# Generational, sex, and socioeconomic inequalities in mental and social wellbeing during the COVID-19 pandemic: prospective longitudinal observational study of five UK cohorts

**DOI:** 10.1017/S0033291722003348

**Published:** 2022-11-08

**Authors:** Darío Moreno-Agostino, Helen L. Fisher, Stephani L. Hatch, Craig Morgan, George B. Ploubidis, Jayati Das-Munshi

**Affiliations:** 1 Centre for Longitudinal Studies, UCL Social Research Institute, University College London, 55-59 Gordon Square, London WC1H 0NU, UK; 2 ESRC Centre for Society and Mental Health, King's College London, Melbourne House, 44-46 Aldwych, London WC2B 4LL, UK; 3 King's College London, Social, Genetic & Developmental Psychiatry Centre, Institute of Psychiatry, Psychology & Neuroscience, 16 De Crespigny Park, London SE5 8AF, UK; 4 Department of Psychological Medicine, King's College London, Institute of Psychiatry, Psychology & Neuroscience, 16 De Crespigny Park, London SE5 8AF, UK; 5 Health Service and Population Research Department, King's College London, Institute of Psychiatry, Psychology & Neuroscience, 16 De Crespigny Park, London SE5 8AF, UK

**Keywords:** longitudinal, mental health, inequalities, cohort, depression, anxiety, loneliness, life satisfaction

## Abstract

**Background:**

Research suggests that there have been inequalities in the impact of the coronavirus disease 2019 (COVID-19) pandemic and related non-pharmaceutical interventions on population mental health. We explored generational, sex, and socioeconomic inequalities during the first year of the pandemic using nationally representative cohorts from the UK.

**Methods:**

We analysed data from 26772 participants from five longitudinal cohorts representing generations born between 1946 and 2000, collected in May 2020, September–October 2020, and February–March 2021 across all five cohorts. We used a multilevel growth curve modelling approach to investigate generational, sex, and socioeconomic differences in levels of anxiety and depressive symptomatology, loneliness, and life satisfaction (LS) over time.

**Results:**

Younger generations had worse levels of mental and social wellbeing throughout the first year of the pandemic. Whereas these generational inequalities narrowed between the first and last observation periods for LS [−0.33 (95% CI −0.51 to −0.15)], they became larger for anxiety [0.22 (0.10, 0.33)]. Generational inequalities in depression and loneliness did not change between the first and last observation periods, but initial depression levels of the youngest cohort were worse than expected if the generational inequalities had not accelerated. Women and those experiencing financial difficulties had worse initial mental and social wellbeing levels than men and those financially living comfortably, respectively, and these gaps did not substantially differ between the first and last observation periods.

**Conclusions:**

By March 2021, mental and social wellbeing inequalities persisted in the UK adult population. Pre-existing generational inequalities may have been exacerbated with the pandemic onset. Policies aimed at protecting vulnerable groups are needed.

## Introduction

Evidence from the initial months of the coronavirus disease 2019 (COVID-19) pandemic suggests that the impact of its onset and of the measures to control its spread have been substantially different not only across different measures of mental and social wellbeing, but also across social groups, contexts, and countries (Gibson, Schneider, Talamonti, & Forshaw, 2021; Prati & Mancini, 2021). A systematic review of 117 studies from 28 different countries found that, among the most usually reported inequality factors, women, younger people, and those in a more disadvantaged socioeconomic position (SEP) generally had worse mental and social wellbeing levels in the initial stages of the pandemic (Gibson et al., 2021).

This is consistent with findings from the UK, where longitudinal evidence comparing mental health outcomes before and after the introduction of the first nationwide lockdown measures on 23 March 2020, has shown that overall levels of distress and anxiety increased in the population, with younger people, women, those in disadvantaged SEP, and those with pre-existing mental health conditions being disproportionately impacted (Kwong et al., 2021; Niedzwiedz et al., 2021; Patel et al., 2022; Pierce et al., 2020; Proto & Quintana-Domeque, 2021). Other studies have focused on monitoring the changes in mental and social wellbeing outcomes as control measures changed (Institute for Government, 2021) during the first months of the pandemic, either as they were gradually eased towards the summer of 2020 (Fancourt, Steptoe, & Bu, 2021; O'Connor et al., 2021; Saunders, Buckman, Fonagy, & Fancourt, 2021; Varga et al., 2021) or as they were reintroduced around October/November 2020 (Ellwardt & Prag, 2021; Patel et al., 2022; Pierce et al., 2021; Stroud & Gutman, 2021; Wetherall et al., 2022). Overall, these studies also suggest that younger people, women, and those in disadvantaged SEP have displayed worse mental and social wellbeing levels or trajectories over time.

However, most of these studies are based either on repeated cross-sectional observations over time –thus unable to explore the change within the same individuals over time– or use convenience or non-probability samples –thus limiting the generalisability of the results. Most of the evidence refers to distress levels, with fewer providing evidence on finer grained outcomes such as anxiety or depressive symptomatology, and even fewer on other relevant mental and social wellbeing outcomes such as loneliness or life satisfaction (LS). Moreover, even if inequalities are reported at the early stages of the pandemic, in most cases it remains unknown whether those inequalities have changed over time. Finally, despite the availability of evidence on inequality factors in isolation, evidence on the interplay of combined inequality factors on those initial/earlier levels or rates of change (e.g. combined generational and sex inequalities) is more limited (Gibson et al., 2021).

By using data collected during the first year of the pandemic, on multiple mental and social wellbeing outcomes, and in five probability samples representing generations born in different periods, we aim to overcome the abovementioned limitations in the available literature. More specifically, we aim to provide evidence that is generalisable to the UK adult population on the generational, sex, and socioeconomic inequalities in the levels and change over time in anxiety and depressive symptomatology, loneliness, and LS during the first year of the COVID-19 pandemic, and to explore generational differences in sex and socioeconomic inequalities. Based on the available literature, we hypothesised that levels across outcomes would be worse in disadvantaged groups (i.e. women, younger adults, and people in more disadvantaged SEP).

## Methods

### Sample and procedure

We used the data from the COVID-19 survey (https://cls.ucl.ac.uk/covid-19-survey/) conducted with participants from five UK cohorts representing different generations: National Survey of Health and Development (NSHD, 1946 cohort) (Wadsworth, Kuh, Richards, & Hardy, 2006), National Child Development Study (NCDS, 1958 cohort) (Power & Elliott, 2006), British Cohort Study (BCS70, 1970 cohort) (Sullivan, Brown, Hamer, & Ploubidis, 2022), Next Steps (NS, 1990 cohort) (Calderwood & Sánchez, 2016), and Millennium Cohort Study (MCS, 2000-02 cohort) (Connelly & Platt, 2014). Detailed information on these cohorts and their designs is available in https://cls.ucl.ac.uk/ for NCDS/1958, BCS70/1970, NS/1990, and MCS/2000-02; and in https://www.nshd.mrc.ac.uk/ for NSHD/1946. The COVID-19 survey (Brown et al., 2021) was designed to collect relevant information around the pandemic impact on the cohort members. Data were collected at three time points: May 2020 (survey wave 1, during the first national lockdown), September–October 2020 (survey wave 2, between the first and second national lockdowns), and February–March 2021 (survey wave 3, during the third national lockdown) (Institute for Government, 2021). Data collection took place via web interviews, supplemented by telephone interviews in survey wave 3 (resulting in larger response rates in this last survey wave). Cohort members were invited to participate via email in survey wave 1, with invitations being also sent via post at survey waves 2 and 3. In this study, we focused on those individuals currently alive and residing in the UK (Appendix S1, Supplementary Material). In MCS, only the data from the main cohort members were included, despite in some cases more than one family member (other sibling/s or parent/s) participating in the survey. Overall response rates with respect to the target populations ranged from 20.8% (survey wave 1) to 31.2% (survey wave 3).

To restore representativeness to the respective target populations due to the differential probability of participating in the COVID surveys, all models were estimated using an inverse probability weighting approach. Where appropriate, non-response weights were combined with the corresponding survey design weights. Participants' internet access was accounted for when deriving the weights to adjust for the potential sample selection effects induced by the survey administration mode (web survey). Further information on the derivation and effectiveness of the weights can be found in the COVID-19 Survey User Guide (Brown et al., 2021).

The COVID-19 Survey was approved by the National Health Service (NHS) Research Ethics Committee, and all participants provided informed consent.

### Measures

A set of common instruments assessing multiple mental and social wellbeing outcomes were used across all cohorts in the COVID-19 surveys. Experiences indicative of anxiety and depression were measured with the 2-item general anxiety disorder (GAD-2) (Kroenke, Spitzer, Williams, Monahan, & Lowe, 2007) and the 2-item Patient Health Questionnaire (PHQ-2) (Kroenke, Spitzer, & Williams, 2003), respectively. Each of these tools include two items on the frequency the respondent has been bothered by experiences of anxiety or depression over the previous two weeks, ranging from 0 (‘Not at all’) to 3 (‘Nearly every day’), which were summed, ranging from 0 (lowest anxiety/depression) to 6 (highest anxiety/depression). Loneliness was measured with the 3-item University of California Los Angeles (UCLA-3) loneliness scale (Hughes, Waite, Hawkley, & Cacioppo, 2004), which includes three items on how frequently the respondents felt they lacked companionship, were left out, or were isolated from others, with three response options: 1 (‘Hardly ever’), 2 (‘Some of the time’), and 3 (‘Often’). The total sum score ranged from 3 (lowest loneliness) to 9 (highest loneliness). Subjective wellbeing was measured with the Office for National Statistics (2018) single question on LS: ‘Overall, how satisfied are you with your life nowadays?’, with response options ranging from 0 (‘Not at all’) to 10 (‘Completely’).

Inequalities in these measures were explored by cohort/generation (NSHD, NCDS, BCS70, NS, MCS), birth sex (man or woman), and SEP. SEP was operationalised using two different indicators: first, the retrospectively assessed self-reported financial situation in the three months prior to the pandemic outbreak (‘pre-pandemic financial situation’ from here onwards), grouped into ‘Living comfortably’, ‘Doing all right’, or ‘Just about getting by’/‘Finding it quite difficult’/‘Finding it very difficult’; and, second, the residential Index of Multiple Deprivation (IMD) rank, grouped into within-country tertiles due to the different methodologies used to derive these ranks across UK countries (Noble et al., 2019), with the first and third tertiles representing the most and least deprived areas, respectively.

### Data analyses

#### Generational, sex, and socioeconomic inequalities during the pandemic

We used a multilevel growth curve modelling approach to analyse differences in the initial levels (at the first survey wave) and change over time (throughout the two additional survey waves) across subgroups in the different outcomes under study. Cohort/generation (a), the above-mentioned subgrouping variables (b), and the interaction among cohort/generation and the subgrouping variables (c) were included in the models to explore generational inequalities, subgroup inequalities, and the interplay between generational and subgroup inequalities in the outcomes' initial levels. In turn, the interaction between each of these three terms (a-c) and time (including linear and quadratic time terms to account for curvilinear trends) were included in the models to explore generational inequalities, subgroup inequalities, and the interplay between generational and subgroup inequalities in the change over time in the outcomes. Separate sets of models were estimated for each outcome and for each subgrouping variable, starting with a set of models with cohort/generation as the only subgrouping variable.

Unadjusted and adjusted models were estimated. Adjusted models included birth sex (in the models by inequality factors different than birth sex); prospectively measured pre-pandemic self-rated health and long-standing illness; highest academic or vocational qualification level achieved, harmonised into National Vocational Qualification (NVQ) levels (Dodgeon & Parsons, 2011), and prospectively measured pre-pandemic housing tenure (except in the models by SEP to avoid overadjustment, since both highest qualification and pre-pandemic housing tenure can be considered indicators of SEP); and household composition. Prospective measures of pre-pandemic LS were available across cohorts and were harmonised and included in the models for LS to adjust for pre-existing inequalities in this outcome. To adjust for potential differences in ill-being outcomes (anxiety, depression, and loneliness), we derived a harmonised measure of psychological distress based on the 28-item General Health Questionnaire (GHQ-28) in NSHD; the Malaise Inventory in NCDS and BCS70; GHQ-12 in NS; and the 6-item Kessler Psychological Distress Scale (K-6) in MCS. Each measure for psychological distress was taken from the most recent pre-pandemic assessment in the respective cohort. Further details on these variables and the analytical approach used can be found in the Appendix S2 and Appendix S3 (Supplementary Material), respectively. To investigate the impact of missing data, unadjusted models were also computed restricting their samples to those of the adjusted models as sensitivity analyses.

Marginal mean estimates and 95% confidence intervals (CIs) of the outcomes were obtained from each of the models (unadjusted and adjusted) and plotted by the different subgroups. Contrasts of marginal predicted levels were performed to obtain estimates (and 95% CIs) of the differences in the adjusted marginal means (*diff*) by cohort/generation, birth sex, and SEP at the beginning of the study. To investigate whether inequalities in these factors had changed throughout the first year of the pandemic, estimates of the change in those initial differences (difference-in-differences, *DID*) by the end of the study period were also obtained.

#### Acceleration of generational inequalities with the pandemic onset

All models explored generational inequalities in the initial levels and rates of change in the outcomes, along with generational differences in the sex and socioeconomic inequalities in the corresponding models. To investigate whether generational inequalities accelerated with the pandemic onset, models were adjusted for potential mediators of the association between generation and the corresponding outcome. A strong attenuation of the generational inequalities observed in the unadjusted models after including these pre-pandemic variables would indicate similar changes across generations with the pandemic onset (in other words, that the mediators may ‘explain away’ the generational differences). Under the assumption that the mediators capture all pre-pandemic differences between generations, any remaining effect may be due to the direct effect of generation on the outcome, suggesting that the pandemic had exacerbated the pre-existing generational inequalities. The regression-based approach to mediation returns an unbiased controlled direct effect under the assumption of no intermediate confounding, no interaction between year of birth/generation and the mediators, and no unmeasured confounding (De Stavola, Daniel, Ploubidis, & Micali, 2015). As sensitivity checks, an additional set of models including additional potential mediators involved in this pathway (i.e. early life determinants) were estimated. These models did not include NS as information in all additional pre-pandemic variables was not available.

As an additional piece of evidence for the potential existence of accelerated generational inequalities in the outcome levels early in the pandemic, we estimated an additional set of models to answer the counterfactual question of when MCS participants ‘should’ have been born to have their mental and social wellbeing initial levels, provided that there was a linear trend in the generational inequalities had not accelerated (in other words, what birth year more closely resembled the marginal mean levels predicted for the MCS cohort if generational inequalities were linear). Such a linear trend would already violate the age trend expected from previous evidence in the UK, where a U-pattern with the lowest mental health levels taking place around midlife (age 35–50) would be expected (Blanchflower, 2021; Gondek et al., 2022).

Further details on the analytical approaches used to investigate the acceleration of generational inequalities are available in Appendix S4 (Supplementary Material).

All analyses and plots were carried out in Stata MP 17 (StataCorp, 2021).

## Results

Data from 26 772 survey participants were analysed, comprising 57 048 observations across the three survey waves of the COVID-19 surveys (Appendix S1, Supplementary Material). As shown in Table 1, most participants were women (52.3–62.4%), in a comfortable pre-pandemic financial situation, living in less deprived areas, and living with their partners or with their partners and others (although members from the two youngest generations, NS and MCS, were more likely to show different characteristics).

**Table 1.** Sociodemographic, economic, and living setting characteristics of the COVID-19 survey participants from the different cohorts

	NSHD, 1946	NCDS, 1958	BCS, 1970	NS, 1990	MCS, 2000
No. of different participants	1567	7691	7042	4971	5501
No. of observations	3983	18 104	15 052	9652	10 257
Survey wave 1 – May 2020	1170	5119	4132	1876	2607
Survey wave 2 – September/October 2020	1488	6228	5236	3609	3229
Survey wave 3 – February/March 2021	1325	6757	5684	4167	4421
Female, *N* (%)	820 (52.3)	4019 (52.3)	3933 (55.9)	3101 (62.4)	3314 (60.2)
Pre-pandemic financial situation, *N* (%)					
Just about getting by/Finding it quite/very difficult	60 (3.8)	800 (10.4)	1144 (16.2)	932 (18.7)	964 (17.5)
Doing all right	312 (19.9)	2531 (32.9)	2719 (38.6)	2015 (40.5)	2344 (42.6)
Living comfortably	986 (62.9)	4055 (52.7)	2909 (41.3)	1827 (36.8)	1825 (33.2)
*Missing*	209 (13.3)	305 (4.0)	270 (3.8)	197 (4.0)	368 (6.7)
IMD tertile, *N* (%)					
1st (most deprived)	333 (21.3)	2252 (29.3)	2087 (29.6)	2141 (43.1)	2137 (38.8)
2^nd^	541 (34.5)	2547 (33.1)	2315 (32.9)	1432 (28.8)	1563 (28.4)
3rd (least deprived)	673 (42.9)	2527 (32.9)	2284 (32.4)	1189 (23.9)	1521 (27.6)
*Missing*	20 (1.3)	365 (4.7)	356 (5.1)	209 (4.2)	280 (5.1)
Pre-pandemic self-reported health, *N* (%)					
Poor	18 (1.1)	296 (3.8)	266 (3.8)	86 (1.7)	54 (1.0)
Fair	147 (9.4)	904 (11.8)	764 (10.8)	318 (6.4)	274 (5.0)
Good	469 (29.9)	2248 (29.2)	1726 (24.5)	1048 (21.1)	1244 (22.6)
Very good	667 (42.6)	2593 (33.7)	2210 (31.4)	1797 (36.1)	2108 (38.3)
Excellent	168 (10.7)	1030 (13.4)	1125 (16.0)	1101 (22.1)	1541 (28.0)
*Missing*	98 (6.3)	620 (8.1)	951 (13.5)	621 (12.5)	280 (5.1)
Pre-pandemic long-standing illness, *N* (%)					
No	640 (40.8)	4796 (62.4)	3938 (55.9)	3529 (71.0)	4283 (77.9)
Yes	824 (52.6)	2263 (29.4)	2155 (30.6)	821 (16.5)	941 (17.1)
*Missing*	103 (6.6)	632 (8.2)	949 (13.5)	621 (12.5)	277 (5.0)
Highest qualification (academic or vocational), *N* (%)^a^					
None	325 (20.7)	493 (6.4)	507 (7.2)	145 (2.9)	513 (9.3)
NVQ-1	99 (6.3)	734 (9.5)	453 (6.4)	278 (5.6)	319 (5.8)
NVQ-2	348 (22.2)	1866 (24.3)	1702 (24.2)	890 (17.9)	1378 (25.0)
NVQ-3	496 (31.7)	1350 (17.6)	967 (13.7)	993 (20.0)	795 (14.5)
NVQ-4	190 (12.1)	2682 (34.9)	2387 (33.9)	1295 (26.1)	1893 (34.4)
NVQ-5	20 (1.3)	393 (5.1)	543 (7.7)	815 (16.4)	276 (5.0)
*Missing*	89 (5.7)	173 (2.2)	483 (6.9)	555 (11.2)	327 (5.9)
Pre-pandemic housing tenure, *N* (%)					
No (rented/rent-free/other arrangement)	83 (5.3)	993 (12.9)	1174 (16.7)	3407 (68.5)	1319 (24.0)
Yes (house owned/partly owned)	1378 (87.9)	6318 (82.1)	4885 (69.4)	996 (20.0)	3811 (69.3)
*Missing*	106 (6.8)	380 (4.9)	983 (14.0)	568 (11.4)	371 (6.7)
Household composition, *N* (%)					
Living alone	368 (23.5)	1884 (24.5)	1047 (14.9)	633 (12.7)	109 (2.0)
Living with partner	1076 (68.7)	3569 (46.4)	1230 (17.5)	1334 (26.8)	140 (2.5)
Living with partner and other(s)	73 (4.7)	1688 (21.9)	3928 (55.8)	1569 (31.6)	172 (3.1)
Other arrangement	47 (3.0)	486 (6.3)	773 (11.0)	1374 (27.6)	4975 (90.4)
*Missing*	3 (0.2)	64 (0.8)	64 (0.9)	61 (1.2)	105 (1.9)
Psychological distress in the most recent available pre-COVID sweep, *N* (%)					
No	1249 (79.7)	5985 (77.8)	4717 (67.0)	3201 (64.4)	4321 (78.5)
Yes	160 (10.2)	883 (11.5)	1030 (14.6)	1111 (22.3)	939 (17.1)
*Missing*	158 (10.1)	823 (10.7)	1295 (18.4)	659 (13.3)	241 (4.4)
LS in the most recent available pre-COVID sweep, *N* (%)					
Low (⩽6)	172 (11.0)	1489 (19.4)	1330 (18.9)	1071 (21.5)	1356 (24.7)
High (>6)	1057 (67.5)	5380 (70.0)	4724 (67.1)	3260 (65.6)	3913 (71.1)
*Missing*	338 (21.6)	822 (10.7)	988 (14.0)	640 (12.9)	232 (4.2)

BCS70, 1970 British Cohort Study; IMD, index of multiple deprivation; NCDS, 1958 National Child Development Study; NVQ, harmonised (based on Dodgeon & Parsons, 2011) qualification categories according to the NVQ system (higher numbers represent higher qualification); NS, 1990 Next Steps; NSHD, 1946 National Survey of Health and Development; MCS, 2000 Millennium Cohort Study.

a In MCS, highest qualification corresponds to the parents' qualification.

### Inequalities during the pandemic

**Fig. 1.**
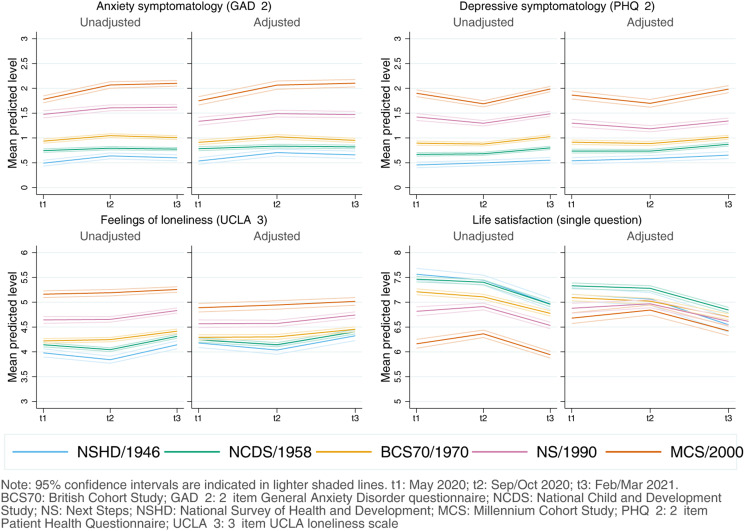
Unadjusted and adjusted marginal mean estimates and 95% CIs estimated for each outcome at each time-point and cohort. Adjusted models included birth sex; highest qualification achieved; prospectively assessed pre-pandemic housing tenure, self-rated health, long-standing illness, psychological distress, and LS (LS only in models for that outcome); and household composition as covariates.

Coefficients, along with the resulting marginal mean estimates and 95% CIs and their corresponding visual depictions, are provided in the Appendices S5–S8 (Supplementary Material), organised by inequality factors. Overall, mental and social wellbeing outcomes worsened throughout the first year of the pandemic. Compared with May 2020, anxiety, depression, loneliness, and LS adjusted marginal mean levels were, on average, *diff*_GAD-2_ = 0.14 (0.11–0.17), *diff*_PHQ-2_ = 0.10 (0.07–0.13), *diff*_UCLA-3_ = 0.15 (0.12–0.19), and *diff*_LS_ = −0.40 (−0.45 to −0.35) points worse, respectively, by February/March 2021, relatively small changes considering the scale ranges. However, the change was not predominantly linear (Fig. 1): increases in anxiety symptomatology were most pronounced between May 2020 (during the first lockdown) and September/October 2020 (between the first and second lockdowns). By contrast, levels of depressive symptomatology, loneliness, and LS seemed to improve by September/October 2020, further worsening by February/March 2021 (during the third lockdown).

#### Inequalities by generation

As depicted in Fig. 1, younger cohorts had higher initial levels of anxiety and depressive symptomatology and were more likely to report loneliness and lower LS compared to older cohorts (further models' results are available in Appendix S5). The differences in the initial levels between the youngest (MCS) and oldest (NSHD) cohorts were, on average, 1.21 (1.10–1.33) for anxiety symptomatology; 1.33 (1.21–1.43) for depressive symptomatology; 0.71 (0.58–0.84) for loneliness; and −0.48 (−0.66 to −0.30) for LS. By the end of the study period, that difference had become *DID*_GAD-2_ = 0.23 (0.12–0.34) points wider for anxiety symptomatology, mainly driven by a greater increase in anxiety symptoms among the youngest cohort between the first and second time-points. The gaps between the youngest and oldest cohorts remained stable for depressive symptomatology [*DID*_PHQ-2_ = 0.005 (−0.10 to 0.11)] and for loneliness [*DID*_UCLA−3_ = −0.02 (−0.14 to 0.11)], despite a temporary improvement in the depressive symptomatology levels among the two youngest cohorts (NS and MCS) by the second time-point. Generational inequalities in LS narrowed by *DID*_LS_ = −0.35 (−0.55 to −0.16) points when comparing the oldest and youngest cohorts.

#### Inequalities by birth sex

**Fig. 2.**
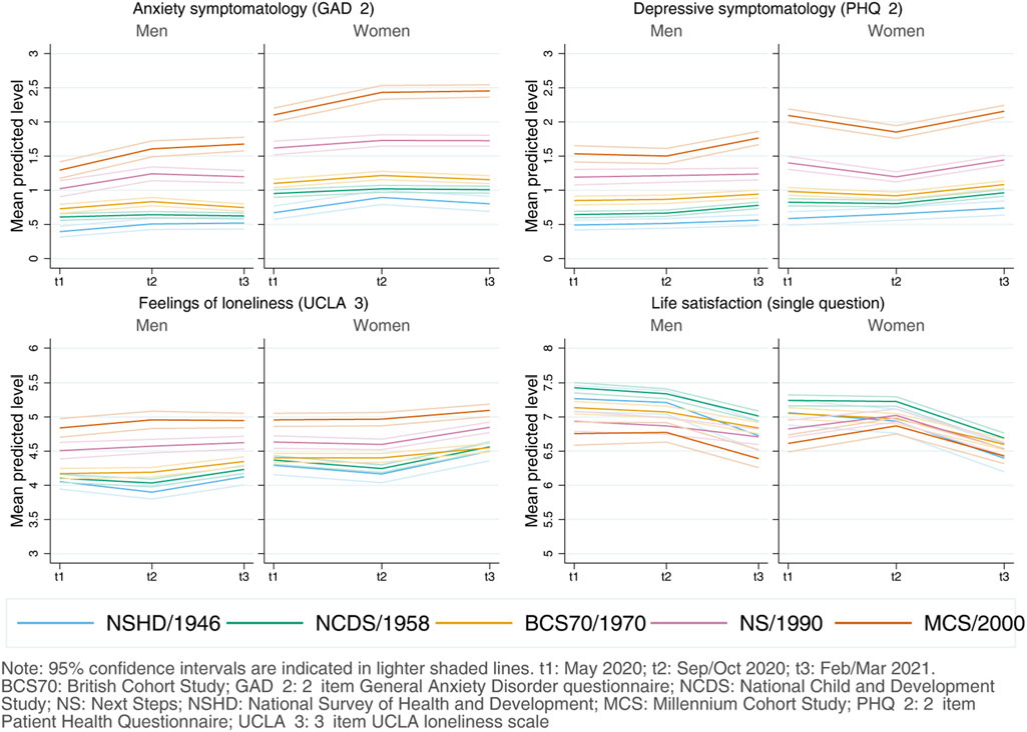
Adjusted marginal mean estimates and 95% CIs estimated for each outcome at each time-point and cohort by birth sex. Models adjusted by highest qualification achieved; prospectively assessed pre-pandemic housing tenure, self-rated health, long-standing illness, psychological distress, and LS (LS only in models for that outcome); and household composition.

Women had, on average, worse initial anxiety symptomatology [*diff*_GAD-2_ = 0.48 (0.43–0.53)], depressive symptomatology [*diff*_PHQ-2_ = 0.24 (0.18–0.29)], loneliness [*diff*_UCLA-3_ = 0.19 (0.14–0.26)], and LS [*diff*_LS_ = −0.15 (−0.23 to −0.07)] levels than men (Fig. 2). However, there was generational variation: women from younger cohorts had higher-than-expected initial levels of anxiety [adjusted unstandardised regression coefficients *B*_NS*woman_ = 0.25 (0.09–0.42); B_MCS*woman_ = 0.46 (0.30–0.62)] and depressive symptomatology [*B*_MCS*woman_ = 0.38 (0.23–0.53)] (Appendix S6). Transient improvements by the second time-point in depressive symptomatology, previously observed among the youngest cohorts, were larger among young women, although with CIs very close or overlapping with zero [*B*_NS*woman*t2_ = −0.17 (−0.35 to −0.02); *B*_MCS*woman*t2_ = −0.17 (−0.35 to 0.01)]. Nevertheless, by the end of the study period, sex inequalities were not substantially different than at the beginning (Appendix S6).

#### Inequalities by SEP

**Fig. 3.**
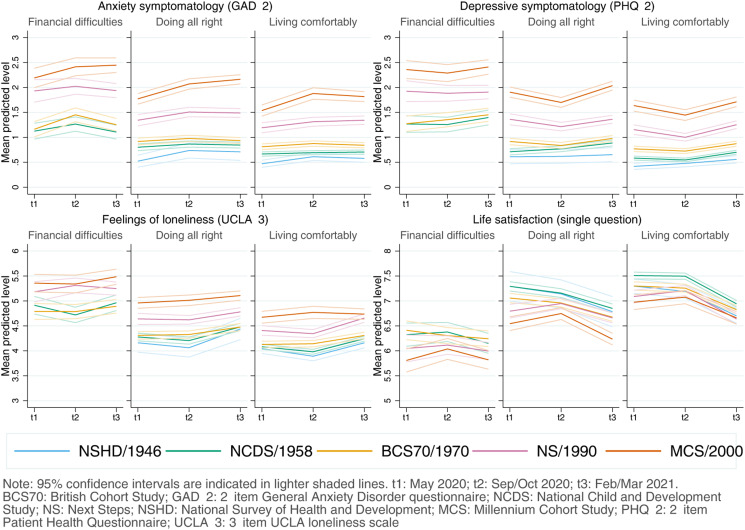
Adjusted marginal mean estimates and 95% CIs estimated for each outcome at each time-point and cohort by pre-pandemic financial situation. 1946 National Survey of Health and Development (NSHD) data for the ‘financial difficulties’ group is not shown due to the small number of cases. Models adjusted by prospectively assessed pre-pandemic self-rated health, long-standing illness, psychological distress, and LS (LS only in models for that outcome); and household composition.

Inequalities by the retrospectively assessed pre-pandemic financial situation drove some of the largest differences in the initial levels in all outcomes, with those in worse situations showing worse initial levels in all outcomes (Fig. 3). As indicated by the lack of significance of the overall interaction terms by SEP and birth year/cohort (Appendix S7), differences observed by pre-pandemic financial situation were not substantially different across generations. The average adjusted difference in the initial levels between those in the worst-off and best-off pre-pandemic financial situations was *diff*_GAD-2_ = 0.56 (0.43–0.69), *diff*_PHQ-2_ = 0.70 (0.56–0.84), *diff*_UCLA-3_ = 0.69 (0.56–0.83), and *diff*_LS_ = −1.02 (−1.21 to −0.82). Overall, there was no substantial change in the differences between those in the worst-off and best-off financial situations by the end of the study period (Appendix S7).

Inequalities by residential IMD tertile were smaller than those by retrospectively assessed pre-pandemic financial situation. In the unadjusted models, people living in more deprived areas had lower mental and social wellbeing levels. However, once the covariates were included in the model, most differences across IMD tertiles attenuated and CIs of the average adjusted difference in the initial levels between those living in areas in the lowest (most deprived) and highest (least deprived) IMD tertiles overlapped or almost overlapped with zero: *diff*_GAD-2_ = −0.02 (−0.08 to 0.05); *diff*_PHQ-2_ = −0.05 (−0.11 to 0.01); *diff*_UCLA-3_ = 0.02 (−0.05 to 0.10); and *diff*_LS_ = −0.10 (−0.20 to −0.004). There was no substantial change in those differences by the end of the study period (Appendix S8) and, although some estimates involving the interaction between IMD tertile and either cohort or the growth parameters were significant (with 0.01 < *p* < 0.05), CIs were in all cases very close to zero.

### Acceleration of generational inequalities with pandemic onset

Although the generational inequalities observed in the outcome levels earlier in the pandemic were attenuated with the inclusion of the potential mediators of those inequalities, they remained evident and were particularly salient in the two youngest cohorts (NS/1990, MCS/2000-02) (Appendix S5). As depicted in Fig. 1, this attenuation was stronger in loneliness and, particularly, in LS. In other words, the included mediators ‘explained away’ part, but not all, of the generational inequalities observed in the outcomes in the earlier stages of the pandemic.

**Fig. 4.**
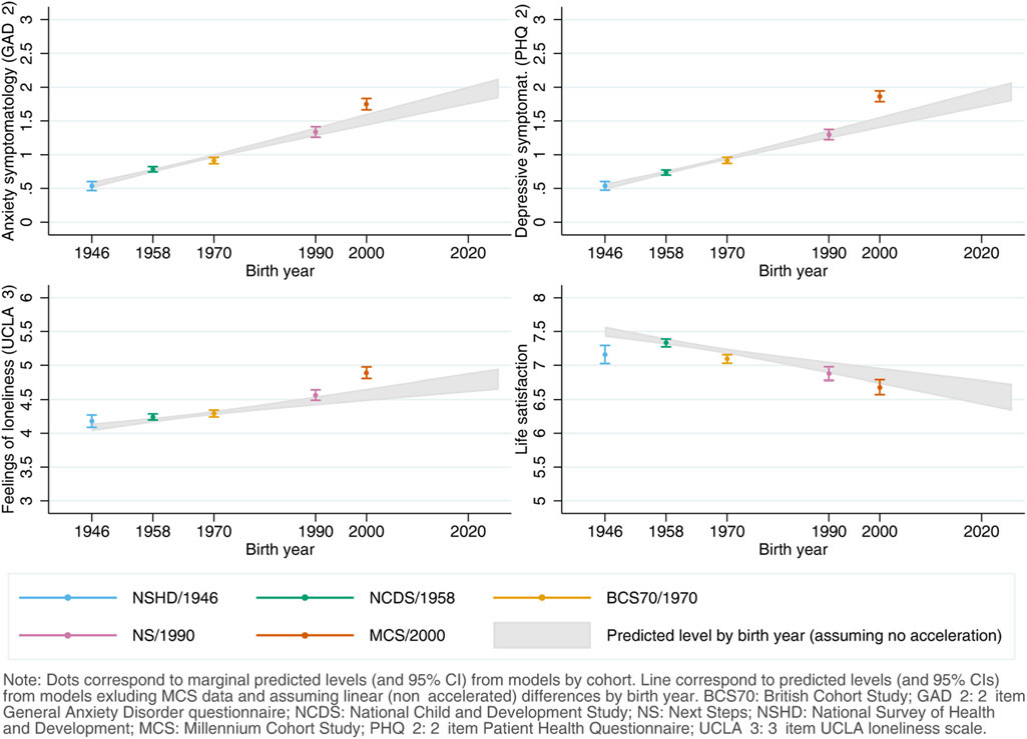
Comparison between marginal predicted initial levels by cohort and marginal predicted initial levels by birth year assuming no acceleration in the differences by birth year and excluding data from the youngest cohort (Millennium Cohort Study, MCS). Models adjusted by birth sex; highest qualification achieved; prospectively assessed pre-pandemic housing tenure, self-rated health, long-standing illness, psychological distress, and LS (LS only in models for that outcome); and household composition.

Figure 4 shows the comparison between the adjusted predicted initial levels for each cohort, obtained from the abovementioned models, and those predicted by birth year from the models estimated excluding MCS data to further explore whether the generational inequalities in the initial levels were accelerating, assuming a linear trend in these inequalities. Although the predictions by the models by birth year for anxiety and depressive symptomatology and loneliness broadly overlapped with those by cohort for the generations born between 1946 and 1990, the youngest cohort's levels were substantially higher than expected even if the increase had been linear over time. This was most noticeable for depressive symptomatology, where the point estimate (*M*_MCS_ = 1.86 (1.78–1.94)) corresponded to that of those born 22 years later, in 2022 (*M*_2022_ = 1.86 (1.73–2.00)). These results were robust to the inclusion of additional pre-pandemic characteristics (online Figure S5.2, Supplementary Material). The adjusted models by birth year did not seem to adequately capture the marginal initial levels of loneliness (which seemed to follow an accelerated increasing trend early on, with levels for those born in 1990 higher than expected from a linear trend) and LS (which seemed to follow a more complex trend).

### Sensitivity analyses

The sensitivity analyses (non-fully adjusted models performed with the same sample as the fully adjusted models) showed very similar results as those with unrestricted samples (Appendices S5–S8).

## Discussion

This study provides evidence on inequalities in initial levels and change over time of several mental and social wellbeing outcomes in the UK population during the first year of the COVID-19 pandemic. These inequalities were observed by generation, birth sex, and pre-pandemic financial situation, with younger cohorts, women, and those in worse financial situations showing worse outcomes. We also found evidence on the interplay between generational and sex inequalities, with anxiety and depressive symptomatology levels being disproportionately high among younger women. Such generational variation did not seem to exist for SEP, which was associated with some of the largest gaps in mental and social wellbeing across cohorts when using pre-pandemic financial status as indicator of the SEP, but with no significant differences across people living in areas at different levels of deprivation. Under the assumption that the variables included in the analyses captured all the pre-pandemic differences in the outcomes across generations, our study also suggests that these generational inequalities may have accelerated with the pandemic onset. The results suggest that the outcome levels (particularly in anxiety and depressive symptomatology) among the youngest cohort are worse than expected had the pandemic not exacerbated the pre-existing generational inequalities.

Younger cohorts systematically showed worse levels in most outcomes at most time-points. By the first year of the pandemic (February/March 2021), the large gaps in depressive symptomatology and loneliness between the youngest and oldest had not reduced, being similar to those at the early stages of the pandemic (May 2020). Our results also suggest that, during the first year of the pandemic, the generational gap narrowed in LS by about 0.35 points (a 3.5% difference considering the 10-point range of the measure) and widened in anxiety symptomatology by about 0.23 points (a 3.8% difference considering the 6-point range of the measure). As operationalised in this study, measures of anxiety may have captured experiences of nervousness and worry. Hence, the widening of generational gaps in anxiety symptomatology may be at least partly explained by sources of concern tied to the younger collective, including worries around disruptions to education and peer socialisation and the uncertainty of when these could end (Dedryver & Knai, 2021; McKinlay, May, Dawes, Fancourt, & Burton, 2022). The inconsistencies of our results compared with a previous study showing improving anxiety levels among UK young (18–29 years old) adults (Fancourt et al., 2021) may be explained by differences between the periods covered in that study –which spanned up to August 2020, when the reinstatement of restrictions by mid-October 2020 had not yet been announced–, and ours –with the second time-point already taking place after that announcement by mid-September 2020 (Institute for Government, 2021). Indeed, evidence from wider follow-up periods (Wetherall et al., 2022) suggests an overall increase in the levels of anxiety and depressive symptomatology between mid-July 2020 (no restrictions) and early October 2021 (restrictions announced). Our study extends this evidence by showing how mental and social wellbeing levels changed across different cohorts throughout the first year of the pandemic using probability samples.

We found evidence suggestive of accelerated generational inequalities in initial anxiety and depression levels (most notably in the latter, based on the further counterfactual analyses) with the pandemic onset, with the youngest generation's initial levels being substantially worse than expected if the generational inequalities had followed a linear trend. Evidence on generational inequalities in high-income English-speaking countries prior to the pandemic was mixed, with evidence suggesting no consistent trend towards worse levels of depression and anxiety disorders (Spiers et al., 2011, 2012); higher levels of psychological distress (reflecting both anxiety and depressive symptomatology) in cohorts born in 1970 compared to those born in 1946 (Gondek et al., 2022; Moreno-Agostino et al., 2022); higher levels of emotional problems among younger cohorts even after accounting for changes in socioeconomic conditions (McElroy, Tibber, Fearon, Patalay, & Ploubidis, 2022); or higher levels of depression among younger cohorts mostly due to a larger report of somatic symptoms (not included in the depression measure used in this study), along with less reports of psychological symptoms (the focus of the depression measure used in this study) (Twenge, 2014). The assumption of a linear trend in the further counterfactual approach to the study of generational inequalities was, therefore, instrumental to accommodate the differences observed across cohorts in their initial levels and to explore whether, as observed, those levels were substantially worse than expected in the youngest cohort. Thus, and considering that the initial levels in this study correspond to the levels in the early stages of the pandemic, our findings are not only consistent with the idea that the pandemic onset had a disproportionate impact among the youngest generation (Kwong et al., 2021; Niedzwiedz et al., 2021; Pierce et al., 2020), but suggest that this impact was even larger than expected if generational inequalities had followed a linear trend. All outcomes remained substantially worse for the youngest cohort by the end of the study period, indicating that, regardless of the source of those inequalities, these have not reduced. Thus, younger generations (i.e. those in their late teenage years, early twenties) may have been the most vulnerable age groups during the pandemic.

A puzzling exception to this general trend was found in the LS levels of those born in 1946, which were lower than expected, especially among women. Although LS levels in high-income English-speaking countries have been found to increase with age after reaching a valley in midlife in cross-sectional studies (Steptoe, Deaton, & Stone, 2015), the longitudinal evidence shows that, with age, a steeper decline in these levels is expected (Jivraj, Nazroo, Vanhoutte, & Chandola, 2014). However, our study suggests that the oldest adults' (NSHD/1946) LS levels were already lower than those of the immediately younger generation (NCDS/1958) at the beginning of the study. Altogether, this may suggest that the pandemic onset had a larger impact on the LS levels of the UK's older adults in their seventies compared to those in their sixties. Further research is needed to analyse the differential impact of the pandemic onset on LS trajectories in the UK's older populations.

Women showed worse levels than men in all outcomes at most time-points, and generational inequalities in anxiety and depressive symptomatology seemed to be substantially larger among women, highlighting the interplay between generational and sex disparities. In line with previous evidence (Fancourt et al., 2021), we found that sex inequalities in anxiety and depressive symptomatology narrowed over time during the initial months of the pandemic; however, those inequalities widened once again by the end of the study period. Very similar results have been reported on distress for young adults (18–29) between April and November 2020 (Stroud & Gutman, 2021). Available evidence on the interplay between generational and sex inequalities is scarcer (Gibson et al., 2021) and is mostly from studies using person-centred methods to identify unobserved subgroups within the population with similar trajectories. These studies, however, point in similar directions as ours, finding that young women were typically more likely to be in worse trajectories of anxiety and depression (Saunders et al., 2021) or psychological distress (Ellwardt & Prag, 2021; Pierce et al., 2021). A potential explanation for these findings is the combination of the abovementioned worries and uncertainty around the restrictions and their implications for the future, together with the larger shares of the household and caring responsibilities that women typically took during restriction periods (Giurge, Whillans, & Yemiscigil, 2021; Xue & McMunn, 2021), increasing the vulnerability to lockdown measures (Xiong et al., 2020). Altogether, these findings may suggest a differential impact of the policies put in place to control the pandemic and highlight the importance of continually monitoring the levels of mental and social wellbeing over time in the population, as trajectories may not follow a linear trend, particularly under the rapidly changing scenarios which took place after the pandemic onset.

Inequalities by SEP varied depending on the indicator used. Large inequalities were observed by pre-pandemic financial situation across all generations, in line with previous research using different indicators of the economic situation in the household (Ellwardt & Prag, 2021; Fancourt et al., 2021; Kwong et al., 2021; O'Connor et al., 2021; Pierce et al., 2020; Saunders et al., 2021). The large, cross-cutting, and relatively homogeneous better mental and social wellbeing levels among those doing financially ‘all right’ compared to those in the worst-off financial situation suggests that this may be an optimal target for public policies aimed at enhancing mental and social wellbeing. We did not find conclusive evidence for such inequalities when using area-level deprivation (IMD) as the SEP indicator. Previous research has reported worse levels of depression and anxiety among those living in more deprived areas in the UK (Kwong et al., 2021). However, the effect sizes associated with area deprivation in that study were small and, like in our study, substantially smaller than those found for financial situation. A potential explanation for these results is that household-centred SEP indicators may be more predictive of an individual's mental and social wellbeing levels than area-level indicators. Moreover, inequalities by area deprivation may themselves vary by other individual and household characteristics beyond cohort/birth year, increasing the risk for having worse mental and social wellbeing levels in combination with other sociodemographic and socioeconomic factors (Pierce et al., 2021).

By using data from five probability samples of the UK population representing generations born in different years (i.e. 1946, 1958, 1970, 1990, and 2000–2002), and using weights to account for both the survey designs and the probability of participating in each of the three COVID-19 survey waves, this study provides evidence that is nationally representative and generalisable to the UK adult population. This evidence covers a wide range of mental and social wellbeing outcomes experienced by people during different phases of the pandemic. Unlike most available evidence, this study provides nuanced evidence on the mental and social wellbeing inequalities in both the initial levels and changes over time. We also accounted for the interplay between generational inequalities and other inequality factors. Moreover, by using data from cohort studies already existing prior to the pandemic onset we could control for pre-pandemic characteristics measured prospectively instead of retrospectively.

However, this study has several limitations. First, the tools used to assess anxiety and depressive symptomatology included only core symptoms, thus providing a relevant but relatively limited picture of that symptomatology. Extended assessment tools such as the GAD-7 or the PHQ-9 could not be included due to logistical limitations and to avoid increasing respondent burden. Second, although our study covers an extended period up to March 2021, the reduced number of repeated assessments limits the granularity of the identified trajectories. Therefore, we acknowledge that there may be additional dynamics taking place between the time-points covered in this study (Ellwardt & Prag, 2021). Third, although the inclusion of multiple interaction terms allowed the trajectories to vary in both their initial levels and growth parameters by the subgrouping variables under study, this resulted in a substantial reduction of power to assess these differences. Future research may use alternative analytical approaches that allow accounting for multiple intersecting social identities (e.g. ethnicity, gender, sexual orientation) tied to social power developed for their use from an intersectional approach (Bauer et al., 2021; Evans, Williams, Onnela, & Subramanian, 2018; Merlo, 2018). Finally, although the analyses were adjusted for relevant prospectively assessed pre-pandemic characteristics, the influence of unmeasured confounding cannot be ruled out, thus limiting the causal interpretation of the findings. This is relevant for the results of anxiety and depressive symptomatology and, particularly, loneliness, where specific pre-pandemic measures of these outcomes were not available. We tried to limit the impact of this shortcoming by adjusting for the existence of pre-pandemic psychological distress, a broad measure of mental ill-health that includes symptoms of anxiety and depression. However, we cannot completely rule out the possibility that at least part of the observed differences in these outcomes are due to pre-existing inequalities.

## Conclusion

Overall, our study suggests that, in the UK adult population, generational, sex, and financial inequalities in mental and social wellbeing outcomes have persisted one year after the first national lockdown. Our study also suggests that generational mental health inequalities may have accelerated with the pandemic onset, with the younger cohorts not only being more impacted than the older cohorts, but also beyond what would have been expected considering existing pre-pandemic generational differences. Moreover, it shows that, during the first year of the pandemic, generational inequalities in anxiety have widened, whereas those in LS have narrowed, although with all generations showing substantially worse levels at the end of the first year of the pandemic than at its early stages. Crucially, our study highlights the importance of exploring the interplay between generational inequalities and those posed by other characteristics such as birth sex. It is critical to keep monitoring mental and social wellbeing levels with an appropriate level of granularity. Measures to support the mental health of the most vulnerable groups in the population may be needed, with a focus on reducing existing gaps and preventing new gaps from appearing.

## Supplementary material

For supplementary material accompanying this paper visit https://doi.org/10.1017/S0033291722003348. click here to view supplementary material
