# Supplementary material for: Generational, sex, and socioeconomic inequalities in mental and social wellbeing during the COVID-19 pandemic: prospective longitudinal observational study of five UK cohorts
Source: Psychol Med. 2022 Nov 8;53(13):6403–14. doi: 10.1017/S0033291722003348 (PMC9874037; doi:10.1017/S0033291722003348)
Supplement: Supplementary file 1 [file S0033291722003348sup001.docx]

**Generational, sex, and socioeconomic inequalities in mental and social wellbeing during the COVID-19 pandemic: prospective longitudinal observational study of five UK cohorts**

**Supplementary Material**

**Table of Contents**

[Appendix S1. Flow diagram of the study sample. 3](#_Toc115772343)

[Appendix S2. Details on variables adjusted for in the growth curve models. 4](#_Toc115772344)

[Appendix S3. Details on the multilevel growth curve models. 6](#_Toc115772345)

[Appendix S4. Details on the analytical strategy used to explore the existence of accelerated generational inequalities in the initial outcome levels. 7](#_Toc115772346)

[Figure S4.1. Directed acyclic graph of the effect of interest. 8](#_Toc115772347)

[Appendix S5. Results by cohort/birth year. 9](#_Toc115772348)

[Table S5.1. Results of multilevel growth curve models by cohort/birth year. 9](#_Toc115772349)

[Table S5.2. Unadjusted and adjusted marginal mean estimates and 95% confidence intervals by cohort/birth year. 11](#_Toc115772350)

[Figure S5.1. Comparison between marginal predicted initial levels by cohort and marginal predicted initial levels by birth year assuming a linear trend in the differences by birth year and excluding data from the youngest cohort (MCS). Unadjusted models. 12](#_Toc115772351)

[Figure S5.2. Comparison between marginal predicted initial levels by cohort and marginal predicted initial levels by birth year assuming a linear trend in the differences by birth year and excluding data from the youngest cohort (MCS). Adjusted (extended) models. 13](#_Toc115772352)

[Appendix S6. Results by birth sex. 14](#_Toc115772353)

[Table S6.1. Results of multilevel growth curve models by birth sex. 14](#_Toc115772354)

[Table S6.2. Unadjusted and adjusted marginal mean estimates and 95% confidence intervals by birth sex. 17](#_Toc115772355)

[Table S6.3. Difference-in-differences (change in difference between men and women between first vs last time point) estimates and 95% confidence intervals. 19](#_Toc115772356)

[Figure S6.1. Unadjusted and adjusted anxiety symptomatology (GAD-2) marginal mean estimates and 95% confidence intervals by birth sex. 20](#_Toc115772357)

[Figure S6.2. Unadjusted and adjusted depressive symptomatology (PHQ-2) marginal mean estimates and 95% confidence intervals by birth sex. 21](#_Toc115772358)

[Figure S6.3. Unadjusted and adjusted loneliness (UCLA-3) marginal mean estimates and 95% confidence intervals by birth sex. 22](#_Toc115772359)

[Figure S6.4. Unadjusted and adjusted life satisfaction marginal mean estimates and 95% confidence intervals by birth sex. 23](#_Toc115772360)

[Appendix S7. Results by socioeconomic position using retrospectively assessed pre-pandemic financial situation as indicator. 24](#_Toc115772361)

[Table S7.1. Results of multilevel growth curve models by pre-pandemic financial situation. 24](#_Toc115772362)

[Table S7.2. Unadjusted and adjusted marginal mean estimates and 95% confidence intervals by pre-pandemic financial situation. 28](#_Toc115772363)

[Table S7.3. Difference-in-differences (change in difference between people in worse vs best pre-pandemic financial situation between first vs last time point) estimates and 95% confidence intervals. 31](#_Toc115772364)

[Figure S7.1. Unadjusted and adjusted anxiety symptomatology (GAD-2) marginal mean estimates and 95% confidence intervals by pre-pandemic financial situation. 32](#_Toc115772365)

[Figure S7.2. Unadjusted and adjusted depressive symptomatology (PHQ-2) marginal mean estimates and 95% confidence intervals by pre-pandemic financial situation. 33](#_Toc115772366)

[Figure S7.3. Unadjusted and adjusted loneliness (UCLA-3) marginal mean estimates and 95% confidence intervals by pre-pandemic financial situation. 34](#_Toc115772367)

[Figure S7.4. Unadjusted and adjusted life satisfaction marginal mean estimates and 95% confidence intervals by pre-pandemic financial situation. 35](#_Toc115772368)

[Appendix S8. Results by socioeconomic position using residential Index of Multiple Deprivation (IMD) tertile as indicator. 36](#_Toc115772369)

[Table S8.1. Results of multilevel growth curve models by IMD tertile. 36](#_Toc115772370)

[TableS8.2. Unadjusted and adjusted marginal mean estimates and 95% confidence intervals by IMD tertile. 41](#_Toc115772371)

[Table S8.3. Difference-in-differences (change in difference between people living in most vs least deprived IMD areas between first vs last time point) estimates and 95% confidence intervals. 44](#_Toc115772372)

[Figure S8.1. Unadjusted and adjusted anxiety symptomatology (GAD-2) marginal mean estimates and 95% confidence intervals by IMD tertile. 45](#_Toc115772373)

[Figure S8.2. Unadjusted and adjusted depressive symptomatology (PHQ-2) marginal mean estimates and 95% confidence intervals by IMD tertile. 46](#_Toc115772374)

[Figure S8.3. Unadjusted and adjusted loneliness (UCLA-3) marginal mean estimates and 95% confidence intervals by IMD tertile. 47](#_Toc115772375)

[Figure S8.4. Unadjusted and adjusted life satisfaction marginal mean estimates and 95% confidence intervals by IMD tertile. 48](#_Toc115772376)

[Supplementary References 49](#_Toc115772377)

# Appendix S1. Flow diagram of the study sample.


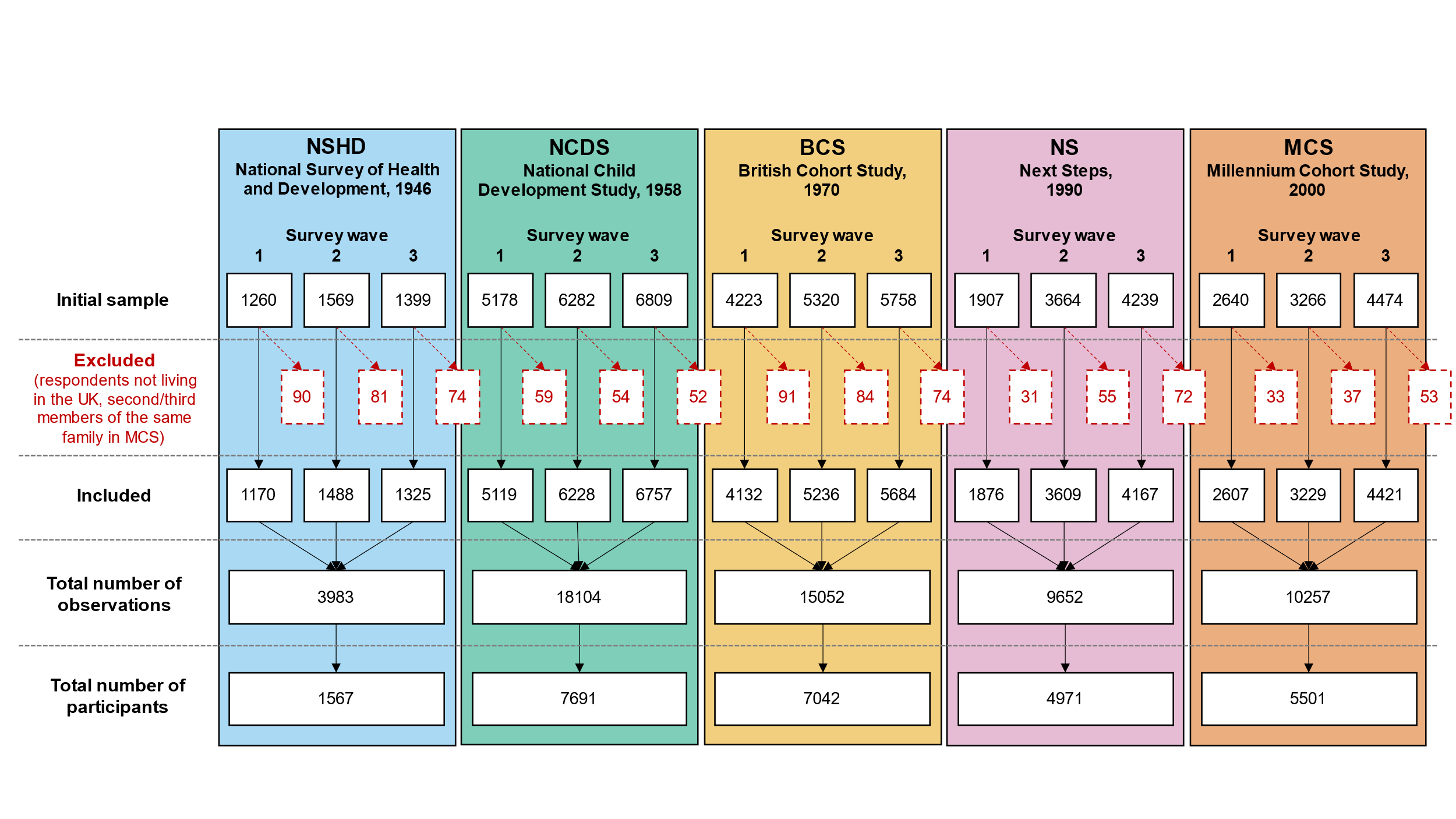


# Appendix S2. Details on variables adjusted for in the growth curve models.

The adjusted models included birth sex (in the models by inequality factors different than birth sex); prospectively measured pre-pandemic self-rated health and long-standing illness; highest academic or vocational qualification level achieved and prospectively measured pre-pandemic housing tenure (except in the models by socioeconomic position [SEP] to avoid overadjustment, since both highest qualification and pre-pandemic housing tenure may be themselves indicators of SEP); and household composition. Prospective measures of pre-pandemic life satisfaction were available across cohorts and were harmonised (see below) and included in the models for life satisfaction to adjust for pre-existing inequalities in this outcome. Such measures were not available for anxiety, depression, and loneliness. Therefore, to adjust for potential differences in these outcomes prior to the pandemic onset, a harmonised (see below) measure of psychological distress in the most recent pre-pandemic assessment was included in the models for anxiety, depression, and loneliness.

Highest qualification was recoded into National Vocational Qualifications (NVQs) equivalents as reported by Dodgeon and Parsons (2011). Data from the youngest cohort (MCS) corresponded to the parents’ highest qualification at the cohort members’ birth, as the degree of variability among cohort members was very small due to their age at the last pre-pandemic data collection (2018, 17 years old).

Prospectively assessed information on pre-pandemic self-rated health and existence of any long-standing illness was available across all cohorts in their most recent pre-pandemic sweeps (NSHD: 2015, NCDS: 2013, BCS70: 2016, NS: 2015, and MCS: 2018). Self-rated health was assessed similarly across cohorts, using the question “In general, would you say your health is 1) excellent, 2) very good, 3) good, 4) fair, or 5) poor?”. Responses were recoded so higher scores represented higher self-rated health levels. This variable was included as a continuous variable as this helped to simplify the estimation of the models without altering the results. The self-reported existence of any long-standing illness was also assessed similarly across cohorts with the question: “Do you have any physical or mental health conditions or illnesses lasting or expected to last 12 months or more?”, recorded and included in the models as a binary variable (yes vs no).

Information prospectively collected on pre-pandemic housing tenure was grouped into owners/part-owners vs renting and any other arrangement and included in the models as a binary variable. Information corresponded to the most recent pre-pandemic sweeps, but in NCDS was supplemented with information from the previous sweep (i.e., 2008) due to a larger number of missing data.

Household composition was classified based on the people living with the respondent at the time of their first participation in the COVID-19 survey study. The four created categories were:

- Alone: if the person reported not living with anyone else.
- Partner: if the person reported living only with their partner.
- Partner and others: if the person reported living with their partner as well as with their children and/or parents.
- Other: if the person reported living in other kind of arrangement (including only with their children and/or parents and with other people.

A binary variable representing the existence of pre-pandemic psychological distress was created using the measures available in the different cohort studies. These measures corresponded to the most recent pre-pandemic assessment available:

- NSHD, 2015, age 69, 28-item General Health Questionnaire (GHQ-28) score equal or greater than 5.
- NCDS, 2008, age 50, Malaise inventory score equal or greater than 4.
- BCS70, 2016-2018, age 46, Malaise inventory score equal or greater than 4.
- NS, 2015, age 25/26, 12-item General Health Questionnaire (GHQ-12) score equal or greater than 4.
- MCS, 2018, age 17, 6-item Kessler Psychological Distress Scale (K-6) score equal or greater than 5.

A binary variable representing whether the person reported a high life satisfaction was created using the measures available in the different cohort studies. Each score was transformed into a common metric of 0 (lowest life satisfaction) to 10 (highest life satisfaction), and a positive score was assigned to all responses of 2/3 (6.67 in a 10-point scale) or higher. These measures corresponded to the most recent pre-pandemic assessment available:

- NSHD, 2009, age 63, “I am satisfied with my life” item from the Satisfaction With Life Scale (SWLS), score equal or greater than 5 in a 1-7 scale.
- NCDS, 2008, age 50, “How satisfied or dissatisfied you are with the way life has turned out so far”, score equal or greater than 7 in a 0-10 scale.
- BCS70, 2016-2018, age 46, “How satisfied or dissatisfied you are with the way life has turned out so far”, score equal or greater than 7 in a 0-10 scale.
- NS, 2015, age 25/26, “How dissatisfied or satisfied are you about the way your life has turned out so far?”, score equal or greater than 4 in a 1-5 scale.

MCS, 2018, age 17, “On the whole, I am satisfied with myself” item from the Young Person Rosenberg Self-esteem Scale, score equal or greater than 4 in a 1-4 scale.

# Appendix S3. Details on the multilevel growth curve models.

We used a multilevel growth curve modelling approach to analyse differences in the initial levels (at the first survey wave) and change over time (throughout the two additional survey waves) across subgroups in the different outcomes under study. In this approach, repeated observations of the outcomes (level 1) are nested within the corresponding individuals (level 2), allowing to simultaneously explore variables related to within- and between-person variability while adjusting for relevant covariates.

As predictors of the between-person variability, we used the cohort/birth year to account for generational differences in the initial levels; the additional subgrouping variables [birth sex and the different indicators of the socioeconomic position (SEP)] to account for between-groups differences in the initial levels, and the interaction among cohort/birth year and the subgrouping variables to account for the interplay between generational effects and the corresponding subgroups in the initial levels.

As predictors of the within-person variability, we used linear and quadratic time terms in order to account for potential curvilinear trends, as well as the interaction between the time terms and the cohort/birth year (to account for generational differences in the change over time), the time terms and the subgrouping variable under study (to account for between-groups differences in the change over time), and the time terms and the interaction between cohort/birth year and the subgrouping variable under study (to account for the interplay between generational effects and the corresponding subgroups in the change over time).

In other words, models studying a subgrouping variable included the three-way interaction terms “time (linear and quadratic) * cohort/birth year * subgrouping variable”.

Separate sets of models were estimated for each outcome and for each subgrouping variable, starting with a set of models with cohort/birth year as the only subgrouping variable. Unadjusted and adjusted models were estimated (see Appendix S2 for details on the variables included in the adjusted models).

All these variables were included in the fixed-effects part of the multilevel models. The random-effects part included the variability in the initial levels (random intercepts) and in the linear change over time (random slopes). These two random effects were allowed to covary freely. Random quadratic slopes were not modelled as their inclusion led to estimation problems.

The overall statistical significance of the main effects of cohort/birth year and the subgrouping variables with more than two levels (i.e., pre-pandemic financial situation and Index of Multiple Deprivation tertile), as well as the interaction terms they were part of, were tested by means of the Wald chi-squared test. Marginal mean estimates and 95% CIs of the outcomes were obtained from each of the models (unadjusted and adjusted) and plotted by the different subgroups.

Participants within the NSHD data who reported experiencing financial difficulties prior to the pandemic onset were not included in the plots due to the small number of cases and subsequent extremely large CIs.

# Appendix S4. Details on the analytical strategy used to explore the existence of accelerated generational inequalities in the initial outcome levels.

All models explored generational inequalities in the initial levels and rates of change in the outcomes, along with generational differences in the sex and socioeconomic inequalities in the corresponding models. To investigate whether generational inequalities accelerated with the pandemic onset, models were adjusted for potential mediators of the association between generation and the corresponding outcome.

A strong attenuation of the generational inequalities observed in the unadjusted models after including these pre-pandemic variables would indicate similar changes across generations with the pandemic onset (in other words, that the mediators may “explain away” the generational differences). Under the assumption that the mediators capture all pre-pandemic differences between generations, any remaining effect may be due to the direct effect of generation on the outcome, suggesting that the pandemic had exacerbated the pre-existing generational inequalities, as depicted in the directed acyclic graph (DAG) below (**Figure S4.1**). The regression-based approach to mediation returns an unbiased controlled direct effect under the assumption of no intermediate confounding, no interaction between year of birth/generation and the mediators, and no unmeasured confounding (De Stavola, Daniel, Ploubidis, & Micali, 2015).

As sensitivity checks, an additional set of models including additional potential mediators involved in this pathway (i.e., early life determinants) were estimated. These models did not include NS as information in all additional pre-pandemic variables was not available.

Early life cognitive ability was operationalised using the results from crystallised ability (verbal ability) at age 10-11 across all four cohorts included in this sub-analysis. The reason for choosing this subdomain of cognitive ability was the availability of assessments of a comparable construct at similar ages across the largest number of cohorts (see McElroy et al., 2021, pages 33 and 35). The measures used corresponded to the verbal test from the National Foundation for Educational Research (NFER) for NSHD and NCDS at age 11, the word similarities test from the British Ability Scales (BAS) for BCS at age 10, and the verbal similarities test from the BAS II for MCS at age 11. The scores were transformed into a common metric ranging from 0 (lowest verbal ability) to 50 (highest verbal ability). Further details on these measures are available in McElroy et al. (2021).

Parental social class during childhood was assessed at ages 10/11 across the four cohorts included in this sub-analysis, representing the highest parental social class from “Professional” to “Unskilled”. Further details on the harmonisation of this variable is available in Dodgeon et al. (2011).

To further account for additional early life socioeconomic conditions, information on housing tenure during childhood was included, generated using prospectively assessed information obtained in the corresponding early life sweeps of the corresponding cohorts. A harmonised variable indicating whether the cohort member was in a rented or owned accommodation in early childhood (ages 2, 7, and 5 in NSHD, NCDS, and BCS70, respectively) and at age 10/11 had been previously generated for NSHD, NCDS, and BCS70 by Wood, Stafford, and O’Neill (2019). The same procedure was used to generate the harmonised variable in MCS, using data from ages 5 and 11.

A binary variable representing the existence of psychological distress in the second to most recent available pre-pandemic assessment was obtained for each of the four cohorts included in this sub-analysis:

- NSHD, 2009, age 63, GHQ-28 score equal or greater than 5.
- NCDS, 2000, age 42, Malaise inventory score equal or greater than 4.
- BCS70, 2012, age 42, Malaise inventory score equal or greater than 4.
- MCS, 2015, age 14, Short mood and Feelings Questionnaire (SMFQ) score equal or greater than 12.

The results of the multilevel growth curve models suggested seemingly higher than expected initial levels in the youngest cohort (MCS) if strictly attending at the birth year separation across cohorts, where a larger gap between BCS70 and NS could be expected due to the relatively wider chronological separation (20 years), but not between NS and MCS (10 years). It is important to note that assuming such a linear trend would already violate the age trend expected from previous evidence in the UK, where a U-pattern with the lowest mental health levels taking place around midlife (age 35-50) would be expected (Blanchflower, 2021; Gondek et al., 2022), and therefore the worst outcome levels could have been expected in the NS and BCS70 cohorts.

In light of the results, we laid out an additional analytical strategy to further investigate the existence of accelerated generational inequalities in the outcome levels early in the pandemic. Since the cohort that seemed to be diverging from a linear trend was MCS, we estimated an additional set of models to answer the counterfactual question of when MCS participants ‘should’ have been born to have their mental and social wellbeing initial levels, provided that there was a linear trend in the generational inequalities (in other words, what birth year more closely resembled the marginal mean levels predicted for the MCS cohort if generational inequalities were linear).

To do so, these models were estimated excluding MCS data and using birth year (centred at 1946, the birth year of NSHD cohort) as a continuous variable to explore linear generational inequalities instead of cohort as a categorical variable. Marginal predictions were obtained from these models for every birth year from 1946 to 2020 and were then compared to those from the original models to identify the birth year that provided the closest match to each of the cohorts.

## Figure S4.1. Directed acyclic graph of the effect of interest.

**
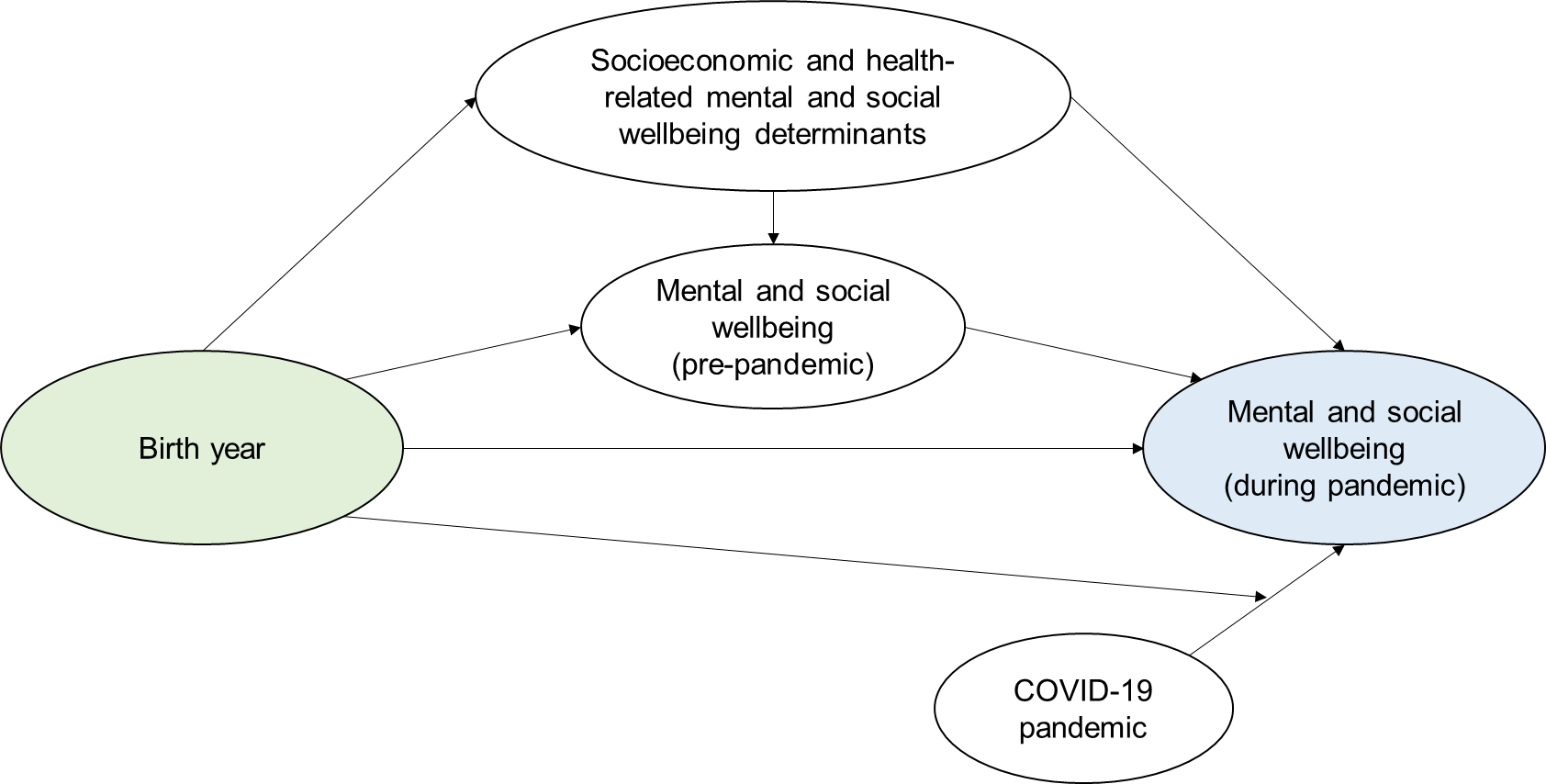
**

# Appendix S5. Results by cohort/birth year.

## Table S5.1. Results of multilevel growth curve models by cohort/birth year.

|  | **Anxiety symptomatology (GAD-2)** | | | **Depressive symptomatology (PHQ-2)** | | | **Feelings of loneliness (UCLA-3)** | | | **Life satisfaction (ONS single question)** | | |
| --- | --- | --- | --- | --- | --- | --- | --- | --- | --- | --- | --- | --- |
| **Unadjusted models** |  |  |  |  |  |  |  |  |  |  |  |  |
| N participants | 25,635 |  |  | 25,633 |  |  | 25,664 |  |  | 25,722 |  |  |
| N observations | 54,140 |  |  | 54,123 |  |  | 54,208 |  |  | 54,444 |  |  |
|  | ***B* (95% CI)** | **χ2** | ***p*** | ***B* (95% CI)** | **χ2** | ***p*** | ***B* (95% CI)** | **χ2** | ***p*** | ***B* (95% CI)** | **χ2** | ***p*** |
| Time (linear) | 0.08 (0.02, 0.14) |  | 0.005 | -0.04 (-0.09, 0.01) |  | 0.139 | -0.28 (-0.34, -0.22) |  | <0.001 | 0.13 (0.04, 0.21) |  | 0.003 |
| Time (quad) | -0.03 (-0.06, -0.01) |  | 0.014 | 0.05 (0.03, 0.08) |  | <0.001 | 0.18 (0.15, 0.21) |  | <0.001 | -0.19 (-0.23, -0.15) |  | <0.001 |
| Cohort (ref. NCDS) |  | 1,107.6 | <0.001 |  | 1472.1 | <0.001 |  | 823.3 | <0.001 |  | 698.7 | <0.001 |
| NSHD | -0.25 (-0.32, -0.18) |  |  | -0.21 (-0.28, -0.15) |  |  | -0.16 (-0.26, -0.07) |  |  | 0.10 (-0.03, 0.23) |  |  |
| BCS | 0.19 (0.14, 0.25) |  |  | 0.23 (0.17, 0.28) |  |  | 0.08 (0.02, 0.14) |  |  | -0.25 (-0.33, -0.17) |  |  |
| NS | 0.73 (0.65, 0.81) |  |  | 0.75 (0.68, 0.83) |  |  | 0.50 (0.42, 0.58) |  |  | -0.65 (-0.75, -0.54) |  |  |
| MCS | 1.03 (0.95, 1.11) |  |  | 1.23 (1.16, 1.31) |  |  | 1.02 (0.94, 1.10) |  |  | -1.30 (-1.41, -1.20) |  |  |
| Linear change * cohort (ref. NCDS) |  | 24.7 | <0.001 |  | 50.5 | <0.001 |  | 41.0 | <0.001 |  | 32.7 | <0.001 |
| NSHD | 0.16 (0.04, 0.28) |  |  | 0.07 (-0.04, 0.19) |  |  | -0.08 (-0.22, 0.07) |  |  | -0.07 (-0.28, 0.15) |  |  |
| BCS | 0.10 (0.01, 0.20) |  |  | -0.06 (-0.15, 0.02) |  |  | 0.24 (0.14, 0.34) |  |  | -0.12 (-0.25, 0.01) |  |  |
| NS | 0.11 (-0.03, 0.25) |  |  | -0.25 (-0.38, -0.12) |  |  | 0.21 (0.08, 0.34) |  |  | 0.21 (0.03, 0.38) |  |  |
| MCS | 0.34 (0.19, 0.48) |  |  | -0.43 (-0.57, -0.28) |  |  | 0.29 (0.15, 0.44) |  |  | 0.38 (0.20, 0.57) |  |  |
| Quad. change * cohort (ref. NCDS) |  | 11.3 | 0.024 |  | 53.7 | <0.001 |  | 49.2 | <0.001 |  | 23.0 | <0.001 |
| NSHD | -0.06 (-0.11, 0.00) |  |  | -0.05 (-0.10, 0.01) |  |  | 0.04 (-0.03, 0.10) |  |  | 0.01 (-0.09, 0.11) |  |  |
| BCS | -0.04 (-0.08, 0.00) |  |  | 0.03 (-0.01, 0.07) |  |  | -0.11 (-0.16, -0.07) |  |  | 0.08 (0.02, 0.14) |  |  |
| NS | -0.03 (-0.09, 0.04) |  |  | 0.11 (0.05, 0.16) |  |  | -0.10 (-0.16, -0.04) |  |  | -0.05 (-0.12, 0.03) |  |  |
| MCS | -0.10 (-0.16, -0.03) |  |  | 0.20 (0.14, 0.27) |  |  | -0.17 (-0.23, -0.10) |  |  | -0.12 (-0.20, -0.04) |  |  |
| **Adjusted models** |  |  |  |  |  |  |  |  |  |  |  |  |
| N participants | 21,262 |  |  | 21,256 |  |  | 21,798 |  |  | 21,088 |  |  |
| N observations | 46,136 |  |  | 46,124 |  |  | 47,087 |  |  | 45,802 |  |  |
|  | ***B* (95% CI)** | **χ2** | ***p*** | ***B* (95% CI)** | **χ2** | ***p*** | ***B* (95% CI)** | **χ2** | ***p*** | ***B* (95% CI)** | **χ2** | ***p*** |
| Time (linear) | 0.09 (0.02, 0.15) |  | 0.007 | -0.07 (-0.13, -0.01) |  | 0.015 | -0.28 (-0.34, -0.21) |  | <0.001 | 0.14 (0.05, 0.23) |  | 0.002 |
| Time (quad) | -0.03 (-0.06, -0.01) |  | 0.020 | 0.07 (0.04, 0.10) |  | <0.001 | 0.18 (0.15, 0.21) |  | <0.001 | -0.19 (-0.24, -0.15) |  | <0.001 |
| Cohort (ref. NCDS) |  | 547.6 | <0.001 |  | 722.2 | <0.001 |  | 190.8 | <0.001 |  | 126.4 | <0.001 |
| NSHD | -0.25 (-0.32, -0.17) |  |  | -0.20 (-0.27, -0.13) |  |  | -0.06 (-0.16, 0.04) |  |  | -0.17 (-0.31, -0.03) |  |  |
| BCS | 0.13 (0.07, 0.19) |  |  | 0.18 (0.12, 0.24) |  |  | 0.05 (-0.01, 0.12) |  |  | -0.24 (-0.32, -0.15) |  |  |
| NS | 0.55 (0.46, 0.64) |  |  | 0.56 (0.48, 0.65) |  |  | 0.32 (0.23, 0.42) |  |  | -0.45 (-0.57, -0.34) |  |  |
| MCS | 0.96 (0.87, 1.06) |  |  | 1.13 (1.04, 1.22) |  |  | 0.65 (0.55, 0.75) |  |  | -0.65 (-0.78, -0.52) |  |  |
| Linear change * cohort (ref. NCDS) |  | 26.9 | <0.001 |  | 27.4 | <0.001 |  | 33.3 | <0.001 |  | 17.8 | 0.001 |
| NSHD | 0.19 (0.06, 0.32) |  |  | 0.10 (-0.03, 0.23) |  |  | -0.08 (-0.24, 0.09) |  |  | -0.02 (-0.28, 0.24) |  |  |
| BCS | 0.12 (0.01, 0.22) |  |  | -0.03 (-0.13, 0.06) |  |  | 0.21 (0.11, 0.32) |  |  | -0.09 (-0.24, 0.05) |  |  |
| NS | 0.16 (0.01, 0.30) |  |  | -0.18 (-0.32, -0.04) |  |  | 0.20 (0.06, 0.34) |  |  | 0.17 (-0.01, 0.35) |  |  |
| MCS | 0.37 (0.21, 0.53) |  |  | -0.32 (-0.47, -0.17) |  |  | 0.32 (0.17, 0.48) |  |  | 0.31 (0.11, 0.51) |  |  |
| Quad. change * cohort (ref. NCDS) |  | 13.6 | 0.009 |  | 27.8 | <0.001 |  | 38.7 | <0.001 |  | 13.9 | 0.008 |
| NSHD | -0.07 (-0.14, -0.01) |  |  | -0.06 (-0.12, 0.01) |  |  | 0.03 (-0.05, 0.11) |  |  | -0.02 (-0.15, 0.12) |  |  |
| BCS | -0.06 (-0.11, -0.01) |  |  | 0.01 (-0.04, 0.05) |  |  | -0.11 (-0.16, -0.06) |  |  | 0.07 (0.01, 0.14) |  |  |
| NS | -0.05 (-0.12, 0.01) |  |  | 0.07 (0.00, 0.13) |  |  | -0.10 (-0.16, -0.04) |  |  | -0.03 (-0.11, 0.05) |  |  |
| MCS | -0.11 (-0.18, -0.03) |  |  | 0.16 (0.08, 0.23) |  |  | -0.17 (-0.25, -0.10) |  |  | -0.10 (-0.19, -0.01) |  |  |
| **Sensitivity models** |  |  |  |  |  |  |  |  |  |  |  |  |
| N participants | 21,262 |  |  | 21,256 |  |  | 21,798 |  |  | 21,088 |  |  |
| N observations | 46,136 |  |  | 46,124 |  |  | 47,087 |  |  | 45,802 |  |  |
|  | ***B* (95% CI)** | **χ2** | ***p*** | ***B* (95% CI)** | **χ2** | ***p*** | ***B* (95% CI)** | **χ2** | ***p*** | ***B* (95% CI)** | **χ2** | ***p*** |
| Time (linear) | 0.09 (0.03, 0.15) |  | 0.004 | -0.06 (-0.12, -0.01) |  | 0.028 | -0.27 (-0.33, -0.20) |  | <0.001 | 0.13 (0.03, 0.22) |  | 0.007 |
| Time (quad) | -0.03 (-0.06, -0.01) |  | 0.018 | 0.07 (0.04, 0.09) |  | <0.001 | 0.18 (0.15, 0.21) |  | <0.001 | -0.19 (-0.23, -0.15) |  | <0.001 |
| Cohort (ref. NCDS) |  | 966.6 | <0.001 |  | 1253.6 | <0.001 |  | 694.3 | <0.001 |  | 557.2 | <0.001 |
| NSHD | -0.24 (-0.31, -0.17) |  |  | -0.21 (-0.28, -0.14) |  |  | -0.13 (-0.23, -0.03) |  |  | 0.03 (-0.12, 0.17) |  |  |
| BCS | 0.19 (0.13, 0.25) |  |  | 0.23 (0.17, 0.28) |  |  | 0.06 (0.00, 0.13) |  |  | -0.25 (-0.34, -0.16) |  |  |
| NS | 0.73 (0.64, 0.81) |  |  | 0.75 (0.67, 0.83) |  |  | 0.51 (0.42, 0.59) |  |  | -0.64 (-0.75, -0.53) |  |  |
| MCS | 1.02 (0.94, 1.11) |  |  | 1.19 (1.11, 1.28) |  |  | 1.00 (0.92, 1.09) |  |  | -1.24 (-1.35, -1.13) |  |  |
| Linear change * cohort (ref. NCDS) |  | 24.9 | <0.001 |  | 29.2 | <0.001 |  | 35.1 | <0.001 |  | 19 | <0.001 |
| NSHD | 0.19 (0.06, 0.32) |  |  | 0.10 (-0.03, 0.23) |  |  | -0.08 (-0.25, 0.08) |  |  | 0.00 (-0.27, 0.26) |  |  |
| BCS | 0.11 (0.01, 0.22) |  |  | -0.04 (-0.13, 0.06) |  |  | 0.21 (0.10, 0.32) |  |  | -0.09 (-0.23, 0.06) |  |  |
| NS | 0.14 (-0.01, 0.29) |  |  | -0.19 (-0.33, -0.05) |  |  | 0.20 (0.06, 0.34) |  |  | 0.19 (0.00, 0.37) |  |  |
| MCS | 0.36 (0.20, 0.52) |  |  | -0.34 (-0.49, -0.18) |  |  | 0.31 (0.16, 0.47) |  |  | 0.33 (0.13, 0.54) |  |  |
| Quad. change * cohort (ref. NCDS) |  | 13.1 | 0.011 |  | 28.7 | <0.001 |  | 38.2 | <0.001 |  | 13.9 | 0.008 |
| NSHD | -0.08 (-0.14, -0.01) |  |  | -0.06 (-0.12, 0.01) |  |  | 0.03 (-0.05, 0.11) |  |  | -0.02 (-0.16, 0.11) |  |  |
| BCS | -0.06 (-0.11, -0.01) |  |  | 0.01 (-0.04, 0.05) |  |  | -0.11 (-0.16, -0.06) |  |  | 0.07 (0.01, 0.14) |  |  |
| NS | -0.05 (-0.11, 0.02) |  |  | 0.07 (0.01, 0.13) |  |  | -0.10 (-0.16, -0.04) |  |  | -0.04 (-0.12, 0.05) |  |  |
| MCS | -0.11 (-0.18, -0.03) |  |  | 0.16 (0.09, 0.23) |  |  | -0.17 (-0.24, -0.10) |  |  | -0.10 (-0.19, 0.00) |  |  |

*Note.* Adjusted models included birth sex; highest qualification achieved; prospectively assessed pre-pandemic housing tenure, self-rated health, long-standing illness, psychological distress, and life satisfaction (life satisfaction only in models for that outcome); and household composition as covariates. Sensitivity models correspond to the unadjusted models after restricting the analytical sample to that of the adjusted models. BCS: British Cohort Study, 1970 birth cohort; GAD-2: 2-item General Anxiety Disorder questionnaire; MCS: Millennium Cohort Study, 2000 birth cohort; NCDS: National Child and Development Study, 1958 birth cohort; NS: Next Steps, 1990 cohort; NSHD: National Survey of Health and Development, 1946 birth cohort; ONS: UK Office for National Statistics; PHQ-2: 2-item Patient Health Questionnaire; UCLA-3: 3-item UCLA loneliness scale. χ2: Wald test performed to assess the overall statistical significance of the interaction terms; all χ2 statistics in this table have 4 degrees of freedom.

## Table S5.2. Unadjusted and adjusted marginal mean estimates and 95% confidence intervals by cohort/birth year.

|  |  | **Anxiety symptomatology (GAD-2)** | **Depressive symptomatology (PHQ-2)** | **Feelings of loneliness (UCLA-3)** | **Life satisfaction (ONS single question)** |
| --- | --- | --- | --- | --- | --- |
| **Cohort** | **Survey wave** | **Unadjusted marginal mean (95% CI)** | **Unadjusted marginal mean (95% CI)** | **Unadjusted marginal mean (95% CI)** | **Unadjusted marginal mean (95% CI)** |
| NSHD | 1 | 0.49 (0.43, 0.55) | 0.46 (0.40, 0.51) | 3.98 (3.90, 4.06) | 7.56 (7.44, 7.68) |
| NSHD | 2 | 0.64 (0.58, 0.70) | 0.50 (0.45, 0.55) | 3.84 (3.77, 3.91) | 7.44 (7.34, 7.55) |
| NSHD | 3 | 0.60 (0.54, 0.66) | 0.55 (0.50, 0.61) | 4.14 (4.06, 4.22) | 6.97 (6.85, 7.08) |
| NCDS | 1 | 0.75 (0.71, 0.78) | 0.67 (0.64, 0.70) | 4.14 (4.10, 4.18) | 7.46 (7.41, 7.52) |
| NCDS | 2 | 0.79 (0.76, 0.83) | 0.68 (0.65, 0.71) | 4.04 (4.01, 4.08) | 7.40 (7.35, 7.45) |
| NCDS | 3 | 0.78 (0.74, 0.81) | 0.80 (0.77, 0.83) | 4.31 (4.27, 4.35) | 6.96 (6.91, 7.01) |
| BCS | 1 | 0.94 (0.89, 0.98) | 0.90 (0.86, 0.94) | 4.22 (4.17, 4.27) | 7.21 (7.15, 7.27) |
| BCS | 2 | 1.05 (1.01, 1.09) | 0.88 (0.84, 0.92) | 4.25 (4.20, 4.29) | 7.11 (7.06, 7.16) |
| BCS | 3 | 1.01 (0.97, 1.05) | 1.03 (0.99, 1.07) | 4.42 (4.37, 4.46) | 6.78 (6.72, 6.83) |
| NS | 1 | 1.48 (1.40, 1.55) | 1.42 (1.35, 1.49) | 4.64 (4.57, 4.71) | 6.82 (6.73, 6.91) |
| NS | 2 | 1.61 (1.55, 1.67) | 1.30 (1.24, 1.35) | 4.65 (4.60, 4.71) | 6.91 (6.85, 6.98) |
| NS | 3 | 1.62 (1.57, 1.68) | 1.49 (1.44, 1.54) | 4.83 (4.78, 4.89) | 6.53 (6.47, 6.60) |
| MCS | 1 | 1.78 (1.71, 1.85) | 1.90 (1.83, 1.97) | 5.16 (5.09, 5.23) | 6.16 (6.07, 6.25) |
| MCS | 2 | 2.07 (2.00, 2.13) | 1.69 (1.63, 1.75) | 5.19 (5.13, 5.26) | 6.36 (6.29, 6.44) |
| MCS | 3 | 2.10 (2.05, 2.16) | 1.99 (1.93, 2.04) | 5.26 (5.20, 5.31) | 5.94 (5.88, 6.01) |
| **Cohort** | **Survey wave** | **Adjusted marginal mean (95% CI)** | **Adjusted marginal mean (95% CI)** | **Adjusted marginal mean (95% CI)** | **Adjusted marginal mean (95% CI)** |
| NSHD | 1 | 0.54 (0.47, 0.60) | 0.54 (0.48, 0.60) | 4.18 (4.09, 4.27) | 7.16 (7.03, 7.29) |
| NSHD | 2 | 0.71 (0.64, 0.78) | 0.58 (0.52, 0.65) | 4.04 (3.95, 4.12) | 7.07 (6.94, 7.19) |
| NSHD | 3 | 0.66 (0.58, 0.73) | 0.65 (0.58, 0.72) | 4.32 (4.23, 4.42) | 6.55 (6.40, 6.70) |
| NCDS | 1 | 0.78 (0.74, 0.82) | 0.74 (0.70, 0.77) | 4.24 (4.20, 4.29) | 7.33 (7.27, 7.39) |
| NCDS | 2 | 0.84 (0.80, 0.87) | 0.74 (0.70, 0.77) | 4.14 (4.10, 4.19) | 7.28 (7.23, 7.33) |
| NCDS | 3 | 0.82 (0.78, 0.86) | 0.87 (0.84, 0.91) | 4.41 (4.36, 4.45) | 6.84 (6.79, 6.90) |
| BCS | 1 | 0.91 (0.87, 0.96) | 0.92 (0.87, 0.96) | 4.29 (4.24, 4.35) | 7.09 (7.03, 7.16) |
| BCS | 2 | 1.02 (0.98, 1.07) | 0.89 (0.85, 0.93) | 4.30 (4.25, 4.35) | 7.02 (6.96, 7.08) |
| BCS | 3 | 0.95 (0.91, 0.99) | 1.01 (0.97, 1.06) | 4.45 (4.41, 4.50) | 6.71 (6.65, 6.77) |
| NS | 1 | 1.34 (1.26, 1.42) | 1.30 (1.22, 1.38) | 4.57 (4.49, 4.64) | 6.88 (6.78, 6.98) |
| NS | 2 | 1.49 (1.42, 1.56) | 1.19 (1.12, 1.25) | 4.57 (4.51, 4.64) | 6.97 (6.89, 7.05) |
| NS | 3 | 1.47 (1.41, 1.53) | 1.34 (1.28, 1.40) | 4.74 (4.68, 4.81) | 6.61 (6.54, 6.69) |
| MCS | 1 | 1.75 (1.66, 1.83) | 1.86 (1.78, 1.95) | 4.89 (4.80, 4.98) | 6.68 (6.57, 6.79) |
| MCS | 2 | 2.07 (1.98, 2.15) | 1.70 (1.62, 1.78) | 4.94 (4.86, 5.03) | 6.84 (6.74, 6.94) |
| MCS | 3 | 2.10 (2.03, 2.18) | 1.98 (1.91, 2.06) | 5.02 (4.94, 5.09) | 6.42 (6.33, 6.52) |

*Note.* Adjusted models included birth sex; highest qualification achieved; prospectively assessed pre-pandemic housing tenure, self-rated health, long-standing illness, psychological distress, and life satisfaction (life satisfaction only in models for that outcome); and household composition as covariates. BCS: British Cohort Study, 1970 birth cohort; GAD-2: 2-item General Anxiety Disorder questionnaire; MCS: Millennium Cohort Study, 2000 birth cohort; NCDS: National Child and Development Study, 1958 birth cohort; NS: Next Steps, 1990 cohort; NSHD: National Survey of Health and Development, 1946 birth cohort; ONS: UK Office for National Statistics; PHQ-2: 2-item Patient Health Questionnaire; UCLA-3: 3-item UCLA loneliness scale. Survey wave 1: May 2020; survey wave 2: September/October 2020; survey wave 3: February/March 2021.

## Figure S5.1. Comparison between marginal predicted initial levels by cohort and marginal predicted initial levels by birth year assuming a linear trend in the differences by birth year and excluding data from the youngest cohort (MCS). Unadjusted models.


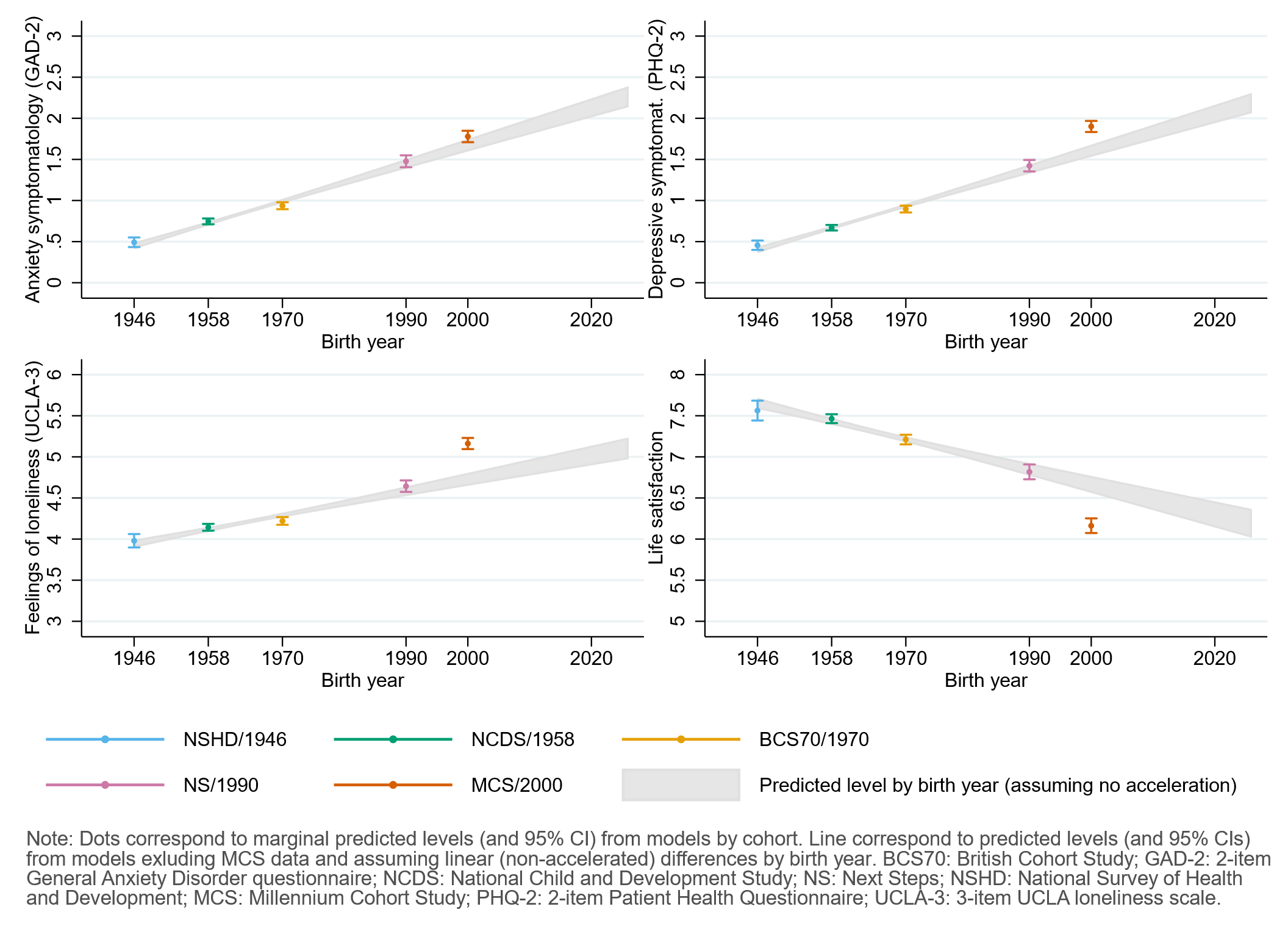


## Figure S5.2. Comparison between marginal predicted initial levels by cohort and marginal predicted initial levels by birth year assuming a linear trend in the differences by birth year and excluding data from the youngest cohort (MCS). Adjusted (extended) models.

Models adjusted by birth sex; highest qualification achieved; prospectively assessed pre-pandemic housing tenure, self-rated health, long-standing illness, psychological distress, and life satisfaction (life satisfaction only in models for that outcome); and household composition; as well as early life cognitive ability, parental social class and housing tenure during childhood, and the existence of psychological distress in the second to most recent available pre-pandemic cohort assessment.


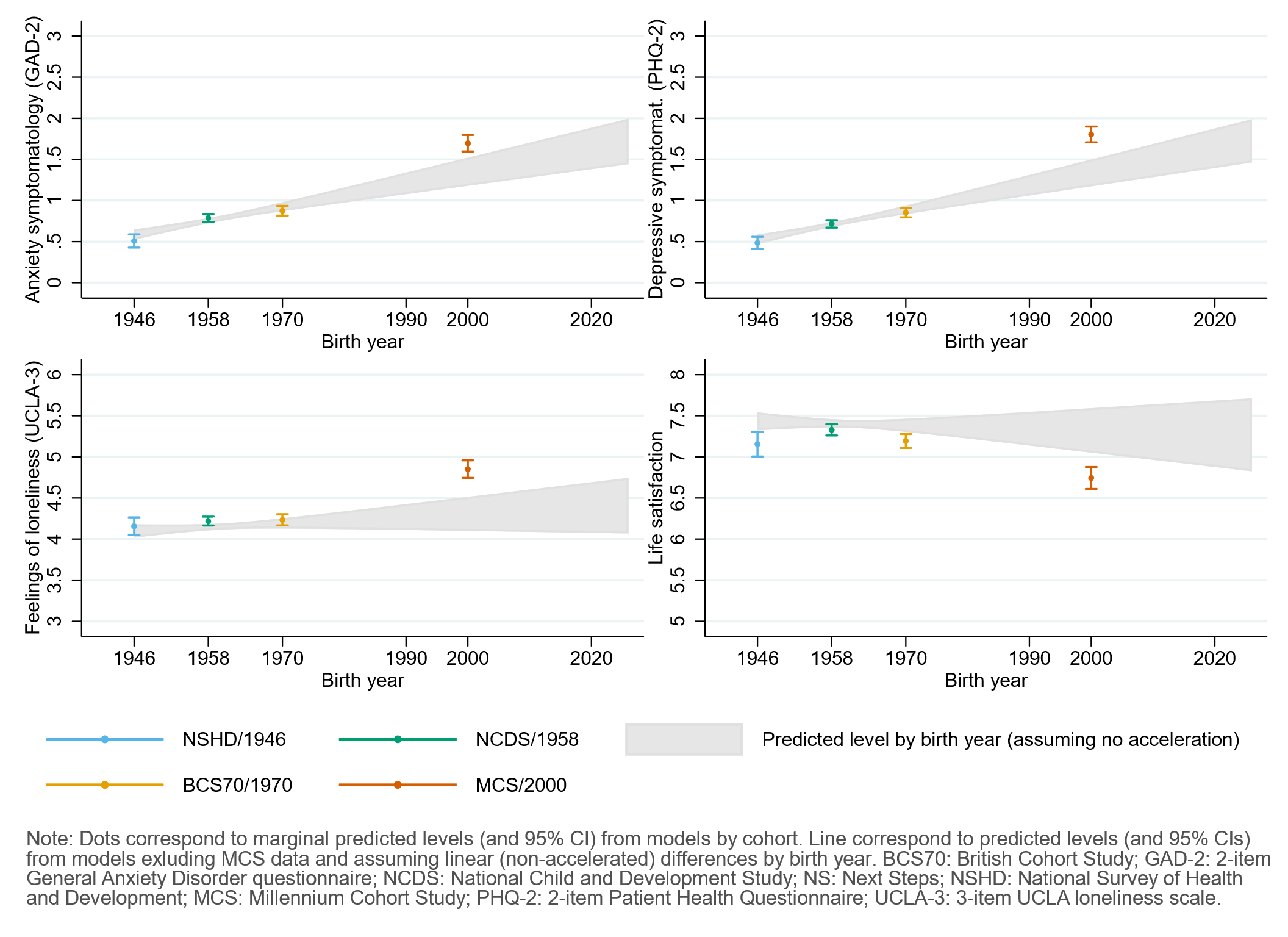


# Appendix S6. Results by birth sex.

## Table S6.1. Results of multilevel growth curve models by birth sex.

|  | **Anxiety symptomatology (GAD-2)** | | | **Depressive symptomatology (PHQ-2)** | | | **Feelings of loneliness (UCLA-3)** | | | **Life satisfaction (ONS single question)** | | |
| --- | --- | --- | --- | --- | --- | --- | --- | --- | --- | --- | --- | --- |
| **Unadjusted models** |  |  |  |  |  |  |  |  |  |  |  |  |
| N participants | 25,635 |  |  | 25,633 |  |  | 25,664 |  |  | 25,722 |  |  |
| N observations | 54,140 |  |  | 54,123 |  |  | 54,208 |  |  | 54,444 |  |  |
|  | ***B* (95% CI)** | **χ2** | ***p*** | ***B* (95% CI)** | **χ2** | ***p*** | ***B* (95% CI)** | **χ2** | ***p*** | ***B* (95% CI)** | **χ2** | ***p*** |
| Time (linear) | 0.05 (-0.02, 0.12) |  | 0.157 | -0.01 (-0.08, 0.06) |  | 0.719 | -0.23 (-0.31, -0.14) |  | <0.001 | 0.09 (-0.02, 0.21) |  | 0.113 |
| Time (quad) | -0.02 (-0.06, 0.01) |  | 0.192 | 0.04 (0.01, 0.07) |  | 0.020 | 0.15 (0.11, 0.19) |  | <0.001 | -0.15 (-0.21, -0.10) |  | <0.001 |
| Cohort (ref. NCDS) |  | 278.7 | <0.001 |  | 456.5 | <0.001 |  | 343.2 | <0.001 |  | 220.2 | <0.001 |
| NSHD | -0.23 (-0.31, -0.15) |  |  | -0.21 (-0.29, -0.14) |  |  | -0.19 (-0.31, -0.08) |  |  | 0.17 (-0.02, 0.36) |  |  |
| BCS | 0.21 (0.13, 0.28) |  |  | 0.27 (0.19, 0.34) |  |  | 0.09 (0.00, 0.18) |  |  | -0.31 (-0.43, -0.19) |  |  |
| NS | 0.55 (0.43, 0.67) |  |  | 0.72 (0.60, 0.84) |  |  | 0.60 (0.46, 0.73) |  |  | -0.68 (-0.85, -0.52) |  |  |
| MCS | 0.65 (0.53, 0.76) |  |  | 0.89 (0.78, 1.01) |  |  | 1.05 (0.92, 1.19) |  |  | -1.11 (-1.29, -0.94) |  |  |
| Linear change * cohort (ref. NCDS) |  | 17.4 | 0.002 |  | 1.6 | 0.809 |  | 25.1 | <0.001 |  | 2.9 | 0.569 |
| NSHD | 0.15 (-0.01, 0.30) |  |  | 0.02 (-0.11, 0.15) |  |  | -0.10 (-0.28, 0.07) |  |  | 0.00 (-0.31, 0.31) |  |  |
| BCS | 0.10 (-0.04, 0.23) |  |  | 0.00 (-0.13, 0.12) |  |  | 0.23 (0.09, 0.37) |  |  | -0.12 (-0.31, 0.06) |  |  |
| NS | 0.25 (0.03, 0.46) |  |  | 0.01 (-0.19, 0.22) |  |  | 0.30 (0.08, 0.52) |  |  | -0.10 (-0.36, 0.16) |  |  |
| MCS | 0.40 (0.18, 0.63) |  |  | -0.13 (-0.35, 0.09) |  |  | 0.34 (0.10, 0.59) |  |  | 0.10 (-0.21, 0.40) |  |  |
| Quad. change * cohort (ref. NCDS) |  | 7.9 | 0.100 |  | 4.2 | 0.384 |  | 27.0 | <0.001 |  | 5.9 | 0.209 |
| NSHD | -0.05 (-0.13, 0.02) |  |  | -0.02 (-0.08, 0.04) |  |  | 0.05 (-0.03, 0.13) |  |  | -0.03 (-0.17, 0.12) |  |  |
| BCS | -0.04 (-0.10, 0.02) |  |  | 0.00 (-0.06, 0.06) |  |  | -0.09 (-0.15, -0.02) |  |  | 0.07 (-0.01, 0.16) |  |  |
| NS | -0.08 (-0.17, 0.01) |  |  | -0.02 (-0.11, 0.07) |  |  | -0.15 (-0.25, -0.06) |  |  | 0.09 (-0.02, 0.21) |  |  |
| MCS | -0.11 (-0.22, -0.01) |  |  | 0.09 (-0.01, 0.19) |  |  | -0.18 (-0.30, -0.07) |  |  | -0.03 (-0.16, 0.11) |  |  |
| Birth sex (ref. Male) | 0.46 (0.39, 0.53) |  |  | 0.28 (0.21, 0.35) |  | <0.001 | 0.39 (0.30, 0.47) |  | <0.001 | -0.22 (-0.33, -0.11) |  | <0.001 |
| Linear change * birth sex (ref. Male) | 0.05 (-0.06, 0.17) |  | 0.357 | -0.05 (-0.16, 0.05) |  | 0.323 | -0.10 (-0.23, 0.02) |  | 0.106 | 0.07 (-0.10, 0.24) |  | 0.430 |
| Quad. change * birth sex (ref. Male) | -0.02 (-0.07, 0.03) |  | 0.437 | 0.03 (-0.02, 0.08) |  | 0.250 | 0.07 (0.01, 0.13) |  | 0.018 | -0.07 (-0.15, 0.01) |  | 0.069 |
| Cohort (ref. NCDS) * birth sex (ref. Male) |  | 67.8 | <0.001 |  | 52.1 | <0.001 |  | 8.7 | 0.070 |  | 12.7 | 0.013 |
| NSHD | -0.05 (-0.18, 0.09) |  |  | 0.00 (-0.13, 0.13) |  |  | 0.06 (-0.12, 0.24) |  |  | -0.14 (-0.40, 0.12) |  |  |
| BCS | -0.06 (-0.17, 0.05) |  |  | -0.09 (-0.20, 0.02) |  |  | -0.05 (-0.17, 0.08) |  |  | 0.11 (-0.05, 0.27) |  |  |
| NS | 0.22 (0.06, 0.38) |  |  | 0.01 (-0.15, 0.17) |  |  | -0.21 (-0.38, -0.05) |  |  | 0.09 (-0.12, 0.31) |  |  |
| MCS | 0.56 (0.40, 0.71) |  |  | 0.49 (0.34, 0.64) |  |  | -0.12 (-0.28, 0.05) |  |  | -0.25 (-0.47, -0.04) |  |  |
| Linear change * cohort (ref. NCDS) * birth sex (ref. Male) |  | 3.0 | 0.555 |  | 18.8 | <0.001 |  | 1.6 | 0.817 |  | 13.1 | 0.011 |
| NSHD | 0.01 (-0.22, 0.25) |  |  | 0.10 (-0.12, 0.32) |  |  | 0.05 (-0.23, 0.33) |  | 0.738 | -0.12 (-0.55, 0.31) |  | 0.584 |
| BCS | 0.01 (-0.17, 0.20) |  |  | -0.10 (-0.28, 0.08) |  |  | 0.02 (-0.18, 0.22) |  | 0.822 | 0.00 (-0.25, 0.26) |  | 0.973 |
| NS | -0.22 (-0.50, 0.05) |  |  | -0.41 (-0.68, -0.15) |  |  | -0.14 (-0.41, 0.14) |  | 0.326 | 0.49 (0.15, 0.83) |  | 0.005 |
| MCS | -0.07 (-0.36, 0.22) |  |  | -0.42 (-0.71, -0.13) |  |  | -0.06 (-0.36, 0.25) |  | 0.718 | 0.42 (0.03, 0.80) |  | 0.034 |
| Quad. change * cohort (ref. NCDS) * birth sex (ref. Male) |  | 2.4 | 0.671 |  | 18.7 | <0.001 |  | 3.9 | 0.416 |  | 12 | 0.017 |
| NSHD | -0.01 (-0.12, 0.10) |  |  | -0.04 (-0.15, 0.06) |  |  | -0.02 (-0.15, 0.11) |  |  | 0.07 (-0.13, 0.27) |  |  |
| BCS | 0.00 (-0.09, 0.08) |  |  | 0.05 (-0.03, 0.13) |  |  | -0.05 (-0.14, 0.05) |  |  | 0.01 (-0.11, 0.12) |  |  |
| NS | 0.09 (-0.03, 0.22) |  |  | 0.20 (0.08, 0.32) |  |  | 0.08 (-0.04, 0.20) |  |  | -0.22 (-0.37, -0.07) |  |  |
| MCS | 0.02 (-0.11, 0.16) |  |  | 0.17 (0.03, 0.30) |  |  | 0.02 (-0.12, 0.16) |  |  | -0.13 (-0.31, 0.04) |  |  |
| **Adjusted models** |  |  |  |  |  |  |  |  |  |  |  |  |
| N participants | 21,262 |  |  | 21,259 |  |  | 21,288 |  |  | 21,088 |  |  |
| N observations | 46,136 |  |  | 46,124 |  |  | 46,197 |  |  | 45,802 |  |  |
|  | ***B* (95% CI)** | **χ2** | ***p*** | ***B* (95% CI)** | **χ2** | ***p*** | ***B* (95% CI)** | **χ2** | ***p*** | ***B* (95% CI)** | **χ2** | ***p*** |
| Time (linear) | 0.06 (-0.02, 0.14) |  | 0.135 | -0.03 (-0.10, 0.05) |  | 0.489 | -0.20 (-0.29, -0.11) |  | <0.001 | 0.03 (-0.09, 0.15) |  | 0.639 |
| Time (quad) | -0.03 (-0.06, 0.01) |  | 0.158 | 0.05 (0.01, 0.08) |  | 0.009 | 0.13 (0.09, 0.17) |  | <0.001 | -0.12 (-0.18, -0.06) |  | <0.001 |
| Cohort (ref. NCDS) |  | 176.8 | <0.001 |  | 277.5 | <0.001 |  | 117.6 | <0.001 |  | 73.5 | <0.001 |
| NSHD | -0.21 (-0.30, -0.12) |  |  | -0.15 (-0.24, -0.07) |  |  | -0.05 (-0.17, 0.07) |  |  | -0.16 (-0.36, 0.04) |  |  |
| BCS | 0.12 (0.04, 0.20) |  |  | 0.21 (0.13, 0.28) |  |  | 0.07 (-0.03, 0.16) |  |  | -0.29 (-0.41, -0.18) |  |  |
| NS | 0.41 (0.29, 0.54) |  |  | 0.55 (0.42, 0.67) |  |  | 0.40 (0.27, 0.54) |  |  | -0.49 (-0.66, -0.33) |  |  |
| MCS | 0.69 (0.55, 0.82) |  |  | 0.89 (0.76, 1.02) |  |  | 0.74 (0.58, 0.89) |  |  | -0.67 (-0.86, -0.48) |  |  |
| Linear change * cohort (ref. NCDS) |  | 14.4 | 0.006 |  | 2.4 | 0.663 |  | 19.4 | <0.001 |  | 2.0 | 0.730 |
| NSHD | 0.10 (-0.07, 0.27) |  |  | 0.04 (-0.09, 0.17) |  |  | -0.14 (-0.34, 0.06) |  |  | 0.13 (-0.23, 0.49) |  |  |
| BCS | 0.14 (-0.01, 0.29) |  |  | 0.01 (-0.13, 0.16) |  |  | 0.15 (0.00, 0.31) |  |  | -0.01 (-0.21, 0.20) |  |  |
| NS | 0.29 (0.06, 0.51) |  |  | 0.05 (-0.17, 0.26) |  |  | 0.27 (0.05, 0.50) |  |  | -0.05 (-0.33, 0.23) |  |  |
| MCS | 0.37 (0.13, 0.61) |  |  | -0.15 (-0.40, 0.09) |  |  | 0.38 (0.12, 0.65) |  |  | 0.19 (-0.14, 0.52) |  |  |
| Quad. change * cohort (ref. NCDS) |  | 7.9 | 0.095 |  | 6.0 | 0.196 |  | 20.4 | <0.001 |  | 4.8 | 0.305 |
| NSHD | -0.02 (-0.10, 0.06) |  |  | -0.03 (-0.10, 0.03) |  |  | 0.06 (-0.04, 0.16) |  |  | -0.10 (-0.27, 0.08) |  |  |
| BCS | -0.07 (-0.14, 0.00) |  |  | -0.02 (-0.08, 0.05) |  |  | -0.06 (-0.14, 0.01) |  |  | 0.03 (-0.06, 0.13) |  |  |
| NS | -0.10 (-0.21, 0.00) |  |  | -0.05 (-0.14, 0.05) |  |  | -0.14 (-0.24, -0.04) |  |  | 0.07 (-0.05, 0.20) |  |  |
| MCS | -0.09 (-0.21, 0.02) |  |  | 0.10 (-0.01, 0.21) |  |  | -0.20 (-0.32, -0.08) |  |  | -0.08 (-0.23, 0.07) |  |  |
| Birth sex (ref. Male) | 0.34 (0.27, 0.41) |  | <0.001 | 0.18 (0.11, 0.25) |  | <0.001 | 0.27 (0.19, 0.35) |  | <0.001 | -0.19 (-0.29, -0.08) |  | 0.001 |
| Linear change * birth sex (ref. Male) | 0.05 (-0.08, 0.17) |  | 0.446 | -0.08 (-0.20, 0.03) |  | 0.148 | -0.14 (-0.28, -0.01) |  | 0.032 | 0.21 (0.03, 0.39) |  | 0.020 |
| Quad. change * birth sex (ref. Male) | -0.01 (-0.07, 0.04) |  | 0.642 | 0.04 (-0.01, 0.10) |  | 0.114 | 0.09 (0.03, 0.15) |  | 0.005 | -0.14 (-0.23, -0.06) |  | 0.001 |
| Cohort (ref. NCDS) * birth sex (ref. Male) |  | 45.1 | <0.001 |  | 31.6 | <0.001 |  | 4.8 | 0.311 |  | 2.0 | 0.739 |
| NSHD | -0.07 (-0.21, 0.07) |  |  | -0.08 (-0.22, 0.05) |  |  | -0.03 (-0.22, 0.16) |  |  | -0.02 (-0.30, 0.26) |  |  |
| BCS | 0.03 (-0.08, 0.14) |  |  | -0.05 (-0.15, 0.06) |  |  | -0.03 (-0.16, 0.09) |  |  | 0.10 (-0.05, 0.26) |  |  |
| NS | 0.25 (0.09, 0.42) |  |  | 0.03 (-0.13, 0.19) |  |  | -0.14 (-0.31, 0.02) |  |  | 0.07 (-0.14, 0.28) |  |  |
| MCS | 0.46 (0.31, 0.62) |  |  | 0.38 (0.23, 0.53) |  |  | -0.15 (-0.32, 0.02) |  |  | 0.04 (-0.18, 0.26) |  |  |
| Linear change * cohort (ref. NCDS) * birth sex (ref. Male) |  | 5.1 | 0.278 |  | 11.0 | 0.027 |  | 3.3 | 0.514 |  | 8.8 | 0.065 |
| NSHD | 0.17 (-0.09, 0.43) |  |  | 0.13 (-0.12, 0.38) |  |  | 0.13 (-0.19, 0.45) |  |  | -0.29 (-0.80, 0.23) |  |  |
| BCS | -0.04 (-0.25, 0.17) |  |  | -0.08 (-0.28, 0.11) |  |  | 0.12 (-0.10, 0.33) |  |  | -0.17 (-0.45, 0.12) |  |  |
| NS | -0.23 (-0.53, 0.07) |  |  | -0.37 (-0.65, -0.08) |  |  | -0.11 (-0.39, 0.18) |  |  | 0.35 (-0.02, 0.72) |  |  |
| MCS | 0.01 (-0.31, 0.33) |  |  | -0.25 (-0.56, 0.06) |  |  | -0.09 (-0.41, 0.24) |  |  | 0.17 (-0.25, 0.59) |  |  |
| Quad. change * cohort (ref. NCDS) * birth sex (ref. Male) |  | 5.1 | 0.275 |  | 10.4 | 0.034 |  | 5.4 | 0.249 |  | 8.0 | 0.090 |
| NSHD | -0.10 (-0.22, 0.03) |  |  | -0.05 (-0.17, 0.08) |  |  | -0.05 (-0.20, 0.11) |  |  | 0.15 (-0.12, 0.41) |  |  |
| BCS | 0.02 (-0.08, 0.12) |  |  | 0.04 (-0.05, 0.13) |  |  | -0.08 (-0.18, 0.02) |  |  | 0.08 (-0.05, 0.21) |  |  |
| NS | 0.09 (-0.05, 0.22) |  |  | 0.18 (0.06, 0.31) |  |  | 0.06 (-0.07, 0.19) |  |  | -0.16 (-0.32, 0.01) |  |  |
| MCS | -0.02 (-0.17, 0.13) |  |  | 0.08 (-0.06, 0.23) |  |  | 0.03 (-0.12, 0.19) |  |  | -0.01 (-0.20, 0.18) |  |  |
| **Sensitivity models** |  |  |  |  |  |  |  |  |  |  |  |  |
| N participants | 21,262 |  |  | 21,259 |  |  | 21,288 |  |  | 21,088 |  |  |
| N observations | 46,136 |  |  | 46,124 |  |  | 46,197 |  |  | 45,802 |  |  |
|  | ***B* (95% CI)** | **χ2** | ***p*** | ***B* (95% CI)** | **χ2** | ***p*** | ***B* (95% CI)** | **χ2** | ***p*** | ***B* (95% CI)** | **χ2** | ***p*** |
| Time (linear) | 0.07 (-0.01, 0.15) |  | 0.102 | -0.02 (-0.09, 0.06) |  | 0.673 | -0.19 (-0.28, -0.10) |  | <0.001 | 0.01 (-0.11, 0.14) |  | 0.831 |
| Time (quad) | -0.03 (-0.07, 0.01) |  | 0.141 | 0.04 (0.01, 0.08) |  | 0.014 | 0.13 (0.09, 0.17) |  | <0.001 | -0.12 (-0.18, -0.06) |  | <0.001 |
| Cohort (ref. NCDS) |  | 252.8 | <0.001 |  | 412.5 | <0.001 |  | 301.0 | <0.001 |  | 192.8 | <0.001 |
| NSHD | -0.21 (-0.30, -0.12) |  |  | -0.19 (-0.27, -0.10) |  |  | -0.16 (-0.29, -0.04) |  |  | 0.10 (-0.12, 0.31) |  |  |
| BCS | 0.19 (0.11, 0.27) |  |  | 0.27 (0.19, 0.35) |  |  | 0.10 (0.01, 0.20) |  |  | -0.34 (-0.47, -0.22) |  |  |
| NS | 0.56 (0.43, 0.68) |  |  | 0.73 (0.61, 0.86) |  |  | 0.60 (0.47, 0.74) |  |  | -0.69 (-0.86, -0.53) |  |  |
| MCS | 0.69 (0.57, 0.81) |  |  | 0.91 (0.79, 1.04) |  |  | 1.06 (0.92, 1.20) |  |  | -1.11 (-1.29, -0.93) |  |  |
| Linear change * cohort (ref. NCDS) |  | 14.4 | 0.006 |  | 1.9 | 0.750 |  | 19.5 | <0.001 |  | 2.0 | 0.735 |
| NSHD | 0.09 (-0.08, 0.26) |  |  | 0.03 (-0.10, 0.16) |  |  | -0.16 (-0.36, 0.04) |  |  | 0.15 (-0.22, 0.51) |  |  |
| BCS | 0.13 (-0.02, 0.28) |  |  | 0.00 (-0.15, 0.14) |  |  | 0.13 (-0.03, 0.29) |  |  | 0.02 (-0.19, 0.22) |  |  |
| NS | 0.28 (0.05, 0.51) |  |  | 0.05 (-0.17, 0.26) |  |  | 0.29 (0.06, 0.52) |  |  | -0.06 (-0.34, 0.22) |  |  |
| MCS | 0.39 (0.14, 0.63) |  |  | -0.14 (-0.39, 0.10) |  |  | 0.39 (0.12, 0.65) |  |  | 0.18 (-0.16, 0.51) |  |  |
| Quad. change * cohort (ref. NCDS) |  | 8.1 | 0.087 |  | 5.3 | 0.254 |  | 20.7 | <0.001 |  | 4.3 | 0.370 |
| NSHD | -0.02 (-0.10, 0.06) |  |  | -0.03 (-0.09, 0.03) |  |  | 0.06 (-0.04, 0.16) |  |  | -0.10 (-0.28, 0.08) |  |  |
| BCS | -0.07 (-0.13, 0.00) |  |  | -0.01 (-0.08, 0.06) |  |  | -0.06 (-0.13, 0.02) |  |  | 0.02 (-0.07, 0.12) |  |  |
| NS | -0.10 (-0.21, 0.00) |  |  | -0.05 (-0.15, 0.05) |  |  | -0.15 (-0.25, -0.04) |  |  | 0.08 (-0.05, 0.20) |  |  |
| MCS | -0.10 (-0.22, 0.01) |  |  | 0.09 (-0.02, 0.21) |  |  | -0.20 (-0.32, -0.08) |  |  | -0.07 (-0.22, 0.08) |  |  |
| Birth sex (ref. Male) | 0.45 (0.38, 0.52) |  | <0.001 | 0.29 (0.22, 0.36) |  | <0.001 | 0.39 (0.30, 0.47) |  | <0.001 | -0.25 (-0.37, -0.14) |  | <0.001 |
| Linear change * birth sex (ref. Male) | 0.04 (-0.08, 0.16) |  | 0.519 | -0.09 (-0.21, 0.02) |  | 0.106 | -0.15 (-0.29, -0.02) |  | 0.022 | 0.21 (0.03, 0.39) |  | 0.021 |
| Quad. change * birth sex (ref. Male) | -0.01 (-0.07, 0.05) |  | 0.699 | 0.05 (-0.01, 0.10) |  | 0.086 | 0.09 (0.03, 0.15) |  | 0.003 | -0.14 (-0.23, -0.06) |  | 0.001 |
| Cohort (ref. NCDS) * birth sex (ref. Male) |  | 49.3 | <0.001 |  | 38.4 | <0.001 |  | 9.3 | 0.054 |  | 11.4 | 0.022 |
| NSHD | -0.06 (-0.20, 0.08) |  |  | -0.04 (-0.18, 0.10) |  |  | 0.06 (-0.14, 0.25) |  |  | -0.12 (-0.42, 0.17) |  |  |
| BCS | -0.02 (-0.14, 0.10) |  |  | -0.10 (-0.22, 0.01) |  |  | -0.10 (-0.23, 0.03) |  |  | 0.18 (0.01, 0.35) |  |  |
| NS | 0.23 (0.06, 0.40) |  |  | 0.00 (-0.17, 0.16) |  |  | -0.21 (-0.39, -0.04) |  |  | 0.12 (-0.10, 0.35) |  |  |
| MCS | 0.51 (0.34, 0.67) |  |  | 0.43 (0.27, 0.59) |  |  | -0.15 (-0.33, 0.03) |  |  | -0.17 (-0.40, 0.06) |  |  |
| Linear change * cohort (ref. NCDS) * birth sex (ref. Male) |  | 5.6 | 0.234 |  | 11.9 | 0.019 |  | 4.5 | 0.341 |  | 10.9 | 0.028 |
| NSHD | 0.19 (-0.07, 0.45) |  |  | 0.14 (-0.12, 0.39) |  |  | 0.14 (-0.18, 0.46) |  |  | -0.28 (-0.80, 0.24) |  |  |
| BCS | -0.02 (-0.23, 0.19) |  |  | -0.05 (-0.25, 0.14) |  |  | 0.15 (-0.07, 0.37) |  |  | -0.20 (-0.48, 0.09) |  |  |
| NS | -0.24 (-0.54, 0.06) |  |  | -0.38 (-0.67, -0.09) |  |  | -0.12 (-0.41, 0.17) |  |  | 0.39 (0.01, 0.76) |  |  |
| MCS | -0.02 (-0.34, 0.30) |  |  | -0.28 (-0.60, 0.04) |  |  | -0.09 (-0.42, 0.23) |  |  | 0.21 (-0.21, 0.63) |  |  |
| Quad. change * cohort (ref. NCDS) * birth sex (ref. Male) |  | 5.7 | 0.226 |  | 11.4 | 0.023 |  | 6.9 | 0.142 |  | 9.8 | 0.044 |
| NSHD | -0.10 (-0.23, 0.02) |  |  | -0.05 (-0.17, 0.07) |  |  | -0.05 (-0.21, 0.11) |  |  | 0.14 (-0.12, 0.41) |  |  |
| BCS | 0.02 (-0.08, 0.11) |  |  | 0.03 (-0.06, 0.13) |  |  | -0.09 (-0.20, 0.01) |  |  | 0.09 (-0.04, 0.23) |  |  |
| NS | 0.10 (-0.04, 0.23) |  |  | 0.19 (0.06, 0.32) |  |  | 0.07 (-0.06, 0.20) |  |  | -0.17 (-0.34, 0.00) |  |  |
| MCS | -0.01 (-0.16, 0.14) |  |  | 0.10 (-0.05, 0.24) |  |  | 0.03 (-0.12, 0.19) |  |  | -0.02 (-0.21, 0.17) |  |  |

*Note.* Adjusted models included highest qualification achieved; prospectively assessed pre-pandemic housing tenure, self-rated health, long-standing illness, psychological distress, and life satisfaction (life satisfaction only in models for that outcome); and household composition as covariates. Sensitivity models correspond to the unadjusted models after restricting the analytical sample to that of the adjusted models. BCS: British Cohort Study, 1970 birth cohort; GAD-2: 2-item General Anxiety Disorder questionnaire; MCS: Millennium Cohort Study, 2000 birth cohort; NCDS: National Child and Development Study, 1958 birth cohort; NS: Next Steps, 1990 cohort; NSHD: National Survey of Health and Development, 1946 birth cohort; ONS: UK Office for National Statistics; PHQ-2: 2-item Patient Health Questionnaire; UCLA-3: 3-item UCLA loneliness scale. χ2: Wald test performed to assess the overall statistical significance of the interaction terms; all χ2 statistics in this table have 4 degrees of freedom.

## Table S6.2. Unadjusted and adjusted marginal mean estimates and 95% confidence intervals by birth sex.

|  |  |  | **Anxiety symptomatology (GAD-2)** | **Depressive symptomatology (PHQ-2)** | **Feelings of loneliness (UCLA-3)** | **Life satisfaction (ONS single question)** |
| --- | --- | --- | --- | --- | --- | --- |
| **Cohort** | **Birth sex** | **Survey wave** | **Unadjusted marginal mean (95% CI)** | **Unadjusted marginal mean (95% CI)** | **Unadjusted marginal mean (95% CI)** | **Unadjusted marginal mean (95% CI)** |
| NSHD | Men | 1 | 0.28 (0.21, 0.34) | 0.31 (0.25, 0.37) | 3.75 (3.65, 3.85) | 7.75 (7.58, 7.92) |
| NSHD | Men | 2 | 0.40 (0.33, 0.47) | 0.33 (0.27, 0.39) | 3.61 (3.53, 3.70) | 7.66 (7.52, 7.80) |
| NSHD | Men | 3 | 0.37 (0.30, 0.44) | 0.38 (0.32, 0.45) | 3.87 (3.77, 3.97) | 7.22 (7.05, 7.38) |
| NSHD | Women | 1 | 0.69 (0.60, 0.78) | 0.59 (0.50, 0.68) | 4.19 (4.07, 4.32) | 7.39 (7.22, 7.56) |
| NSHD | Women | 2 | 0.85 (0.76, 0.95) | 0.65 (0.56, 0.73) | 4.05 (3.94, 4.16) | 7.25 (7.11, 7.39) |
| NSHD | Women | 3 | 0.81 (0.71, 0.90) | 0.70 (0.62, 0.79) | 4.39 (4.27, 4.51) | 6.74 (6.59, 6.89) |
| NCDS | Men | 1 | 0.50 (0.46, 0.55) | 0.52 (0.48, 0.57) | 3.94 (3.89, 4.00) | 7.58 (7.50, 7.65) |
| NCDS | Men | 2 | 0.53 (0.49, 0.57) | 0.55 (0.51, 0.59) | 3.86 (3.81, 3.91) | 7.52 (7.45, 7.59) |
| NCDS | Men | 3 | 0.52 (0.48, 0.56) | 0.65 (0.61, 0.69) | 4.07 (4.02, 4.12) | 7.16 (7.09, 7.22) |
| NCDS | Women | 1 | 0.97 (0.91, 1.02) | 0.80 (0.75, 0.85) | 4.33 (4.27, 4.39) | 7.36 (7.28, 7.44) |
| NCDS | Women | 2 | 1.03 (0.98, 1.08) | 0.80 (0.76, 0.85) | 4.21 (4.16, 4.27) | 7.30 (7.23, 7.36) |
| NCDS | Women | 3 | 1.01 (0.96, 1.06) | 0.94 (0.89, 0.98) | 4.53 (4.47, 4.58) | 6.78 (6.71, 6.85) |
| BCS | Men | 1 | 0.71 (0.65, 0.77) | 0.79 (0.73, 0.85) | 4.03 (3.96, 4.10) | 7.27 (7.18, 7.35) |
| BCS | Men | 2 | 0.79 (0.74, 0.85) | 0.81 (0.76, 0.87) | 4.09 (4.02, 4.15) | 7.16 (7.08, 7.24) |
| BCS | Men | 3 | 0.76 (0.71, 0.81) | 0.92 (0.86, 0.97) | 4.26 (4.20, 4.33) | 6.90 (6.82, 6.98) |
| BCS | Women | 1 | 1.12 (1.06, 1.17) | 0.98 (0.92, 1.03) | 4.37 (4.31, 4.43) | 7.17 (7.09, 7.24) |
| BCS | Women | 2 | 1.25 (1.19, 1.30) | 0.93 (0.88, 0.98) | 4.37 (4.31, 4.43) | 7.07 (7.00, 7.14) |
| BCS | Women | 3 | 1.21 (1.15, 1.26) | 1.11 (1.06, 1.17) | 4.54 (4.48, 4.60) | 6.68 (6.61, 6.75) |
| NS | Men | 1 | 1.05 (0.94, 1.17) | 1.24 (1.13, 1.36) | 4.54 (4.42, 4.66) | 6.90 (6.75, 7.04) |
| NS | Men | 2 | 1.25 (1.16, 1.34) | 1.26 (1.17, 1.35) | 4.61 (4.51, 4.70) | 6.83 (6.71, 6.94) |
| NS | Men | 3 | 1.24 (1.16, 1.32) | 1.31 (1.23, 1.39) | 4.66 (4.58, 4.75) | 6.64 (6.54, 6.75) |
| NS | Women | 1 | 1.74 (1.65, 1.83) | 1.53 (1.45, 1.62) | 4.71 (4.62, 4.79) | 6.77 (6.66, 6.89) |
| NS | Women | 2 | 1.83 (1.76, 1.91) | 1.32 (1.25, 1.39) | 4.68 (4.62, 4.75) | 6.97 (6.88, 7.05) |
| NS | Women | 3 | 1.86 (1.79, 1.93) | 1.60 (1.54, 1.67) | 4.94 (4.88, 5.01) | 6.46 (6.38, 6.55) |
| MCS | Men | 1 | 1.15 (1.04, 1.26) | 1.42 (1.31, 1.52) | 5.00 (4.88, 5.12) | 6.46 (6.31, 6.62) |
| MCS | Men | 2 | 1.47 (1.37, 1.57) | 1.40 (1.30, 1.49) | 5.07 (4.97, 5.18) | 6.47 (6.35, 6.60) |
| MCS | Men | 3 | 1.52 (1.44, 1.60) | 1.63 (1.55, 1.71) | 5.07 (4.99, 5.16) | 6.13 (6.02, 6.24) |
| MCS | Women | 1 | 2.17 (2.08, 2.26) | 2.19 (2.10, 2.27) | 5.26 (5.18, 5.35) | 5.99 (5.88, 6.10) |
| MCS | Women | 2 | 2.48 (2.39, 2.56) | 1.89 (1.81, 1.97) | 5.27 (5.19, 5.35) | 6.29 (6.19, 6.38) |
| MCS | Women | 3 | 2.51 (2.44, 2.59) | 2.24 (2.17, 2.31) | 5.39 (5.31, 5.46) | 5.81 (5.72, 5.90) |
| **Cohort** | **Birth sex** | **Survey wave** | **Adjusted marginal mean (95% CI)** | **Adjusted marginal mean (95% CI)** | **Adjusted marginal mean (95% CI)** | **Adjusted marginal mean (95% CI)** |
| NSHD | Men | 1 | 0.39 (0.32, 0.47) | 0.49 (0.42, 0.56) | 4.05 (3.94, 4.17) | 7.27 (7.08, 7.46) |
| NSHD | Men | 2 | 0.51 (0.43, 0.59) | 0.51 (0.44, 0.59) | 3.90 (3.80, 4.00) | 7.21 (7.04, 7.38) |
| NSHD | Men | 3 | 0.52 (0.43, 0.61) | 0.56 (0.48, 0.64) | 4.12 (4.01, 4.24) | 6.73 (6.51, 6.94) |
| NSHD | Women | 1 | 0.67 (0.57, 0.77) | 0.59 (0.49, 0.68) | 4.29 (4.16, 4.43) | 7.06 (6.88, 7.24) |
| NSHD | Women | 2 | 0.90 (0.79, 1.00) | 0.65 (0.56, 0.75) | 4.17 (4.04, 4.29) | 6.94 (6.76, 7.12) |
| NSHD | Women | 3 | 0.80 (0.69, 0.91) | 0.74 (0.64, 0.84) | 4.50 (4.36, 4.64) | 6.40 (6.20, 6.60) |
| NCDS | Men | 1 | 0.61 (0.56, 0.66) | 0.64 (0.60, 0.69) | 4.10 (4.04, 4.16) | 7.43 (7.35, 7.50) |
| NCDS | Men | 2 | 0.64 (0.60, 0.69) | 0.67 (0.62, 0.71) | 4.03 (3.98, 4.09) | 7.34 (7.27, 7.41) |
| NCDS | Men | 3 | 0.62 (0.58, 0.67) | 0.78 (0.73, 0.83) | 4.23 (4.17, 4.29) | 7.01 (6.94, 7.09) |
| NCDS | Women | 1 | 0.95 (0.90, 1.01) | 0.82 (0.77, 0.88) | 4.37 (4.31, 4.43) | 7.24 (7.16, 7.32) |
| NCDS | Women | 2 | 1.02 (0.97, 1.07) | 0.80 (0.76, 0.85) | 4.25 (4.19, 4.30) | 7.23 (7.16, 7.30) |
| NCDS | Women | 3 | 1.01 (0.96, 1.06) | 0.96 (0.91, 1.01) | 4.56 (4.51, 4.62) | 6.69 (6.62, 6.76) |
| BCS | Men | 1 | 0.73 (0.66, 0.80) | 0.85 (0.79, 0.91) | 4.17 (4.09, 4.25) | 7.14 (7.05, 7.23) |
| BCS | Men | 2 | 0.83 (0.77, 0.89) | 0.87 (0.81, 0.93) | 4.19 (4.12, 4.26) | 7.07 (6.99, 7.16) |
| BCS | Men | 3 | 0.75 (0.69, 0.80) | 0.94 (0.89, 1.00) | 4.35 (4.28, 4.42) | 6.84 (6.76, 6.92) |
| BCS | Women | 1 | 1.10 (1.04, 1.16) | 0.98 (0.93, 1.04) | 4.40 (4.34, 4.47) | 7.05 (6.97, 7.14) |
| BCS | Women | 2 | 1.22 (1.16, 1.28) | 0.92 (0.86, 0.97) | 4.40 (4.34, 4.47) | 6.97 (6.90, 7.05) |
| BCS | Women | 3 | 1.16 (1.10, 1.21) | 1.08 (1.03, 1.14) | 4.55 (4.49, 4.61) | 6.60 (6.52, 6.68) |
| NS | Men | 1 | 1.02 (0.91, 1.14) | 1.19 (1.08, 1.31) | 4.51 (4.39, 4.63) | 6.94 (6.79, 7.08) |
| NS | Men | 2 | 1.24 (1.14, 1.34) | 1.21 (1.12, 1.31) | 4.57 (4.47, 4.67) | 6.87 (6.75, 6.99) |
| NS | Men | 3 | 1.20 (1.11, 1.29) | 1.24 (1.15, 1.32) | 4.62 (4.53, 4.72) | 6.71 (6.60, 6.82) |
| NS | Women | 1 | 1.62 (1.52, 1.72) | 1.40 (1.31, 1.50) | 4.63 (4.54, 4.72) | 6.83 (6.70, 6.95) |
| NS | Women | 2 | 1.73 (1.65, 1.81) | 1.20 (1.12, 1.27) | 4.60 (4.52, 4.68) | 7.02 (6.93, 7.12) |
| NS | Women | 3 | 1.73 (1.65, 1.80) | 1.44 (1.37, 1.52) | 4.85 (4.77, 4.92) | 6.53 (6.44, 6.63) |
| MCS | Men | 1 | 1.30 (1.17, 1.42) | 1.53 (1.41, 1.65) | 4.84 (4.70, 4.98) | 6.76 (6.59, 6.93) |
| MCS | Men | 2 | 1.61 (1.49, 1.72) | 1.50 (1.39, 1.61) | 4.96 (4.83, 5.08) | 6.77 (6.63, 6.92) |
| MCS | Men | 3 | 1.68 (1.58, 1.78) | 1.76 (1.67, 1.86) | 4.95 (4.84, 5.05) | 6.39 (6.26, 6.52) |
| MCS | Women | 1 | 2.10 (2.00, 2.20) | 2.09 (2.00, 2.19) | 4.96 (4.86, 5.05) | 6.61 (6.49, 6.74) |
| MCS | Women | 2 | 2.43 (2.33, 2.53) | 1.85 (1.76, 1.95) | 4.97 (4.87, 5.07) | 6.87 (6.75, 6.99) |
| MCS | Women | 3 | 2.45 (2.36, 2.54) | 2.16 (2.07, 2.24) | 5.10 (5.01, 5.19) | 6.43 (6.32, 6.54) |

*Note.* Adjusted models included highest qualification achieved; prospectively assessed pre-pandemic housing tenure, self-rated health, long-standing illness, psychological distress, and life satisfaction (life satisfaction only in models for that outcome); and household composition as covariates. BCS: British Cohort Study, 1970 birth cohort; GAD-2: 2-item General Anxiety Disorder questionnaire; MCS: Millennium Cohort Study, 2000 birth cohort; NCDS: National Child and Development Study, 1958 birth cohort; NS: Next Steps, 1990 cohort; NSHD: National Survey of Health and Development, 1946 birth cohort; ONS: UK Office for National Statistics; PHQ-2: 2-item Patient Health Questionnaire; UCLA-3: 3-item UCLA loneliness scale. Survey wave 1: May 2020; survey wave 2: September/October 2020; survey wave 3: February/March 2021.

## Table S6.3. Difference-in-differences (change in difference between men and women between first vs last time point) estimates and 95% confidence intervals.

|  | *DID* (95% CI) | *p* |
| --- | --- | --- |
| GAD-2 | -0.003 (-0.062, 0.056) | 0.925 |
| PHQ-2 | -0.016 (-0.073, 0.041) | 0.578 |
| UCLA-3 | 0.061 (-0.004, 0.125) | 0.064 |
| Life satisfaction | -0.060 (-0.155, 0.034) | 0.048 |

*Note*. DID: difference-in-differences; GAD-2: 2-item General Anxiety Disorder questionnaire; PHQ-2: 2-item Patient Health Questionnaire; UCLA-3: 3-item UCLA loneliness scale.

## Figure S6.1. Unadjusted and adjusted anxiety symptomatology (GAD-2) marginal mean estimates and 95% confidence intervals by birth sex.

Adjusted models include highest qualification achieved and pre-pandemic housing tenure, pre-pandemic self-reported health and long-standing illness, pre-pandemic psychological distress, and household composition.

**
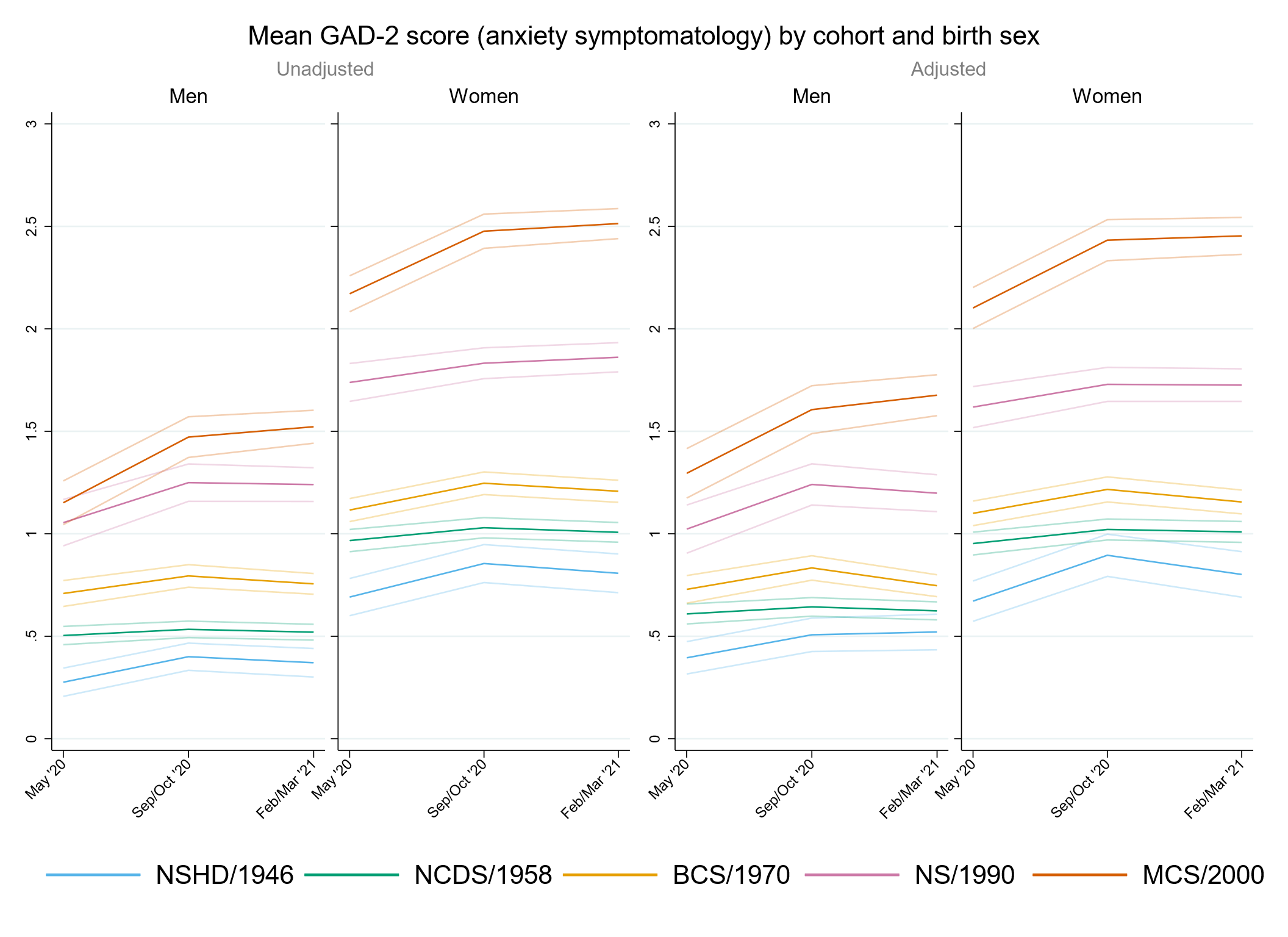
**

## Figure S6.2. Unadjusted and adjusted depressive symptomatology (PHQ-2) marginal mean estimates and 95% confidence intervals by birth sex.

Adjusted models include highest qualification achieved and pre-pandemic housing tenure, pre-pandemic self-reported health and long-standing illness, pre-pandemic psychological distress, and household composition.

**
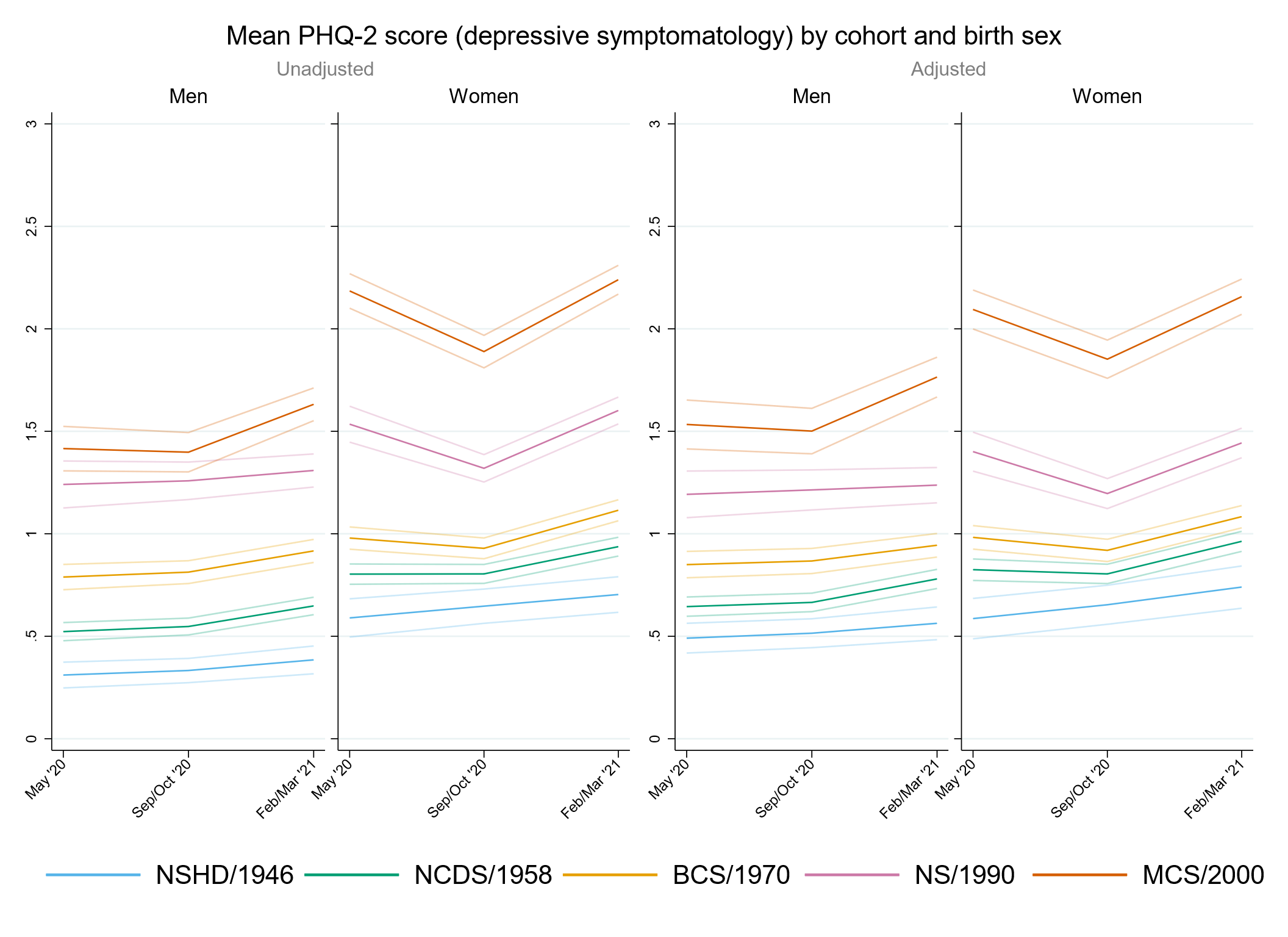
**

## Figure S6.3. Unadjusted and adjusted loneliness (UCLA-3) marginal mean estimates and 95% confidence intervals by birth sex.

Adjusted models include highest qualification achieved and pre-pandemic housing tenure, pre-pandemic self-reported health and long-standing illness, pre-pandemic psychological distress, and household composition.

**
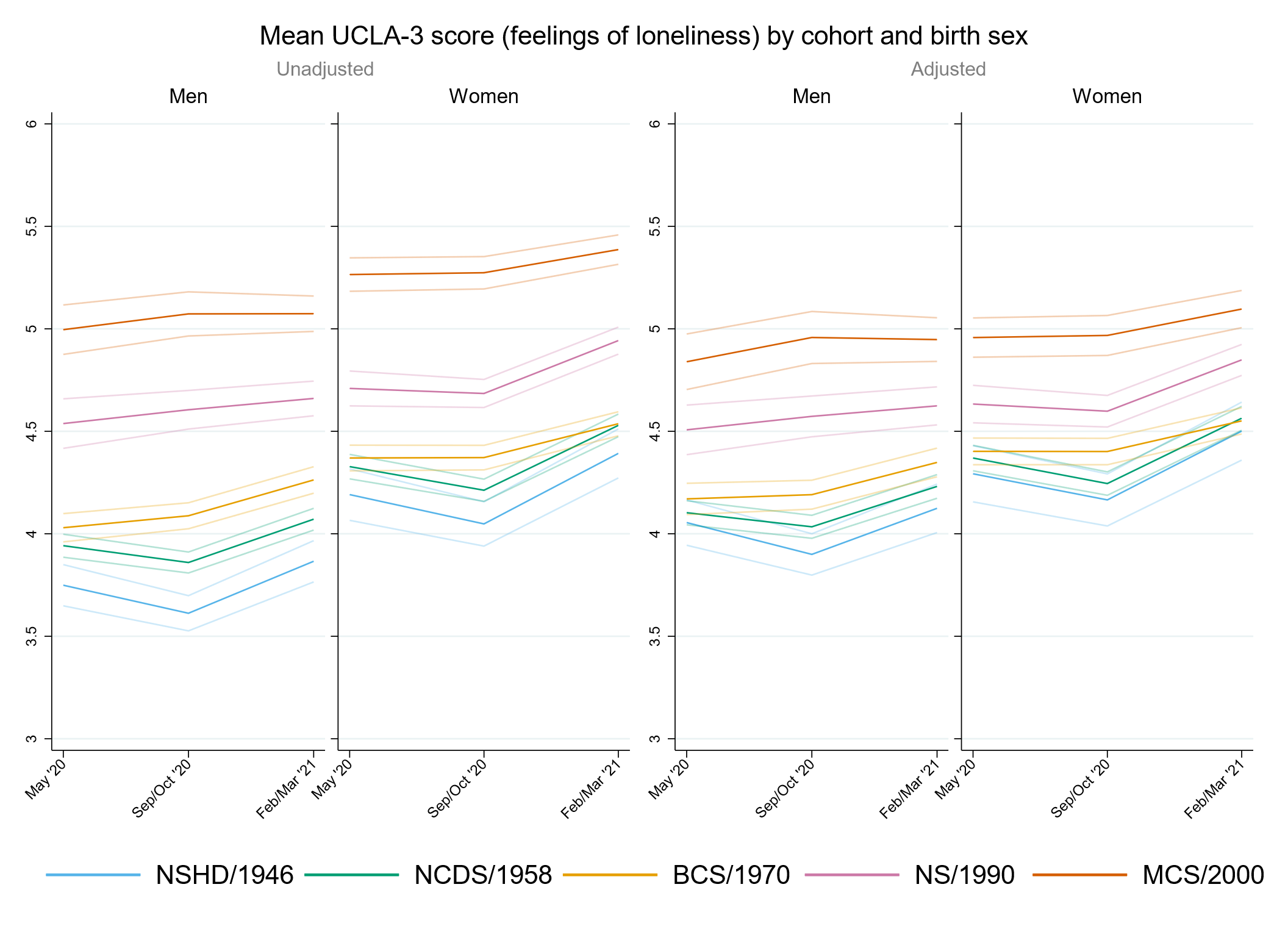
**

## Figure S6.4. Unadjusted and adjusted life satisfaction marginal mean estimates and 95% confidence intervals by birth sex.

Adjusted models include highest qualification achieved and pre-pandemic housing tenure, pre-pandemic self-reported health and long-standing illness, pre-pandemic life satisfaction and psychological distress, and household composition.
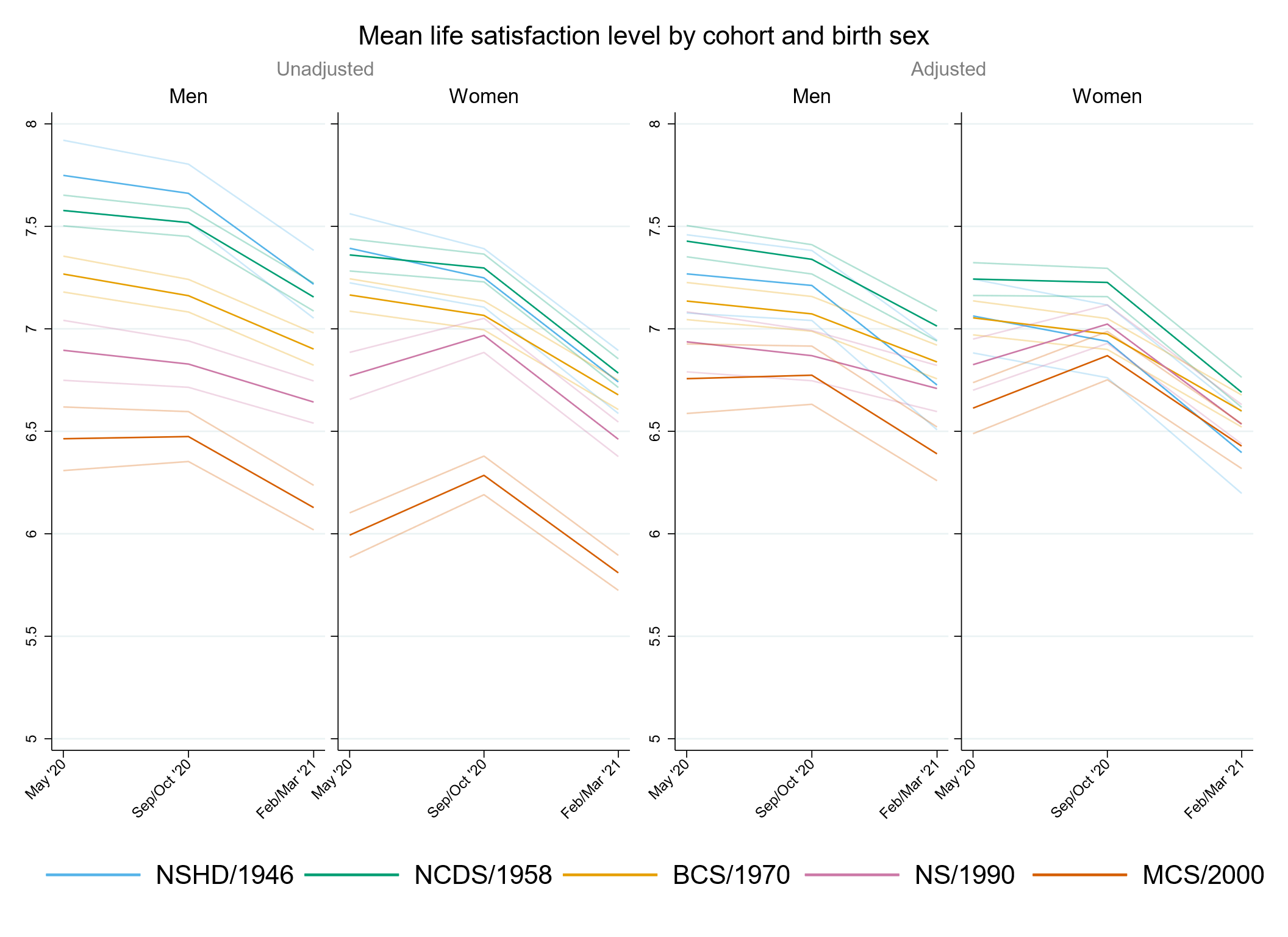


# Appendix S7. Results by socioeconomic position using retrospectively assessed pre-pandemic financial situation as indicator.

## Table S7.1. Results of multilevel growth curve models by pre-pandemic financial situation.

|  | **Anxiety symptomatology (GAD-2)** | | | **Depressive symptomatology (PHQ-2)** | | | **Feelings of loneliness (UCLA-3)** | | | **Life satisfaction (ONS single question)** | | |
| --- | --- | --- | --- | --- | --- | --- | --- | --- | --- | --- | --- | --- |
| **Unadjusted models** |  | | |  | | |  | | |  | | |
| N participants | 24,874 |  |  | 24,873 |  |  | 24,913 |  |  | 24,963 |  |  |
| N observations | 52,962 |  |  | 52,947 |  |  | 53,039 |  |  | 53,262 |  |  |
|  | ***B* (95% CI)** | **χ2** | ***p*** | ***B* (95% CI)** | **χ2** | ***p*** | ***B* (95% CI)** | **χ2** | ***p*** | ***B* (95% CI)** | **χ2** | ***p*** |
| Time (linear) | 0.09 (-0.02, 0.20) |  | 0.113 | 0.05 (-0.05, 0.15) |  | 0.310 | -0.27 (-0.39, -0.15) |  | <0.001 | -0.01 (-0.16, 0.14) |  | 0.890 |
| Time (quad) | -0.04 (-0.09, 0.02) |  | 0.167 | 0.02 (-0.03, 0.06) |  | 0.445 | 0.18 (0.13, 0.24) |  | <0.001 | -0.11 (-0.18, -0.04) |  | 0.003 |
| Cohort (ref. NCDS) |  | 375.9 | <0.001 |  | 518.6 | <0.001 |  | 319.7 | <0.001 |  | 268.0 | <0.001 |
| NSHD | -0.20 (-0.34, -0.05) |  |  | -0.06 (-0.21, 0.09) |  |  | -0.08 (-0.28, 0.12) |  |  | 0.03 (-0.24, 0.30) |  |  |
| BCS | 0.11 (0.01, 0.20) |  |  | 0.17 (0.09, 0.26) |  |  | -0.04 (-0.15, 0.06) |  |  | -0.11 (-0.24, 0.01) |  |  |
| NS | 0.65 (0.52, 0.78) |  |  | 0.69 (0.56, 0.81) |  |  | 0.40 (0.27, 0.54) |  |  | -0.55 (-0.71, -0.39) |  |  |
| MCS | 0.98 (0.86, 1.10) |  |  | 1.20 (1.09, 1.32) |  |  | 0.96 (0.83, 1.08) |  |  | -1.20 (-1.36, -1.04) |  |  |
| Linear change * cohort (ref. NCDS) |  | 6.7 | 0.150 |  | 36.9 | <0.001 |  | 12.3 | 0.015 |  | 31.7 | <0.001 |
| NSHD | 0.20 (-0.04, 0.43) |  |  | -0.08 (-0.31, 0.15) |  |  | -0.12 (-0.45, 0.22) |  |  | -0.05 (-0.49, 0.38) |  |  |
| BCS | 0.04 (-0.12, 0.19) |  |  | -0.21 (-0.35, -0.06) |  |  | 0.25 (0.08, 0.42) |  |  | -0.01 (-0.22, 0.21) |  |  |
| NS | 0.09 (-0.14, 0.31) |  |  | -0.40 (-0.61, -0.20) |  |  | 0.16 (-0.06, 0.38) |  |  | 0.44 (0.17, 0.70) |  |  |
| MCS | 0.26 (0.03, 0.48) |  |  | -0.60 (-0.82, -0.38) |  |  | 0.24 (0.01, 0.47) |  |  | 0.64 (0.36, 0.93) |  |  |
| Quad. change * cohort (ref. NCDS) |  | 1.7 | 0.792 |  | 35.1 | <0.001 |  | 17.3 | 0.002 |  | 24.6 | <0.001 |
| NSHD | -0.06 (-0.18, 0.05) |  |  | 0.01 (-0.10, 0.13) |  |  | 0.08 (-0.08, 0.23) |  |  | 0.00 (-0.21, 0.20) |  |  |
| BCS | -0.01 (-0.08, 0.06) |  |  | 0.08 (0.01, 0.15) |  |  | -0.12 (-0.20, -0.05) |  |  | 0.01 (-0.09, 0.11) |  |  |
| NS | -0.02 (-0.12, 0.08) |  |  | 0.16 (0.07, 0.26) |  |  | -0.09 (-0.19, 0.00) |  |  | -0.14 (-0.26, -0.03) |  |  |
| MCS | -0.05 (-0.15, 0.06) |  |  | 0.28 (0.17, 0.38) |  |  | -0.15 (-0.25, -0.04) |  |  | -0.27 (-0.40, -0.14) |  |  |
| Pre-pandemic financial situation (ref. Doing all right) |  | 132.0 | <0.001 |  | 217.1 | <0.001 |  | 252.4 | <0.001 |  | 322.6 | <0.001 |
| Experiencing difficulties | 0.61 (0.45, 0.78) |  |  | 0.87 (0.70, 1.04) |  |  | 0.95 (0.77, 1.14) |  |  | -1.40 (-1.64, -1.16) |  |  |
| Living comfortably | -0.24 (-0.32, -0.16) |  |  | -0.26 (-0.33, -0.19) |  |  | -0.37 (-0.46, -0.28) |  |  | 0.54 (0.43, 0.66) |  |  |
| Linear change * pre-pandemic financial situation (ref. Doing all right) |  | 4.0 | 0.134 |  | 8.7 | 0.013 |  | 0.5 | 0.771 |  | 7.6 | 0.022 |
| Experiencing difficulties | 0.18 (-0.09, 0.44) |  |  | -0.03 (-0.30, 0.23) |  |  | -0.10 (-0.38, 0.18) |  |  | 0.11 (-0.25, 0.48) |  |  |
| Living comfortably | -0.06 (-0.19, 0.07) |  |  | -0.17 (-0.28, -0.05) |  |  | -0.02 (-0.17, 0.12) |  |  | 0.26 (0.07, 0.44) |  |  |
| Quad. change * pre-pandemic financial situation (ref. Doing all right) |  | 6.0 | 0.051 |  | 7.0 | 0.030 |  | 0.2 | 0.930 |  | 15.7 | <0.001 |
| Experiencing difficulties | -0.11 (-0.23, 0.01) |  |  | -0.01 (-0.13, 0.12) |  |  | 0.02 (-0.10, 0.15) |  |  | 0.01 (-0.16, 0.18) |  |  |
| Living comfortably | 0.03 (-0.03, 0.09) |  |  | 0.07 (0.01, 0.12) |  |  | 0.00 (-0.06, 0.07) |  |  | -0.16 (-0.25, -0.07) |  |  |
| Cohort (ref. NCDS) * pre-pandemic financial situation (ref. Doing all right) |  | 4.5 | 0.812 |  | 5.6 | 0.694 |  | 21.2 | 0.007 |  | 13.0 | 0.113 |
| NSHD * Experiencing difficulties | -0.13 (-0.62, 0.36) |  |  | -0.20 (-0.79, 0.39) |  |  | -0.50 (-1.06, 0.06) |  |  | 0.32 (-0.52, 1.15) |  |  |
| NSHD * Living comfortably | 0.05 (-0.11, 0.21) |  |  | -0.04 (-0.21, 0.12) |  |  | 0.06 (-0.16, 0.28) |  |  | -0.18 (-0.49, 0.13) |  |  |
| BCS * Experiencing difficulties | 0.07 (-0.16, 0.30) |  |  | -0.10 (-0.34, 0.13) |  |  | -0.01 (-0.26, 0.24) |  |  | 0.18 (-0.13, 0.50) |  |  |
| BCS * Living comfortably | 0.01 (-0.10, 0.13) |  |  | -0.04 (-0.14, 0.07) |  |  | 0.03 (-0.09, 0.16) |  |  | -0.03 (-0.19, 0.13) |  |  |
| NS * Experiencing difficulties | 0.09 (-0.21, 0.38) |  |  | -0.15 (-0.44, 0.13) |  |  | -0.19 (-0.49, 0.10) |  |  | 0.43 (0.05, 0.81) |  |  |
| NS * Living comfortably | -0.05 (-0.22, 0.12) |  |  | -0.05 (-0.21, 0.11) |  |  | -0.02 (-0.19, 0.15) |  |  | 0.07 (-0.15, 0.28) |  |  |
| MCS * Experiencing difficulties | -0.09 (-0.37, 0.18) |  |  | -0.29 (-0.55, -0.02) |  |  | -0.50 (-0.77, -0.22) |  |  | 0.53 (0.18, 0.89) |  |  |
| MCS * Living comfortably | -0.09 (-0.26, 0.08) |  |  | -0.12 (-0.28, 0.04) |  |  | 0.00 (-0.17, 0.17) |  |  | 0.09 (-0.13, 0.31) |  |  |
| Linear change * cohort (ref. NCDS) * pre-pandemic financial situation (ref. Doing all right) |  | 5.9 | 0.657 |  | 10.3 | 0.242 |  | 6.1 | 0.634 |  | 11.3 | 0.184 |
| NSHD * Experiencing difficulties | -0.31 (-1.04, 0.43) |  |  | -0.11 (-1.12, 0.89) |  |  | 0.41 (-0.21, 1.04) |  |  | 0.85 (-0.36, 2.06) |  |  |
| NSHD * Living comfortably | 0.00 (-0.27, 0.28) |  |  | 0.27 (0.00, 0.53) |  |  | 0.04 (-0.33, 0.41) |  |  | -0.13 (-0.64, 0.38) |  |  |
| BCS * Experiencing difficulties | 0.16 (-0.24, 0.57) |  |  | 0.26 (-0.14, 0.66) |  |  | 0.03 (-0.37, 0.44) |  |  | -0.30 (-0.81, 0.21) |  |  |
| BCS * Living comfortably | 0.05 (-0.15, 0.24) |  |  | 0.20 (0.02, 0.37) |  |  | -0.02 (-0.23, 0.19) |  |  | -0.09 (-0.36, 0.18) |  |  |
| NS * Experiencing difficulties | -0.16 (-0.65, 0.33) |  |  | 0.39 (-0.08, 0.87) |  |  | 0.36 (-0.12, 0.84) |  |  | -0.51 (-1.12, 0.11) |  |  |
| NS * Living comfortably | 0.04 (-0.26, 0.33) |  |  | 0.12 (-0.16, 0.39) |  |  | -0.08 (-0.36, 0.20) |  |  | -0.22 (-0.57, 0.14) |  |  |
| MCS * Experiencing difficulties | -0.17 (-0.66, 0.31) |  |  | 0.36 (-0.12, 0.84) |  |  | 0.10 (-0.38, 0.58) |  |  | -0.35 (-0.97, 0.26) |  |  |
| MCS * Living comfortably | 0.22 (-0.10, 0.54) |  |  | 0.24 (-0.07, 0.55) |  |  | 0.13 (-0.18, 0.45) |  |  | -0.47 (-0.87, -0.06) |  |  |
| Quad. change * cohort (ref. NCDS) * pre-pandemic financial situation (ref. Doing all right) |  | 6.6 | 0.582 |  | 7.9 | 0.442 |  | 9.2 | 0.324 |  | 12.3 | 0.138 |
| NSHD * Experiencing difficulties | 0.13 (-0.21, 0.47) |  |  | 0.01 (-0.45, 0.48) |  |  | -0.22 (-0.55, 0.11) |  |  | -0.53 (-1.24, 0.19) |  |  |
| NSHD * Living comfortably | -0.02 (-0.15, 0.12) |  |  | -0.10 (-0.23, 0.03) |  |  | -0.05 (-0.22, 0.13) |  |  | 0.09 (-0.15, 0.33) |  |  |
| BCS * Experiencing difficulties | -0.05 (-0.24, 0.13) |  |  | -0.07 (-0.26, 0.11) |  |  | -0.02 (-0.21, 0.17) |  |  | 0.14 (-0.09, 0.37) |  |  |
| BCS * Living comfortably | -0.02 (-0.11, 0.07) |  |  | -0.07 (-0.15, 0.01) |  |  | 0.03 (-0.07, 0.12) |  |  | 0.06 (-0.07, 0.19) |  |  |
| NS * Experiencing difficulties | 0.08 (-0.14, 0.30) |  |  | -0.17 (-0.38, 0.04) |  |  | -0.17 (-0.38, 0.04) |  |  | 0.18 (-0.09, 0.45) |  |  |
| NS * Living comfortably | -0.01 (-0.15, 0.12) |  |  | -0.03 (-0.15, 0.10) |  |  | 0.07 (-0.05, 0.20) |  |  | 0.07 (-0.09, 0.23) |  |  |
| MCS * Experiencing difficulties | 0.08 (-0.14, 0.31) |  |  | -0.17 (-0.40, 0.05) |  |  | -0.01 (-0.24, 0.21) |  |  | 0.18 (-0.11, 0.46) |  |  |
| MCS * Living comfortably | -0.13 (-0.27, 0.02) |  |  | -0.10 (-0.25, 0.04) |  |  | -0.06 (-0.21, 0.09) |  |  | 0.25 (0.07, 0.44) |  |  |
| **Adjusted models** |  | | |  | | |  | | |  | | |
| N participants | 21,581 |  |  | 21,580 |  |  | 21,610 |  |  | 21,431 |  |  |
| N observations | 46,915 |  |  | 46,905 |  |  | 46,983 |  |  | 46,621 |  |  |
|  | ***B* (95% CI)** | **χ2** | ***p*** | ***B* (95% CI)** | **χ2** | ***p*** | ***B* (95% CI)** | **χ2** | ***p*** | ***B* (95% CI)** | **χ2** | ***p*** |
| Time (linear) | 0.11 (-0.01, 0.22) |  | 0.077 | 0.03 (-0.07, 0.14) |  | 0.542 | -0.24 (-0.37, -0.12) |  | 0.000 | -0.05 (-0.21, 0.11) |  | 0.547 |
| Time (quad) | -0.04 (-0.10, 0.01) |  | 0.126 | 0.03 (-0.02, 0.08) |  | 0.263 | 0.17 (0.11, 0.23) |  | 0.000 | -0.09 (-0.16, -0.01) |  | 0.027 |
| Cohort (ref. NCDS) |  | 306.8 | <0.001 |  | 426.3 | <0.001 |  | 126.7 | <0.001 |  | 85.7 | <0.001 |
| NSHD | -0.28 (-0.42, -0.14) |  |  | -0.10 (-0.25, 0.05) |  |  | -0.12 (-0.32, 0.09) |  |  | 0.00 (-0.31, 0.31) |  |  |
| BCS | 0.12 (0.02, 0.21) |  |  | 0.21 (0.12, 0.29) |  |  | 0.03 (-0.08, 0.14) |  |  | -0.23 (-0.37, -0.10) |  |  |
| NS | 0.54 (0.41, 0.67) |  |  | 0.65 (0.52, 0.78) |  |  | 0.36 (0.23, 0.50) |  |  | -0.50 (-0.66, -0.33) |  |  |
| MCS | 0.97 (0.84, 1.10) |  |  | 1.20 (1.07, 1.32) |  |  | 0.68 (0.54, 0.82) |  |  | -0.75 (-0.92, -0.58) |  |  |
| Linear change * cohort (ref. NCDS) |  | 9.7 | 0.047 |  | 25.7 | <0.001 |  | 8.6 | 0.072 |  | 23.3 | <0.001 |
| NSHD | 0.23 (-0.03, 0.48) |  |  | -0.04 (-0.29, 0.21) |  |  | -0.09 (-0.44, 0.25) |  |  | 0.00 (-0.53, 0.53) |  |  |
| BCS | 0.01 (-0.16, 0.17) |  |  | -0.22 (-0.38, -0.07) |  |  | 0.20 (0.01, 0.38) |  |  | 0.04 (-0.19, 0.28) |  |  |
| NS | 0.15 (-0.09, 0.39) |  |  | -0.33 (-0.55, -0.11) |  |  | 0.14 (-0.10, 0.37) |  |  | 0.42 (0.13, 0.71) |  |  |
| MCS | 0.30 (0.06, 0.53) |  |  | -0.51 (-0.74, -0.28) |  |  | 0.28 (0.04, 0.52) |  |  | 0.61 (0.31, 0.91) |  |  |
| Quad. change * cohort (ref. NCDS) |  | 2.7 | 0.611 |  | 24.8 | <0.001 |  | 12.6 | 0.014 |  | 20.2 | <0.001 |
| NSHD | -0.08 (-0.21, 0.05) |  |  | -0.01 (-0.13, 0.10) |  |  | 0.06 (-0.10, 0.23) |  |  | -0.02 (-0.27, 0.24) |  |  |
| BCS | -0.01 (-0.09, 0.07) |  |  | 0.08 (0.01, 0.15) |  |  | -0.11 (-0.19, -0.02) |  |  | -0.01 (-0.12, 0.10) |  |  |
| NS | -0.05 (-0.16, 0.06) |  |  | 0.12 (0.02, 0.22) |  |  | -0.08 (-0.19, 0.02) |  |  | -0.13 (-0.27, 0.00) |  |  |
| MCS | -0.06 (-0.17, 0.05) |  |  | 0.24 (0.14, 0.35) |  |  | -0.15 (-0.26, -0.04) |  |  | -0.27 (-0.41, -0.14) |  |  |
| Pre-pandemic financial situation (ref. Doing all right) |  | 37.8 | <0.001 |  | 69.0 | <0.001 |  | 88.2 | <0.001 |  | 97.8 | <0.001 |
| Experiencing difficulties | 0.32 (0.15, 0.50) |  |  | 0.56 (0.38, 0.73) |  |  | 0.64 (0.44, 0.83) |  |  | -0.97 (-1.22, -0.72) |  |  |
| Living comfortably | -0.14 (-0.21, -0.06) |  |  | -0.13 (-0.20, -0.06) |  |  | -0.20 (-0.29, -0.11) |  |  | 0.22 (0.10, 0.33) |  |  |
| Linear change * pre-pandemic financial situation (ref. Doing all right) |  | 3.9 | 0.141 |  | 7.2 | 0.028 |  | 1.3 | 0.534 |  | 9.6 | 0.008 |
| Experiencing difficulties | 0.19 (-0.11, 0.48) |  |  | -0.12 (-0.41, 0.18) |  |  | -0.17 (-0.46, 0.13) |  |  | 0.24 (-0.17, 0.65) |  |  |
| Living comfortably | -0.07 (-0.21, 0.06) |  |  | -0.17 (-0.29, -0.04) |  |  | -0.04 (-0.19, 0.11) |  |  | 0.31 (0.11, 0.50) |  |  |
| Quad. change * pre-pandemic financial situation (ref. Doing all right) |  | 5.6 | 0.061 |  | 5.5 | 0.063 |  | 0.4 | 0.804 |  | 16.2 | <0.001 |
| Experiencing difficulties | -0.11 (-0.24, 0.03) |  |  | 0.05 (-0.09, 0.18) |  |  | 0.05 (-0.09, 0.18) |  |  | -0.06 (-0.25, 0.14) |  |  |
| Living comfortably | 0.03 (-0.03, 0.10) |  |  | 0.07 (0.01, 0.13) |  |  | 0.01 (-0.06, 0.08) |  |  | -0.18 (-0.28, -0.09) |  |  |
| Cohort (ref. NCDS) * pre-pandemic financial situation (ref. Doing all right) |  | 11.8 | 0.162 |  | 8.3 | 0.401 |  | 5.6 | 0.695 |  | 10.5 | 0.233 |
| NSHD * Experiencing difficulties | 0.22 (-0.33, 0.78) |  |  | 0.07 (-0.55, 0.70) |  |  | -0.24 (-0.84, 0.36) |  |  | 0.17 (-0.71, 1.05) |  |  |
| NSHD * Living comfortably | 0.08 (-0.07, 0.24) |  |  | -0.07 (-0.23, 0.10) |  |  | 0.08 (-0.15, 0.31) |  |  | -0.21 (-0.56, 0.13) |  |  |
| BCS * Experiencing difficulties | -0.08 (-0.32, 0.16) |  |  | -0.20 (-0.44, 0.04) |  |  | -0.16 (-0.42, 0.10) |  |  | 0.32 (0.00, 0.65) |  |  |
| BCS * Living comfortably | 0.03 (-0.08, 0.14) |  |  | -0.02 (-0.13, 0.09) |  |  | 0.02 (-0.11, 0.14) |  |  | 0.03 (-0.14, 0.19) |  |  |
| NS * Experiencing difficulties | 0.26 (-0.04, 0.57) |  |  | 0.01 (-0.29, 0.30) |  |  | -0.09 (-0.39, 0.21) |  |  | 0.22 (-0.17, 0.61) |  |  |
| NS * Living comfortably | -0.02 (-0.19, 0.15) |  |  | -0.08 (-0.24, 0.08) |  |  | -0.04 (-0.21, 0.13) |  |  | 0.08 (-0.14, 0.29) |  |  |
| MCS * Experiencing difficulties | 0.10 (-0.18, 0.37) |  |  | -0.10 (-0.37, 0.16) |  |  | -0.24 (-0.51, 0.04) |  |  | 0.23 (-0.13, 0.59) |  |  |
| MCS * Living comfortably | -0.10 (-0.26, 0.06) |  |  | -0.14 (-0.30, 0.01) |  |  | -0.09 (-0.26, 0.08) |  |  | 0.21 (0.00, 0.43) |  |  |
| Linear change * cohort (ref. NCDS) * pre-pandemic financial situation (ref. Doing all right) |  | 8.2 | 0.413 |  | 9.1 | 0.331 |  | 8.7 | 0.367 |  | 10.9 | 0.210 |
| NSHD * Experiencing difficulties | -0.37 (-1.24, 0.49) |  |  | -0.10 (-1.18, 0.98) |  |  | 0.25 (-0.47, 0.97) |  |  | 1.66 (-0.47, 3.79) |  |  |
| NSHD * Living comfortably | -0.03 (-0.33, 0.26) |  |  | 0.22 (-0.07, 0.51) |  |  | 0.01 (-0.38, 0.40) |  |  | -0.17 (-0.77, 0.44) |  |  |
| BCS * Experiencing difficulties | 0.23 (-0.23, 0.69) |  |  | 0.38 (-0.07, 0.83) |  |  | 0.16 (-0.28, 0.60) |  |  | -0.37 (-0.94, 0.20) |  |  |
| BCS * Living comfortably | 0.07 (-0.14, 0.28) |  |  | 0.21 (0.02, 0.41) |  |  | 0.02 (-0.20, 0.25) |  |  | -0.15 (-0.45, 0.15) |  |  |
| NS * Experiencing difficulties | -0.26 (-0.79, 0.27) |  |  | 0.34 (-0.17, 0.85) |  |  | 0.50 (0.00, 1.00) |  |  | -0.46 (-1.12, 0.21) |  |  |
| NS * Living comfortably | -0.02 (-0.33, 0.29) |  |  | 0.11 (-0.19, 0.40) |  |  | -0.11 (-0.40, 0.19) |  |  | -0.22 (-0.60, 0.17) |  |  |
| MCS * Experiencing difficulties | -0.27 (-0.79, 0.25) |  |  | 0.43 (-0.08, 0.94) |  |  | 0.04 (-0.47, 0.54) |  |  | -0.35 (-1.01, 0.31) |  |  |
| MCS * Living comfortably | 0.21 (-0.11, 0.54) |  |  | 0.23 (-0.10, 0.55) |  |  | 0.17 (-0.17, 0.51) |  |  | -0.51 (-0.93, -0.09) |  |  |
| Quad. change * cohort (ref. NCDS) * pre-pandemic financial situation (ref. Doing all right) |  | 8.5 | 0.384 |  | 7.8 | 0.450 |  | 14.2 | 0.078 |  | 12.7 | 0.121 |
| NSHD * Experiencing difficulties | 0.13 (-0.28, 0.54) |  |  | 0.00 (-0.53, 0.52) |  |  | -0.18 (-0.58, 0.22) |  |  | -1.07 (-2.49, 0.35) |  |  |
| NSHD * Living comfortably | 0.00 (-0.15, 0.15) |  |  | -0.07 (-0.21, 0.07) |  |  | -0.04 (-0.22, 0.15) |  |  | 0.09 (-0.20, 0.38) |  |  |
| BCS * Experiencing difficulties | -0.08 (-0.29, 0.13) |  |  | -0.15 (-0.36, 0.06) |  |  | -0.06 (-0.26, 0.14) |  |  | 0.17 (-0.09, 0.43) |  |  |
| BCS * Living comfortably | -0.03 (-0.13, 0.06) |  |  | -0.08 (-0.17, 0.01) |  |  | 0.00 (-0.10, 0.11) |  |  | 0.08 (-0.06, 0.22) |  |  |
| NS * Experiencing difficulties | 0.11 (-0.12, 0.35) |  |  | -0.16 (-0.39, 0.07) |  |  | -0.23 (-0.45, -0.01) |  |  | 0.18 (-0.12, 0.48) |  |  |
| NS * Living comfortably | 0.01 (-0.13, 0.15) |  |  | -0.01 (-0.14, 0.12) |  |  | 0.09 (-0.04, 0.22) |  |  | 0.06 (-0.11, 0.23) |  |  |
| MCS * Experiencing difficulties | 0.12 (-0.12, 0.36) |  |  | -0.22 (-0.46, 0.02) |  |  | 0.02 (-0.22, 0.25) |  |  | 0.19 (-0.12, 0.49) |  |  |
| MCS * Living comfortably | -0.13 (-0.29, 0.02) |  |  | -0.11 (-0.26, 0.04) |  |  | -0.10 (-0.26, 0.06) |  |  | 0.28 (0.09, 0.48) |  |  |
| **Sensitivity models** |  | | |  | | |  | | |  | | |
| N participants | 21,581 |  |  | 21,580 |  |  | 21,610 |  |  | 21,431 |  |  |
| N observations | 46,915 |  |  | 46,905 |  |  | 46,983 |  |  | 46,621 |  |  |
|  | ***B* (95% CI)** | **χ2** | ***p*** | ***B* (95% CI)** | **χ2** | ***p*** | ***B* (95% CI)** | **χ2** | ***p*** | ***B* (95% CI)** | **χ2** | ***p*** |
| Time (linear) | 0.11 (-0.01, 0.23) |  | 0.064 | 0.04 (-0.07, 0.14) |  | 0.497 | -0.24 (-0.37, -0.11) |  | 0.000 | -0.06 (-0.22, 0.10) |  | 0.444 |
| Time (quad) | -0.04 (-0.10, 0.01) |  | 0.112 | 0.03 (-0.02, 0.08) |  | 0.290 | 0.17 (0.11, 0.23) |  | 0.000 | -0.08 (-0.16, -0.01) |  | 0.032 |
| Cohort (ref. NCDS) |  | 353.8 | <0.001 |  | 470.8 | <0.001 |  | 285.4 | <0.001 |  | 232.5 | <0.001 |
| NSHD | -0.22 (-0.36, -0.08) |  |  | -0.06 (-0.22, 0.09) |  |  | -0.10 (-0.30, 0.11) |  |  | 0.01 (-0.30, 0.33) |  |  |
| BCS | 0.14 (0.04, 0.23) |  |  | 0.19 (0.10, 0.28) |  |  | -0.03 (-0.14, 0.08) |  |  | -0.15 (-0.28, -0.01) |  |  |
| NS | 0.65 (0.51, 0.78) |  |  | 0.70 (0.57, 0.83) |  |  | 0.41 (0.27, 0.55) |  |  | -0.54 (-0.72, -0.37) |  |  |
| MCS | 0.98 (0.86, 1.10) |  |  | 1.18 (1.06, 1.30) |  |  | 0.94 (0.82, 1.07) |  |  | -1.18 (-1.34, -1.01) |  |  |
| Linear change * cohort (ref. NCDS) |  | 8.3 | 0.083 |  | 27.7 | <0.001 |  | 7.8 | 0.098 |  | 25.5 | <0.001 |
| NSHD | 0.23 (-0.03, 0.49) |  |  | -0.04 (-0.29, 0.22) |  |  | -0.09 (-0.43, 0.26) |  |  | 0.02 (-0.51, 0.55) |  |  |
| BCS | 0.00 (-0.17, 0.17) |  |  | -0.23 (-0.39, -0.08) |  |  | 0.19 (0.01, 0.38) |  |  | 0.07 (-0.17, 0.30) |  |  |
| NS | 0.12 (-0.12, 0.36) |  |  | -0.35 (-0.58, -0.13) |  |  | 0.13 (-0.11, 0.36) |  |  | 0.46 (0.18, 0.75) |  |  |
| MCS | 0.27 (0.03, 0.50) |  |  | -0.53 (-0.76, -0.30) |  |  | 0.26 (0.02, 0.50) |  |  | 0.65 (0.35, 0.94) |  |  |
| Quad. change * cohort (ref. NCDS) |  | 2.3 | 0.685 |  | 26.0 | <0.001 |  | 12.0 | 0.017 |  | 20.4 | <0.001 |
| NSHD | -0.08 (-0.21, 0.05) |  |  | -0.02 (-0.13, 0.10) |  |  | 0.06 (-0.10, 0.23) |  |  | -0.02 (-0.28, 0.24) |  |  |
| BCS | -0.01 (-0.08, 0.07) |  |  | 0.09 (0.02, 0.16) |  |  | -0.10 (-0.19, -0.02) |  |  | -0.02 (-0.13, 0.09) |  |  |
| NS | -0.04 (-0.15, 0.07) |  |  | 0.13 (0.03, 0.23) |  |  | -0.08 (-0.18, 0.03) |  |  | -0.15 (-0.28, -0.02) |  |  |
| MCS | -0.05 (-0.16, 0.06) |  |  | 0.25 (0.14, 0.36) |  |  | -0.15 (-0.26, -0.04) |  |  | -0.28 (-0.41, -0.14) |  |  |
| Pre-pandemic financial situation (ref. Doing all right) |  | 102.2 | <0.001 |  | 170.5 | <0.001 |  | 215.1 | <.001 |  | 254.5 | <0.001 |
| Experiencing difficulties | 0.56 (0.38, 0.74) |  |  | 0.82 (0.63, 1.00) |  |  | 0.96 (0.75, 1.16) |  |  | -1.39 (-1.66, -1.13) |  |  |
| Living comfortably | -0.23 (-0.31, -0.15) |  |  | -0.26 (-0.33, -0.19) |  |  | -0.37 (-0.46, -0.27) |  |  | 0.52 (0.40, 0.63) |  |  |
| Linear change * pre-pandemic financial situation (ref. Doing all right) |  | 4.2 | 0.122 |  | 7.2 | 0.027 |  | 1.1 | 0.592 |  | 9.8 | 0.007 |
| Experiencing difficulties | 0.19 (-0.11, 0.49) |  |  | -0.11 (-0.41, 0.19) |  |  | -0.15 (-0.45, 0.14) |  |  | 0.23 (-0.18, 0.65) |  |  |
| Living comfortably | -0.08 (-0.21, 0.06) |  |  | -0.17 (-0.29, -0.05) |  |  | -0.04 (-0.19, 0.11) |  |  | 0.31 (0.12, 0.51) |  |  |
| Quad. change * pre-pandemic financial situation (ref. Doing all right) |  | 5.8 | 0.055 |  | 5.7 | 0.058 |  | 0.4 | 0.824 |  | 16.6 | <0.001 |
| Experiencing difficulties | -0.11 (-0.24, 0.03) |  |  | 0.05 (-0.09, 0.18) |  |  | 0.04 (-0.09, 0.18) |  |  | -0.06 (-0.25, 0.14) |  |  |
| Living comfortably | 0.04 (-0.03, 0.10) |  |  | 0.07 (0.01, 0.13) |  |  | 0.01 (-0.06, 0.08) |  |  | -0.19 (-0.28, -0.10) |  |  |
| Cohort (ref. NCDS) * pre-pandemic financial situation (ref. Doing all right) |  | 6.2 | 0.621 |  | 4.0 | 0.858 |  | 15.2 | 0.055 |  | 9.0 | 0.339 |
| NSHD * Experiencing difficulties | 0.05 (-0.56, 0.67) |  |  | -0.13 (-0.78, 0.52) |  |  | -0.47 (-1.11, 0.16) |  |  | 0.22 (-0.85, 1.29) |  |  |
| NSHD * Living comfortably | 0.10 (-0.07, 0.26) |  |  | -0.05 (-0.22, 0.12) |  |  | 0.10 (-0.14, 0.33) |  |  | -0.19 (-0.54, 0.16) |  |  |
| BCS * Experiencing difficulties | 0.02 (-0.24, 0.27) |  |  | -0.12 (-0.38, 0.14) |  |  | -0.07 (-0.35, 0.20) |  |  | 0.22 (-0.13, 0.57) |  |  |
| BCS * Living comfortably | 0.01 (-0.11, 0.13) |  |  | -0.03 (-0.14, 0.08) |  |  | 0.02 (-0.12, 0.15) |  |  | 0.01 (-0.16, 0.18) |  |  |
| NS * Experiencing difficulties | 0.20 (-0.12, 0.51) |  |  | -0.07 (-0.38, 0.24) |  |  | -0.20 (-0.51, 0.12) |  |  | 0.30 (-0.11, 0.71) |  |  |
| NS * Living comfortably | -0.05 (-0.23, 0.13) |  |  | -0.08 (-0.25, 0.08) |  |  | -0.02 (-0.20, 0.16) |  |  | 0.10 (-0.12, 0.33) |  |  |
| MCS * Experiencing difficulties | -0.02 (-0.31, 0.27) |  |  | -0.23 (-0.51, 0.05) |  |  | -0.46 (-0.75, -0.17) |  |  | 0.50 (0.12, 0.88) |  |  |
| MCS * Living comfortably | -0.09 (-0.26, 0.08) |  |  | -0.10 (-0.26, 0.07) |  |  | 0.00 (-0.18, 0.18) |  |  | 0.10 (-0.12, 0.33) |  |  |
| Linear change * cohort (ref. NCDS) * pre-pandemic financial situation (ref. Doing all right) |  | 8.5 | 0.388 |  | 9.4 | 0.312 |  | 8.5 | 0.389 |  | 10.9 | 0.207 |
| NSHD * Experiencing difficulties | -0.37 (-1.26, 0.51) |  |  | -0.08 (-1.15, 0.99) |  |  | 0.25 (-0.47, 0.97) |  |  | 1.68 (-0.51, 3.86) |  |  |
| NSHD * Living comfortably | -0.04 (-0.34, 0.26) |  |  | 0.21 (-0.08, 0.50) |  |  | 0.01 (-0.38, 0.40) |  |  | -0.18 (-0.78, 0.43) |  |  |
| BCS * Experiencing difficulties | 0.24 (-0.22, 0.71) |  |  | 0.40 (-0.05, 0.85) |  |  | 0.16 (-0.28, 0.60) |  |  | -0.39 (-0.96, 0.19) |  |  |
| BCS * Living comfortably | 0.07 (-0.13, 0.28) |  |  | 0.22 (0.03, 0.42) |  |  | 0.03 (-0.20, 0.25) |  |  | -0.18 (-0.48, 0.12) |  |  |
| NS * Experiencing difficulties | -0.26 (-0.79, 0.27) |  |  | 0.35 (-0.16, 0.87) |  |  | 0.51 (0.01, 1.02) |  |  | -0.47 (-1.14, 0.21) |  |  |
| NS * Living comfortably | 0.01 (-0.30, 0.32) |  |  | 0.12 (-0.17, 0.42) |  |  | -0.09 (-0.39, 0.21) |  |  | -0.28 (-0.66, 0.11) |  |  |
| MCS * Experiencing difficulties | -0.25 (-0.78, 0.27) |  |  | 0.43 (-0.09, 0.96) |  |  | 0.05 (-0.46, 0.56) |  |  | -0.41 (-1.07, 0.26) |  |  |
| MCS * Living comfortably | 0.23 (-0.10, 0.57) |  |  | 0.23 (-0.10, 0.56) |  |  | 0.18 (-0.16, 0.52) |  |  | -0.51 (-0.93, -0.08) |  |  |
| Quad. change * cohort (ref. NCDS) * pre-pandemic financial situation (ref. Doing all right) |  | 8.5 | 0.385 |  | 8.1 | 0.424 |  | 13.6 | 0.093 |  | 12.3 | 0.139 |
| NSHD * Experiencing difficulties | 0.13 (-0.28, 0.54) |  |  | -0.01 (-0.53, 0.50) |  |  | -0.18 (-0.58, 0.22) |  |  | -1.09 (-2.54, 0.36) |  |  |
| NSHD * Living comfortably | 0.00 (-0.15, 0.15) |  |  | -0.07 (-0.21, 0.07) |  |  | -0.04 (-0.22, 0.15) |  |  | 0.10 (-0.19, 0.39) |  |  |
| BCS * Experiencing difficulties | -0.09 (-0.30, 0.12) |  |  | -0.16 (-0.37, 0.05) |  |  | -0.06 (-0.26, 0.14) |  |  | 0.19 (-0.08, 0.45) |  |  |
| BCS * Living comfortably | -0.03 (-0.13, 0.06) |  |  | -0.09 (-0.18, 0.00) |  |  | 0.00 (-0.11, 0.11) |  |  | 0.10 (-0.04, 0.23) |  |  |
| NS * Experiencing difficulties | 0.11 (-0.12, 0.34) |  |  | -0.17 (-0.40, 0.06) |  |  | -0.24 (-0.46, -0.02) |  |  | 0.19 (-0.11, 0.49) |  |  |
| NS * Living comfortably | 0.00 (-0.14, 0.14) |  |  | -0.02 (-0.15, 0.11) |  |  | 0.08 (-0.05, 0.22) |  |  | 0.09 (-0.09, 0.26) |  |  |
| MCS * Experiencing difficulties | 0.10 (-0.14, 0.34) |  |  | -0.23 (-0.48, 0.02) |  |  | 0.01 (-0.23, 0.25) |  |  | 0.21 (-0.09, 0.52) |  |  |
| MCS * Living comfortably | -0.14 (-0.30, 0.01) |  |  | -0.11 (-0.27, 0.04) |  |  | -0.10 (-0.26, 0.06) |  |  | 0.28 (0.08, 0.47) |  |  |

*Note.* Adjusted models included birth sex; prospectively assessed self-rated health, long-standing illness, psychological distress, and life satisfaction (life satisfaction only in models for that outcome); and household composition as covariates. Sensitivity models correspond to the unadjusted models after restricting the analytical sample to that of the adjusted models. BCS: British Cohort Study, 1970 birth cohort; GAD-2: 2-item General Anxiety Disorder questionnaire; MCS: Millennium Cohort Study, 2000 birth cohort; NCDS: National Child and Development Study, 1958 birth cohort; NS: Next Steps, 1990 cohort; NSHD: National Survey of Health and Development, 1946 birth cohort; ONS: UK Office for National Statistics; PHQ-2: 2-item Patient Health Questionnaire; UCLA-3: 3-item UCLA loneliness scale. χ2: Wald test performed to assess the overall statistical significance of the interaction terms; χ2 statistics have 4 degrees of freedom for time*cohort interaction terms, 2 degrees of freedom for time*pre-pandemic financial situation interaction terms; and 8 degrees of freedom for the rest of interaction terms.

## Table S7.2. Unadjusted and adjusted marginal mean estimates and 95% confidence intervals by pre-pandemic financial situation.

|  |  |  | **Anxiety symptomatology (GAD-2)** | **Depressive symptomatology (PHQ-2)** | **Feelings of loneliness (UCLA-3)** | **Life satisfaction (ONS single question)** |
| --- | --- | --- | --- | --- | --- | --- |
| **Cohort** | **Pre-pandemic financial situation** | **Survey wave** | **Unadjusted marginal mean (95% CI)** | **Unadjusted marginal mean (95% CI)** | **Unadjusted marginal mean (95% CI)** | **Unadjusted marginal mean (95% CI)** |
| NSHD | Experiencing difficulties | 1 |  |  |  |  |
| NSHD | Experiencing difficulties | 2 |  |  |  |  |
| NSHD | Experiencing difficulties | 3 |  |  |  |  |
| NSHD | Doing all right | 1 | 0.62 (0.49, 0.74) | 0.66 (0.52, 0.80) | 4.17 (3.99, 4.35) | 7.34 (7.08, 7.59) |
| NSHD | Doing all right | 2 | 0.80 (0.65, 0.95) | 0.66 (0.53, 0.80) | 4.05 (3.87, 4.22) | 7.16 (6.93, 7.40) |
| NSHD | Doing all right | 3 | 0.79 (0.63, 0.95) | 0.73 (0.59, 0.88) | 4.45 (4.25, 4.64) | 6.77 (6.52, 7.03) |
| NSHD | Living comfortably | 1 | 0.42 (0.36, 0.49) | 0.36 (0.30, 0.41) | 3.86 (3.77, 3.95) | 7.70 (7.57, 7.83) |
| NSHD | Living comfortably | 2 | 0.56 (0.50, 0.63) | 0.42 (0.36, 0.48) | 3.70 (3.62, 3.79) | 7.59 (7.47, 7.71) |
| NSHD | Living comfortably | 3 | 0.53 (0.46, 0.60) | 0.49 (0.42, 0.55) | 3.99 (3.89, 4.08) | 7.12 (6.99, 7.25) |
| NCDS | Experiencing difficulties | 1 | 1.43 (1.27, 1.58) | 1.59 (1.43, 1.75) | 5.20 (5.03, 5.37) | 5.90 (5.68, 6.13) |
| NCDS | Experiencing difficulties | 2 | 1.55 (1.41, 1.69) | 1.62 (1.47, 1.77) | 5.04 (4.89, 5.19) | 5.92 (5.73, 6.10) |
| NCDS | Experiencing difficulties | 3 | 1.38 (1.25, 1.52) | 1.67 (1.53, 1.81) | 5.30 (5.14, 5.45) | 5.74 (5.56, 5.93) |
| NCDS | Doing all right | 1 | 0.81 (0.74, 0.88) | 0.72 (0.66, 0.78) | 4.25 (4.17, 4.33) | 7.31 (7.21, 7.40) |
| NCDS | Doing all right | 2 | 0.87 (0.80, 0.93) | 0.79 (0.73, 0.85) | 4.17 (4.10, 4.23) | 7.19 (7.10, 7.27) |
| NCDS | Doing all right | 3 | 0.85 (0.79, 0.90) | 0.90 (0.84, 0.95) | 4.45 (4.38, 4.52) | 6.86 (6.77, 6.94) |
| NCDS | Living comfortably | 1 | 0.57 (0.53, 0.61) | 0.46 (0.42, 0.50) | 3.88 (3.83, 3.93) | 7.85 (7.78, 7.91) |
| NCDS | Living comfortably | 2 | 0.59 (0.56, 0.63) | 0.43 (0.40, 0.46) | 3.77 (3.73, 3.81) | 7.83 (7.77, 7.88) |
| NCDS | Living comfortably | 3 | 0.60 (0.56, 0.64) | 0.57 (0.54, 0.60) | 4.04 (3.99, 4.08) | 7.27 (7.21, 7.33) |
| BCS | Experiencing difficulties | 1 | 1.60 (1.45, 1.75) | 1.66 (1.51, 1.80) | 5.15 (5.00, 5.30) | 5.98 (5.79, 6.16) |
| BCS | Experiencing difficulties | 2 | 1.86 (1.73, 1.99) | 1.75 (1.61, 1.88) | 5.13 (4.99, 5.27) | 5.83 (5.67, 5.98) |
| BCS | Experiencing difficulties | 3 | 1.70 (1.58, 1.82) | 1.88 (1.75, 2.00) | 5.24 (5.11, 5.36) | 5.78 (5.63, 5.93) |
| BCS | Doing all right | 1 | 0.92 (0.85, 0.98) | 0.89 (0.83, 0.96) | 4.21 (4.14, 4.28) | 7.19 (7.10, 7.28) |
| BCS | Doing all right | 2 | 0.99 (0.94, 1.05) | 0.84 (0.78, 0.89) | 4.25 (4.18, 4.32) | 7.07 (7.00, 7.15) |
| BCS | Doing all right | 3 | 0.98 (0.92, 1.03) | 0.98 (0.92, 1.03) | 4.41 (4.35, 4.48) | 6.75 (6.67, 6.83) |
| BCS | Living comfortably | 1 | 0.69 (0.64, 0.74) | 0.59 (0.55, 0.64) | 3.87 (3.81, 3.93) | 7.71 (7.63, 7.78) |
| BCS | Living comfortably | 2 | 0.76 (0.71, 0.81) | 0.56 (0.52, 0.61) | 3.89 (3.84, 3.95) | 7.65 (7.58, 7.73) |
| BCS | Living comfortably | 3 | 0.75 (0.70, 0.80) | 0.73 (0.68, 0.78) | 4.09 (4.03, 4.15) | 7.20 (7.13, 7.28) |
| NS | Experiencing difficulties | 1 | 2.16 (1.95, 2.37) | 2.12 (1.92, 2.33) | 5.41 (5.21, 5.61) | 5.79 (5.53, 6.05) |
| NS | Experiencing difficulties | 2 | 2.27 (2.12, 2.42) | 2.14 (1.99, 2.29) | 5.51 (5.37, 5.66) | 5.76 (5.58, 5.94) |
| NS | Experiencing difficulties | 3 | 2.22 (2.08, 2.36) | 2.17 (2.04, 2.31) | 5.51 (5.39, 5.64) | 5.61 (5.45, 5.78) |
| NS | Doing all right | 1 | 1.46 (1.35, 1.57) | 1.41 (1.30, 1.52) | 4.65 (4.54, 4.76) | 6.76 (6.62, 6.89) |
| NS | Doing all right | 2 | 1.58 (1.49, 1.67) | 1.24 (1.16, 1.32) | 4.64 (4.55, 4.72) | 6.93 (6.83, 7.03) |
| NS | Doing all right | 3 | 1.60 (1.51, 1.68) | 1.43 (1.36, 1.51) | 4.80 (4.72, 4.89) | 6.61 (6.51, 6.70) |
| NS | Living comfortably | 1 | 1.17 (1.07, 1.27) | 1.10 (1.00, 1.19) | 4.27 (4.17, 4.36) | 7.37 (7.24, 7.49) |
| NS | Living comfortably | 2 | 1.29 (1.20, 1.37) | 0.92 (0.84, 0.99) | 4.22 (4.15, 4.30) | 7.49 (7.39, 7.59) |
| NS | Living comfortably | 3 | 1.32 (1.24, 1.40) | 1.19 (1.11, 1.26) | 4.51 (4.44, 4.59) | 6.92 (6.82, 7.02) |
| MCS | Experiencing difficulties | 1 | 2.31 (2.12, 2.50) | 2.51 (2.33, 2.69) | 5.66 (5.49, 5.84) | 5.23 (5.00, 5.46) |
| MCS | Experiencing difficulties | 2 | 2.56 (2.39, 2.73) | 2.40 (2.23, 2.56) | 5.68 (5.52, 5.85) | 5.44 (5.25, 5.64) |
| MCS | Experiencing difficulties | 3 | 2.58 (2.44, 2.73) | 2.52 (2.39, 2.66) | 5.80 (5.66, 5.94) | 5.27 (5.10, 5.45) |
| MCS | Doing all right | 1 | 1.79 (1.69, 1.89) | 1.93 (1.83, 2.02) | 5.21 (5.11, 5.31) | 6.10 (5.97, 6.23) |
| MCS | Doing all right | 2 | 2.06 (1.96, 2.15) | 1.67 (1.58, 1.76) | 5.21 (5.12, 5.31) | 6.36 (6.25, 6.46) |
| MCS | Doing all right | 3 | 2.15 (2.06, 2.23) | 2.01 (1.93, 2.09) | 5.30 (5.22, 5.38) | 5.86 (5.76, 5.96) |
| MCS | Living comfortably | 1 | 1.46 (1.35, 1.57) | 1.54 (1.44, 1.65) | 4.83 (4.72, 4.94) | 6.73 (6.59, 6.87) |
| MCS | Living comfortably | 2 | 1.78 (1.68, 1.89) | 1.33 (1.23, 1.42) | 4.89 (4.78, 5.00) | 6.87 (6.75, 6.99) |
| MCS | Living comfortably | 3 | 1.74 (1.65, 1.83) | 1.64 (1.55, 1.73) | 4.90 (4.81, 4.99) | 6.44 (6.33, 6.55) |
| **Cohort** | **Pre-pandemic financial situation** | **Survey wave** | **Adjusted marginal mean (95% CI)** | **Adjusted marginal mean (95% CI)** | **Adjusted marginal mean (95% CI)** | **Adjusted marginal mean (95% CI)** |
| NSHD | Experiencing difficulties | 1 |  |  |  |  |
| NSHD | Experiencing difficulties | 2 |  |  |  |  |
| NSHD | Experiencing difficulties | 3 |  |  |  |  |
| NSHD | Doing all right | 1 | 0.52 (0.40, 0.64) | 0.61 (0.47, 0.75) | 4.16 (3.97, 4.35) | 7.29 (7.00, 7.59) |
| NSHD | Doing all right | 2 | 0.74 (0.59, 0.89) | 0.62 (0.48, 0.75) | 4.06 (3.88, 4.25) | 7.14 (6.86, 7.42) |
| NSHD | Doing all right | 3 | 0.71 (0.54, 0.88) | 0.65 (0.51, 0.79) | 4.44 (4.23, 4.65) | 6.78 (6.48, 7.09) |
| NSHD | Living comfortably | 1 | 0.47 (0.40, 0.54) | 0.42 (0.36, 0.48) | 4.04 (3.95, 4.14) | 7.30 (7.15, 7.44) |
| NSHD | Living comfortably | 2 | 0.61 (0.54, 0.69) | 0.48 (0.41, 0.55) | 3.89 (3.80, 3.98) | 7.19 (7.06, 7.33) |
| NSHD | Living comfortably | 3 | 0.58 (0.50, 0.66) | 0.56 (0.49, 0.63) | 4.16 (4.06, 4.26) | 6.70 (6.55, 6.85) |
| NCDS | Experiencing difficulties | 1 | 1.13 (0.97, 1.28) | 1.27 (1.10, 1.43) | 4.91 (4.74, 5.09) | 6.32 (6.09, 6.56) |
| NCDS | Experiencing difficulties | 2 | 1.27 (1.12, 1.41) | 1.26 (1.11, 1.40) | 4.72 (4.57, 4.87) | 6.38 (6.19, 6.57) |
| NCDS | Experiencing difficulties | 3 | 1.11 (0.97, 1.25) | 1.40 (1.25, 1.55) | 4.96 (4.81, 5.12) | 6.15 (5.95, 6.35) |
| NCDS | Doing all right | 1 | 0.80 (0.73, 0.87) | 0.71 (0.65, 0.77) | 4.28 (4.20, 4.36) | 7.29 (7.20, 7.39) |
| NCDS | Doing all right | 2 | 0.87 (0.80, 0.93) | 0.77 (0.71, 0.83) | 4.21 (4.13, 4.28) | 7.16 (7.07, 7.24) |
| NCDS | Doing all right | 3 | 0.85 (0.79, 0.90) | 0.89 (0.83, 0.95) | 4.48 (4.41, 4.55) | 6.85 (6.76, 6.94) |
| NCDS | Living comfortably | 1 | 0.67 (0.62, 0.71) | 0.58 (0.54, 0.62) | 4.08 (4.03, 4.13) | 7.51 (7.44, 7.58) |
| NCDS | Living comfortably | 2 | 0.69 (0.65, 0.73) | 0.55 (0.51, 0.58) | 3.98 (3.93, 4.03) | 7.50 (7.44, 7.56) |
| NCDS | Living comfortably | 3 | 0.71 (0.67, 0.75) | 0.70 (0.66, 0.74) | 4.24 (4.19, 4.29) | 6.94 (6.88, 7.01) |
| BCS | Experiencing difficulties | 1 | 1.16 (1.01, 1.32) | 1.27 (1.12, 1.43) | 4.79 (4.63, 4.95) | 6.41 (6.22, 6.60) |
| BCS | Experiencing difficulties | 2 | 1.45 (1.31, 1.59) | 1.35 (1.21, 1.50) | 4.79 (4.65, 4.93) | 6.30 (6.14, 6.47) |
| BCS | Experiencing difficulties | 3 | 1.25 (1.13, 1.38) | 1.45 (1.32, 1.58) | 4.89 (4.76, 5.03) | 6.24 (6.09, 6.40) |
| BCS | Doing all right | 1 | 0.92 (0.85, 0.99) | 0.92 (0.85, 0.98) | 4.31 (4.23, 4.38) | 7.06 (6.97, 7.15) |
| BCS | Doing all right | 2 | 0.98 (0.92, 1.04) | 0.84 (0.78, 0.90) | 4.33 (4.25, 4.40) | 6.96 (6.88, 7.04) |
| BCS | Doing all right | 3 | 0.94 (0.88, 1.00) | 0.98 (0.92, 1.04) | 4.48 (4.41, 4.55) | 6.68 (6.59, 6.76) |
| BCS | Living comfortably | 1 | 0.81 (0.76, 0.87) | 0.77 (0.72, 0.82) | 4.13 (4.06, 4.19) | 7.30 (7.22, 7.38) |
| BCS | Living comfortably | 2 | 0.88 (0.82, 0.93) | 0.72 (0.68, 0.77) | 4.14 (4.08, 4.21) | 7.26 (7.18, 7.34) |
| BCS | Living comfortably | 3 | 0.84 (0.79, 0.89) | 0.87 (0.82, 0.93) | 4.31 (4.24, 4.37) | 6.83 (6.74, 6.91) |
| NS | Experiencing difficulties | 1 | 1.93 (1.71, 2.16) | 1.92 (1.72, 2.13) | 5.18 (4.98, 5.39) | 6.05 (5.78, 6.32) |
| NS | Experiencing difficulties | 2 | 2.02 (1.87, 2.18) | 1.88 (1.73, 2.04) | 5.31 (5.16, 5.46) | 6.12 (5.93, 6.30) |
| NS | Experiencing difficulties | 3 | 1.94 (1.80, 2.08) | 1.91 (1.77, 2.04) | 5.25 (5.12, 5.38) | 6.00 (5.83, 6.17) |
| NS | Doing all right | 1 | 1.34 (1.23, 1.46) | 1.36 (1.25, 1.47) | 4.64 (4.53, 4.75) | 6.80 (6.66, 6.93) |
| NS | Doing all right | 2 | 1.51 (1.41, 1.60) | 1.21 (1.13, 1.30) | 4.62 (4.53, 4.71) | 6.95 (6.85, 7.05) |
| NS | Doing all right | 3 | 1.49 (1.40, 1.58) | 1.36 (1.28, 1.44) | 4.78 (4.70, 4.86) | 6.66 (6.56, 6.76) |
| NS | Living comfortably | 1 | 1.19 (1.09, 1.29) | 1.15 (1.06, 1.25) | 4.41 (4.31, 4.50) | 7.09 (6.96, 7.22) |
| NS | Living comfortably | 2 | 1.31 (1.22, 1.40) | 1.00 (0.92, 1.08) | 4.34 (4.26, 4.43) | 7.21 (7.11, 7.31) |
| NS | Living comfortably | 3 | 1.34 (1.26, 1.43) | 1.25 (1.18, 1.33) | 4.66 (4.57, 4.74) | 6.64 (6.53, 6.74) |
| MCS | Experiencing difficulties | 1 | 2.19 (2.00, 2.39) | 2.36 (2.18, 2.54) | 5.36 (5.18, 5.53) | 5.81 (5.58, 6.04) |
| MCS | Experiencing difficulties | 2 | 2.41 (2.23, 2.59) | 2.29 (2.12, 2.46) | 5.34 (5.16, 5.52) | 6.04 (5.83, 6.25) |
| MCS | Experiencing difficulties | 3 | 2.45 (2.30, 2.59) | 2.41 (2.27, 2.56) | 5.49 (5.33, 5.64) | 5.82 (5.63, 6.01) |
| MCS | Doing all right | 1 | 1.77 (1.67, 1.88) | 1.91 (1.80, 2.01) | 4.96 (4.85, 5.07) | 6.54 (6.41, 6.68) |
| MCS | Doing all right | 2 | 2.07 (1.97, 2.17) | 1.70 (1.60, 1.80) | 5.01 (4.91, 5.12) | 6.75 (6.63, 6.87) |
| MCS | Doing all right | 3 | 2.16 (2.07, 2.26) | 2.04 (1.95, 2.12) | 5.11 (5.01, 5.20) | 6.23 (6.12, 6.35) |
| MCS | Living comfortably | 1 | 1.54 (1.43, 1.65) | 1.64 (1.53, 1.75) | 4.67 (4.55, 4.79) | 6.97 (6.83, 7.12) |
| MCS | Living comfortably | 2 | 1.88 (1.76, 1.99) | 1.45 (1.34, 1.55) | 4.77 (4.65, 4.89) | 7.08 (6.94, 7.21) |
| MCS | Living comfortably | 3 | 1.82 (1.72, 1.92) | 1.71 (1.62, 1.81) | 4.74 (4.63, 4.84) | 6.66 (6.54, 6.79) |

*Note.* Adjusted models included birth sex; prospectively assessed self-rated health, long-standing illness, psychological distress, and life satisfaction (life satisfaction only in models for that outcome); and household composition as covariates. BCS: British Cohort Study, 1970 birth cohort; GAD-2: 2-item General Anxiety Disorder questionnaire; MCS: Millennium Cohort Study, 2000 birth cohort; NCDS: National Child and Development Study, 1958 birth cohort; NS: Next Steps, 1990 cohort; NSHD: National Survey of Health and Development, 1946 birth cohort; ONS: UK Office for National Statistics; PHQ-2: 2-item Patient Health Questionnaire; UCLA-3: 3-item UCLA loneliness scale. Survey wave 1: May 2020; survey wave 2: September/October 2020; survey wave 3: February/March 2021. Marginal predictions on NSHD cohort members experiencing financial difficulties have been removed from the table due to the small number of observations in this group.

## Table S7.3. Difference-in-differences (change in difference between people in worse vs best pre-pandemic financial situation between first vs last time point) estimates and 95% confidence intervals.

|  | *DID* (95% CI) | *p* |
| --- | --- | --- |
| GAD-2 | 0.075 (-0.103, 0.253) | 0.410 |
| PHQ-2 | 0.084 (-0.050, 0.217) | 0.218 |
| UCLA-3 | 0.104 (-0.035, 0.243) | 0.143 |
| Life satisfaction | -0.165 (-0.560, 0.230) | 0.414 |

*Note*. DID: difference-in-differences; GAD-2: 2-item General Anxiety Disorder questionnaire; PHQ-2: 2-item Patient Health Questionnaire; UCLA-3: 3-item UCLA loneliness scale.

## Figure S7.1. Unadjusted and adjusted anxiety symptomatology (GAD-2) marginal mean estimates and 95% confidence intervals by pre-pandemic financial situation.

Adjusted models include birth sex, pre-pandemic self-reported health and long-standing illness, pre-pandemic psychological distress, and household composition.**
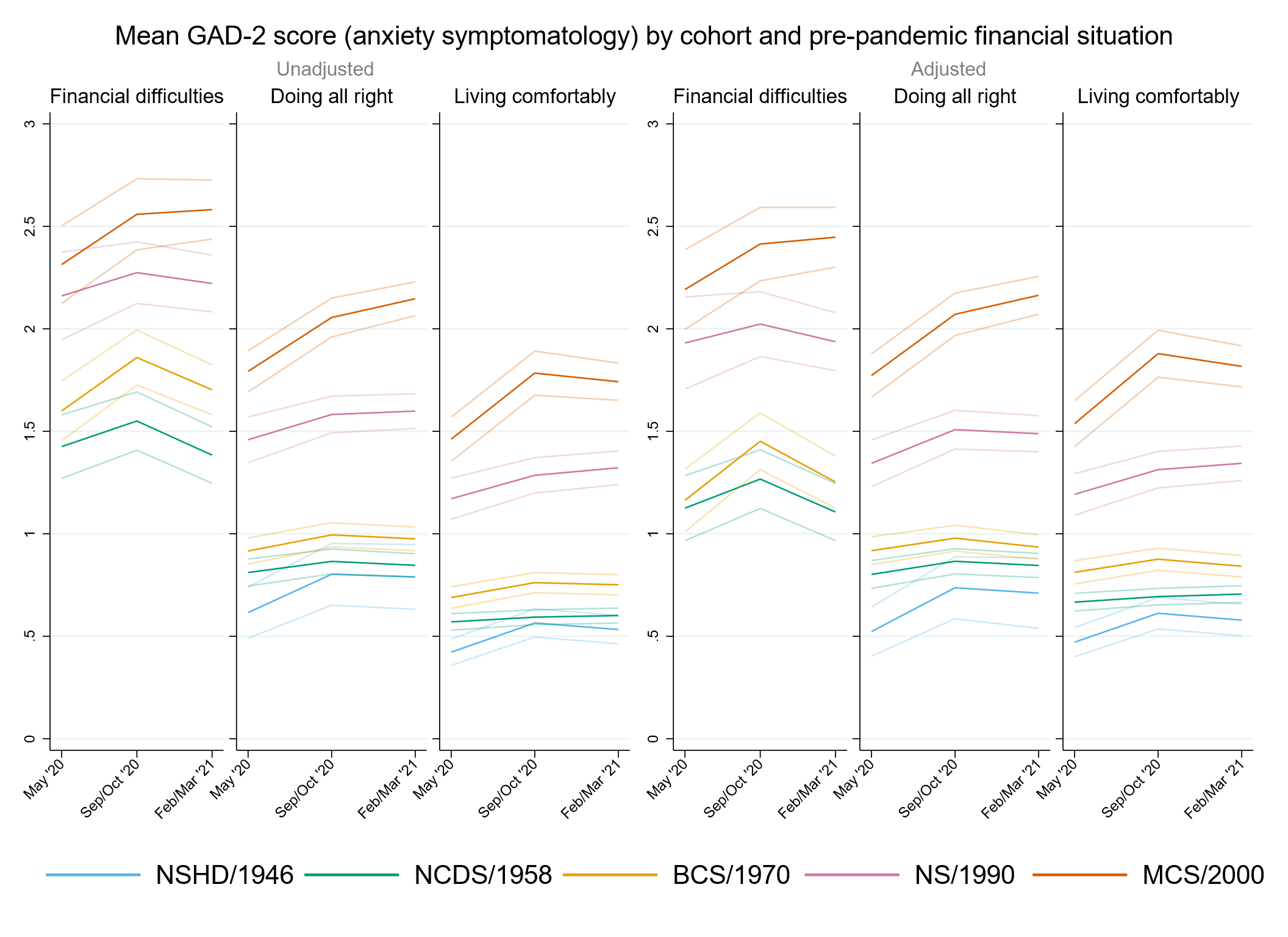
**

## Figure S7.2. Unadjusted and adjusted depressive symptomatology (PHQ-2) marginal mean estimates and 95% confidence intervals by pre-pandemic financial situation.

Adjusted models include birth sex, pre-pandemic self-reported health and long-standing illness, pre-pandemic psychological distress, and household composition.**
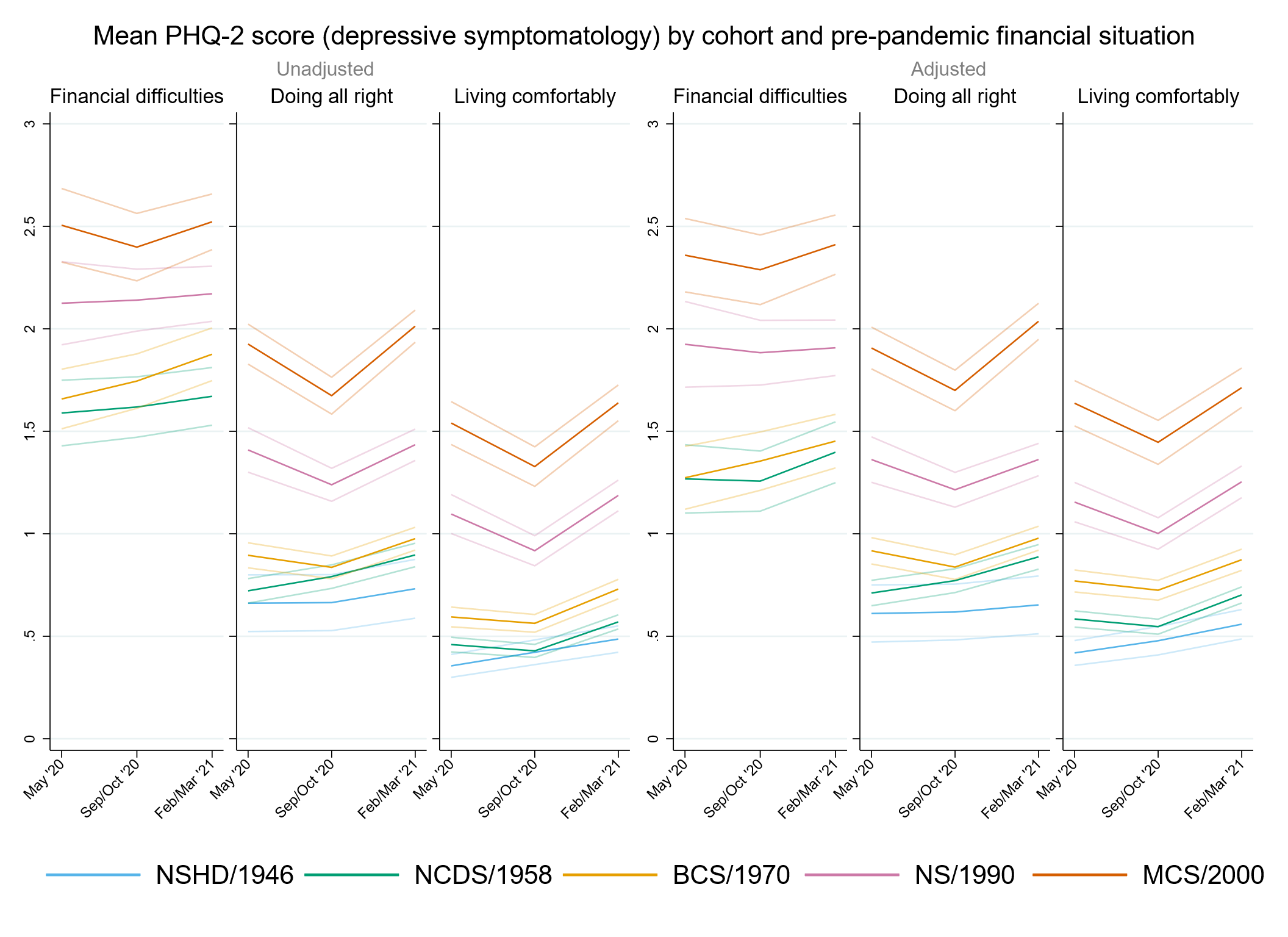
**

## Figure S7.3. Unadjusted and adjusted loneliness (UCLA-3) marginal mean estimates and 95% confidence intervals by pre-pandemic financial situation.

Adjusted models include birth sex, pre-pandemic self-reported health and long-standing illness, pre-pandemic psychological distress, and household composition.**
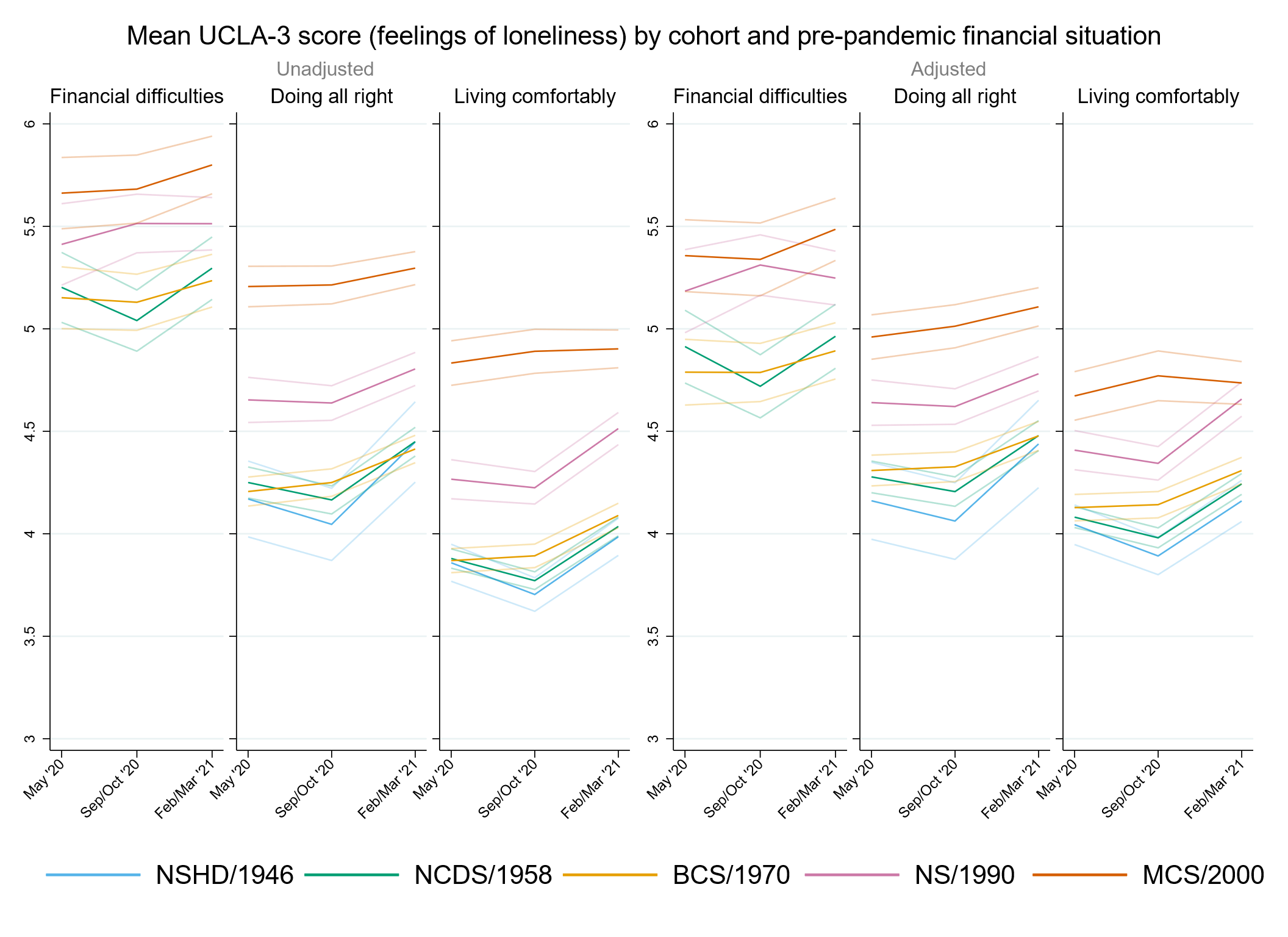
**

## Figure S7.4. Unadjusted and adjusted life satisfaction marginal mean estimates and 95% confidence intervals by pre-pandemic financial situation.

Adjusted models include birth sex, pre-pandemic self-reported health and long-standing illness, pre-pandemic life satisfaction and psychological distress, and household composition.

**
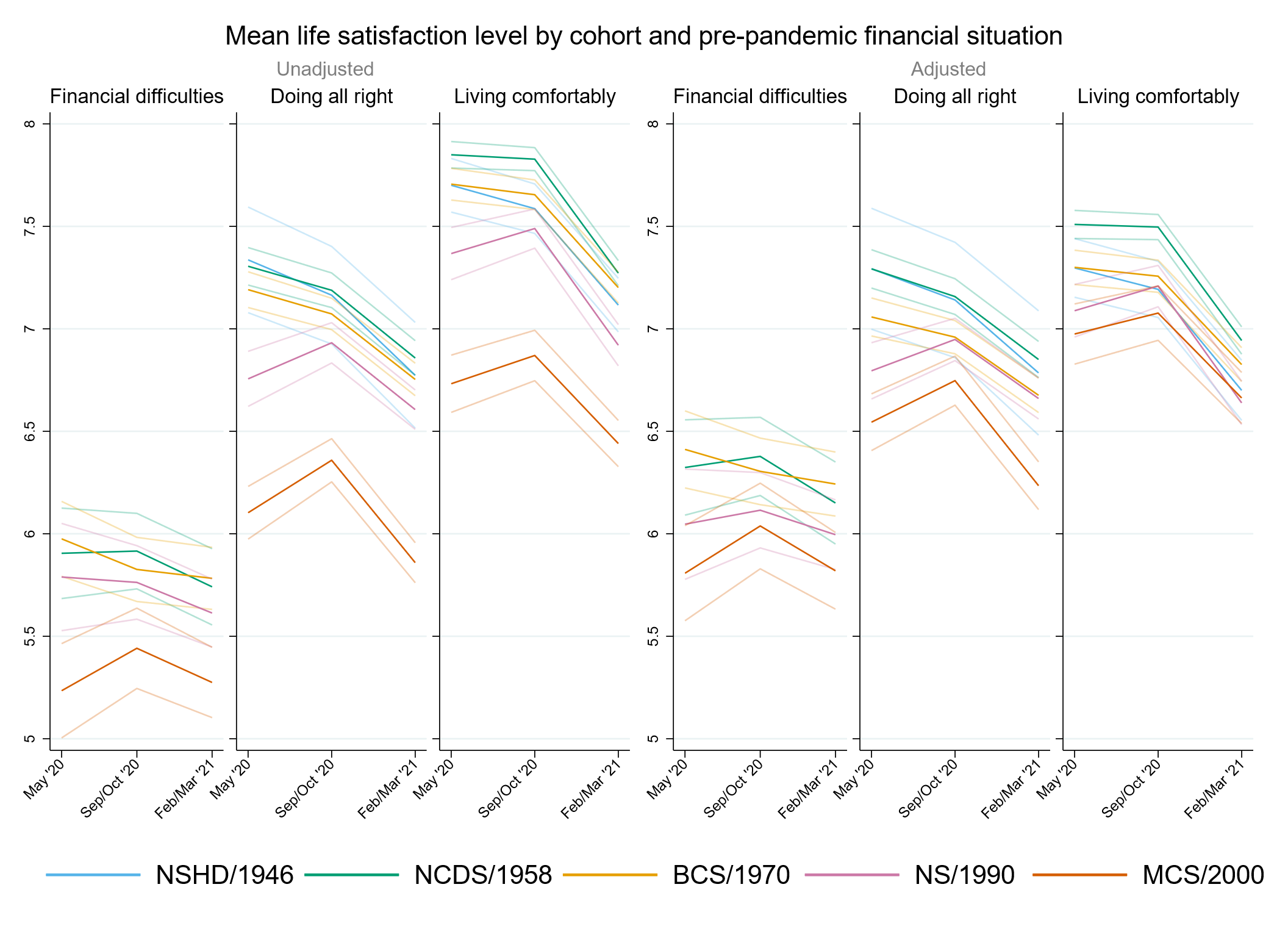
**

# Appendix S8. Results by socioeconomic position using residential Index of Multiple Deprivation (IMD) tertile as indicator.

## Table S8.1. Results of multilevel growth curve models by IMD tertile.

|  | **Anxiety symptomatology (GAD-2)** | | | **Depressive symptomatology (PHQ-2)** | | | **Feelings of loneliness (UCLA-3)** | | | **Life satisfaction (ONS single question)** | | |
| --- | --- | --- | --- | --- | --- | --- | --- | --- | --- | --- | --- | --- |
| **Unadjusted models** |  | | |  | | |  | | |  | | |
| N participants | 24,777 |  |  | 24,776 |  |  | 24,800 |  |  | 24,853 |  |  |
| N observations | 52,666 |  |  | 52,650 |  |  | 52,728 |  |  | 52,953 |  |  |
|  | ***B* (95% CI)** | **χ2** | ***p*** | ***B* (95% CI)** | **χ2** | ***p*** | ***B* (95% CI)** | **χ2** | ***p*** | ***B* (95% CI)** | **χ2** | ***p*** |
| Time (linear) | 0.04 (-0.07, 0.15) |  | 0.447 | -0.03 (-0.14, 0.07) |  | 0.549 | -0.28 (-0.41, -0.15) |  | <0.001 | 0.09 (-0.08, 0.25) |  | 0.300 |
| Time (quad) | -0.01 (-0.06, 0.04) |  | 0.762 | 0.06 (0.01, 0.11) |  | 0.020 | 0.19 (0.13, 0.24) |  | <0.001 | -0.16 (-0.24, -0.08) |  | <0.001 |
| Cohort (ref. NCDS) |  | 366.6 | <0.001 |  | 467.0 | <0.001 |  | 223.9 | <0.001 |  | 275.3 | <0.001 |
| NSHD | -0.40 (-0.53, -0.26) |  |  | -0.38 (-0.53, -0.24) |  |  | -0.27 (-0.46, -0.07) |  |  | 0.36 (0.09, 0.63) |  |  |
| BCS | 0.25 (0.14, 0.36) |  |  | 0.26 (0.15, 0.36) |  |  | 0.06 (-0.06, 0.18) |  |  | -0.28 (-0.43, -0.12) |  |  |
| NS | 0.72 (0.58, 0.85) |  |  | 0.73 (0.60, 0.86) |  |  | 0.44 (0.30, 0.57) |  |  | -0.63 (-0.80, -0.45) |  |  |
| MCS | 0.98 (0.84, 1.11) |  |  | 1.18 (1.05, 1.32) |  |  | 0.90 (0.76, 1.04) |  |  | -1.38 (-1.56, -1.20) |  |  |
| Linear change * cohort (ref. NCDS) |  | 13.6 | 0.009 |  | 11.3 | 0.023 |  | 20.5 | <0.001 |  | 19.7 | <0.001 |
| NSHD | 0.23 (0.00, 0.46) |  |  | 0.06 (-0.17, 0.29) |  |  | -0.07 (-0.39, 0.25) |  |  | -0.22 (-0.63, 0.19) |  |  |
| BCS | 0.21 (0.02, 0.39) |  |  | 0.02 (-0.16, 0.19) |  |  | 0.38 (0.18, 0.58) |  |  | -0.25 (-0.51, 0.00) |  |  |
| NS | 0.12 (-0.12, 0.35) |  |  | -0.25 (-0.47, -0.03) |  |  | 0.31 (0.09, 0.54) |  |  | 0.14 (-0.15, 0.43) |  |  |
| MCS | 0.42 (0.17, 0.68) |  |  | -0.30 (-0.56, -0.04) |  |  | 0.32 (0.06, 0.57) |  |  | 0.46 (0.13, 0.78) |  |  |
| Quad. change * cohort (ref. NCDS) |  | 9.2 | 0.057 |  | 8.3 | 0.082 |  | 22.6 | <0.001 |  | 12.1 | 0.017 |
| NSHD | -0.10 (-0.21, 0.01) |  |  | -0.04 (-0.16, 0.08) |  |  | 0.03 (-0.13, 0.18) |  |  | 0.07 (-0.12, 0.27) |  |  |
| BCS | -0.10 (-0.18, -0.01) |  |  | 0.00 (-0.09, 0.08) |  |  | -0.17 (-0.27, -0.08) |  |  | 0.12 (0.00, 0.24) |  |  |
| NS | -0.05 (-0.16, 0.05) |  |  | 0.08 (-0.02, 0.18) |  |  | -0.16 (-0.26, -0.06) |  |  | -0.03 (-0.16, 0.10) |  |  |
| MCS | -0.14 (-0.26, -0.02) |  |  | 0.14 (0.02, 0.26) |  |  | -0.19 (-0.30, -0.07) |  |  | -0.13 (-0.28, 0.02) |  |  |
| Deprivation rank tertile (ref. Lowest tertile / Most deprived) |  | 10.4 | 0.006 |  | 24.3 | <0.001 |  | 21.9 | <0.001 |  | 9.4 | 0.009 |
| Middle tertile | -0.08 (-0.17, 0.02) |  |  | -0.19 (-0.27, -0.10) |  |  | -0.17 (-0.28, -0.07) |  |  | 0.22 (0.08, 0.36) |  |  |
| Highest tertile / Least deprived | -0.15 (-0.24, -0.06) |  |  | -0.21 (-0.29, -0.12) |  |  | -0.25 (-0.36, -0.15) |  |  | 0.13 (-0.01, 0.27) |  |  |
| Linear change * deprivation rank tertile (ref. Lowest tertile / Most deprived) |  | 1.1 | 0.568 |  | 0.4 | 0.810 |  | 0.6 | 0.740 |  | 5.6 | 0.062 |
| Middle tertile | 0.05 (-0.11, 0.20) |  |  | 0.01 (-0.13, 0.15) |  |  | -0.03 (-0.20, 0.14) |  |  | -0.08 (-0.30, 0.14) |  |  |
| Highest tertile / Least deprived | 0.08 (-0.06, 0.22) |  |  | -0.03 (-0.17, 0.10) |  |  | 0.03 (-0.13, 0.18) |  |  | 0.16 (-0.06, 0.38) |  |  |
| Quad. change * deprivation rank tertile (ref. Lowest tertile / Most deprived) |  | 1.7 | 0.427 |  | 0.1 | 0.954 |  | 1.1 | 0.580 |  | 5.5 | 0.063 |
| Middle tertile | -0.03 (-0.10, 0.04) |  |  | 0.00 (-0.07, 0.06) |  |  | 0.02 (-0.06, 0.09) |  |  | 0.01 (-0.09, 0.11) |  |  |
| Highest tertile / Least deprived | -0.04 (-0.11, 0.02) |  |  | -0.01 (-0.07, 0.05) |  |  | -0.02 (-0.09, 0.05) |  |  | -0.09 (-0.19, 0.01) |  |  |
| Cohort (ref. NCDS) * deprivation rank tertile (ref. Lowest tertile / Most deprived) |  | 14.4 | 0.071 |  | 15.0 | 0.059 |  | 8.5 | 0.383 |  | 14.2 | 0.077 |
| NSHD * Middle tertile | 0.18 (-0.01, 0.36) |  |  | 0.23 (0.06, 0.41) |  |  | 0.08 (-0.17, 0.33) |  |  | -0.36 (-0.71, -0.01) |  |  |
| NSHD * Highest tertile / Least deprived | 0.21 (0.04, 0.39) |  |  | 0.23 (0.05, 0.42) |  |  | 0.24 (0.00, 0.48) |  |  | -0.34 (-0.68, 0.01) |  |  |
| BCS * Middle tertile | -0.04 (-0.19, 0.10) |  |  | -0.01 (-0.15, 0.13) |  |  | 0.02 (-0.14, 0.19) |  |  | -0.04 (-0.25, 0.17) |  |  |
| BCS * Highest tertile / Least deprived | -0.11 (-0.25, 0.03) |  |  | -0.07 (-0.21, 0.06) |  |  | 0.01 (-0.15, 0.17) |  |  | 0.09 (-0.11, 0.30) |  |  |
| NS * Middle tertile | 0.04 (-0.16, 0.24) |  |  | 0.10 (-0.09, 0.29) |  |  | 0.03 (-0.17, 0.23) |  |  | -0.12 (-0.38, 0.15) |  |  |
| NS * Highest tertile / Least deprived | -0.08 (-0.28, 0.12) |  |  | -0.09 (-0.28, 0.10) |  |  | 0.04 (-0.17, 0.24) |  |  | 0.15 (-0.11, 0.41) |  |  |
| MCS * Middle tertile | 0.04 (-0.16, 0.23) |  |  | 0.03 (-0.16, 0.22) |  |  | 0.15 (-0.05, 0.35) |  |  | 0.18 (-0.08, 0.44) |  |  |
| MCS * Highest tertile / Least deprived | 0.08 (-0.11, 0.27) |  |  | 0.05 (-0.14, 0.23) |  |  | 0.20 (0.01, 0.40) |  |  | 0.13 (-0.13, 0.38) |  |  |
| Linear change * cohort (ref. NCDS) * deprivation rank tertile (ref. Lowest tertile / Most deprived) |  | 4.4 | 0.822 |  | 13.4 | 0.100 |  | 12.9 | 0.116 |  | 9.1 | 0.331 |
| NSHD * Middle tertile | -0.20 (-0.50, 0.10) |  |  | -0.17 (-0.46, 0.11) |  |  | 0.23 (-0.16, 0.63) |  |  | 0.46 (-0.10, 1.01) |  |  |
| NSHD * Highest tertile / Least deprived | -0.03 (-0.34, 0.27) |  |  | 0.19 (-0.11, 0.48) |  |  | -0.21 (-0.60, 0.18) |  |  | 0.00 (-0.54, 0.54) |  |  |
| BCS * Middle tertile | -0.18 (-0.43, 0.07) |  |  | -0.12 (-0.35, 0.11) |  |  | -0.19 (-0.45, 0.07) |  |  | 0.30 (-0.03, 0.64) |  |  |
| BCS * Highest tertile / Least deprived | -0.13 (-0.36, 0.11) |  |  | -0.10 (-0.32, 0.12) |  |  | -0.28 (-0.53, -0.02) |  |  | 0.16 (-0.18, 0.49) |  |  |
| NS * Middle tertile | -0.04 (-0.38, 0.30) |  |  | -0.03 (-0.36, 0.29) |  |  | -0.13 (-0.45, 0.19) |  |  | 0.18 (-0.24, 0.60) |  |  |
| NS * Highest tertile / Least deprived | 0.10 (-0.24, 0.43) |  |  | 0.06 (-0.26, 0.38) |  |  | -0.14 (-0.47, 0.19) |  |  | 0.10 (-0.32, 0.52) |  |  |
| MCS * Middle tertile | -0.12 (-0.50, 0.25) |  |  | -0.05 (-0.42, 0.33) |  |  | 0.04 (-0.33, 0.41) |  |  | -0.22 (-0.69, 0.25) |  |  |
| MCS * Highest tertile / Least deprived | -0.11 (-0.45, 0.24) |  |  | -0.27 (-0.61, 0.07) |  |  | -0.10 (-0.45, 0.25) |  |  | -0.01 (-0.46, 0.45) |  |  |
| Quad. change * cohort (ref. NCDS) * deprivation rank tertile (ref. Lowest tertile / Most deprived) |  | 4.4 | 0.822 |  | 10.8 | 0.214 |  | 13.4 | 0.099 |  | 4.5 | 0.811 |
| NSHD * Middle tertile | 0.09 (-0.05, 0.23) |  |  | 0.06 (-0.08, 0.20) |  |  | -0.11 (-0.29, 0.08) |  |  | -0.17 (-0.43, 0.09) |  |  |
| NSHD * Highest tertile / Least deprived | 0.03 (-0.12, 0.17) |  |  | -0.07 (-0.22, 0.07) |  |  | 0.11 (-0.07, 0.29) |  |  | -0.01 (-0.26, 0.25) |  |  |
| BCS * Middle tertile | 0.09 (-0.02, 0.20) |  |  | 0.04 (-0.07, 0.15) |  |  | 0.07 (-0.05, 0.19) |  |  | -0.10 (-0.25, 0.06) |  |  |
| BCS * Highest tertile / Least deprived | 0.07 (-0.04, 0.18) |  |  | 0.06 (-0.04, 0.16) |  |  | 0.13 (0.01, 0.24) |  |  | -0.06 (-0.21, 0.10) |  |  |
| NS * Middle tertile | 0.05 (-0.10, 0.20) |  |  | 0.03 (-0.11, 0.18) |  |  | 0.07 (-0.07, 0.21) |  |  | -0.05 (-0.23, 0.14) |  |  |
| NS * Highest tertile / Least deprived | 0.00 (-0.15, 0.15) |  |  | 0.05 (-0.09, 0.19) |  |  | 0.08 (-0.06, 0.23) |  |  | -0.04 (-0.23, 0.14) |  |  |
| MCS * Middle tertile | 0.10 (-0.07, 0.28) |  |  | 0.04 (-0.14, 0.21) |  |  | 0.00 (-0.17, 0.17) |  |  | 0.05 (-0.16, 0.26) |  |  |
| MCS * Highest tertile / Least deprived | 0.03 (-0.14, 0.19) |  |  | 0.13 (-0.03, 0.29) |  |  | 0.05 (-0.11, 0.21) |  |  | -0.01 (-0.22, 0.19) |  |  |
| **Adjusted models** |  | | |  | | |  | | |  | | |
| N participants | 21,739 |  |  | 21,740 |  |  | 21,755 |  |  | 21,549 |  |  |
| N observations | 47,194 |  |  | 47,184 |  |  | 47,248 |  |  | 46,834 |  |  |
|  | ***B* (95% CI)** | **χ2** | ***p*** | ***B* (95% CI)** | **χ2** | ***p*** | ***B* (95% CI)** | **χ2** | ***p*** | ***B* (95% CI)** | **χ2** | ***p*** |
| Time (linear) | 0.02 (-0.09, 0.14) |  | 0.688 | -0.10 (-0.21, 0.01) |  | 0.072 | -0.30 (-0.43, -0.17) |  | <0.001 | 0.18 (0.01, 0.36) |  | 0.041 |
| Time (quad) | 0.00 (-0.05, 0.05) |  | 0.994 | 0.10 (0.04, 0.15) |  | <0.001 | 0.19 (0.13, 0.26) |  | <0.001 | -0.19 (-0.28, -0.11) |  | <0.001 |
| Cohort (ref. NCDS) |  | 295.7 | <0.001 |  | 407.2 | <0.001 |  | 109.6 | <0.001 |  | 95.0 | <0.001 |
| NSHD | -0.34 (-0.48, -0.20) |  |  | -0.33 (-0.47, -0.19) |  |  | -0.19 (-0.38, 0.01) |  |  | 0.13 (-0.14, 0.40) |  |  |
| BCS | 0.19 (0.08, 0.30) |  |  | 0.22 (0.11, 0.32) |  |  | 0.01 (-0.11, 0.13) |  |  | -0.22 (-0.38, -0.07) |  |  |
| NS | 0.73 (0.59, 0.86) |  |  | 0.76 (0.62, 0.89) |  |  | 0.43 (0.29, 0.56) |  |  | -0.56 (-0.74, -0.38) |  |  |
| MCS | 0.94 (0.80, 1.09) |  |  | 1.14 (1.00, 1.28) |  |  | 0.61 (0.46, 0.76) |  |  | -0.83 (-1.02, -0.63) |  |  |
| Linear change * cohort (ref. NCDS) |  | 17.1 | 0.002 |  | 6.0 | 0.198 |  | 21.6 | <0.001 |  | 13.5 | 0.009 |
| NSHD | 0.27 (0.04, 0.50) |  |  | 0.11 (-0.15, 0.38) |  |  | 0.03 (-0.31, 0.36) |  |  | 0.00 (-0.44, 0.44) |  |  |
| BCS | 0.25 (0.05, 0.45) |  |  | 0.07 (-0.12, 0.26) |  |  | 0.43 (0.23, 0.64) |  |  | -0.29 (-0.57, -0.02) |  |  |
| NS | 0.13 (-0.12, 0.37) |  |  | -0.16 (-0.39, 0.08) |  |  | 0.33 (0.09, 0.57) |  |  | 0.05 (-0.25, 0.36) |  |  |
| MCS | 0.50 (0.23, 0.77) |  |  | -0.18 (-0.44, 0.09) |  |  | 0.35 (0.08, 0.62) |  |  | 0.36 (0.02, 0.70) |  |  |
| Quad. change * cohort (ref. NCDS) |  | 13.6 | 0.009 |  | 4.2 | 0.374 |  | 25.2 | <0.001 |  | 10.8 | 0.029 |
| NSHD | -0.11 (-0.22, 0.00) |  |  | -0.05 (-0.20, 0.10) |  |  | -0.01 (-0.18, 0.16) |  |  | -0.09 (-0.32, 0.15) |  |  |
| BCS | -0.14 (-0.23, -0.04) |  |  | -0.05 (-0.14, 0.04) |  |  | -0.21 (-0.31, -0.12) |  |  | 0.14 (0.02, 0.27) |  |  |
| NS | -0.06 (-0.17, 0.05) |  |  | 0.02 (-0.09, 0.12) |  |  | -0.16 (-0.27, -0.06) |  |  | 0.00 (-0.14, 0.14) |  |  |
| MCS | -0.18 (-0.30, -0.05) |  |  | 0.08 (-0.05, 0.20) |  |  | -0.20 (-0.32, -0.07) |  |  | -0.10 (-0.26, 0.05) |  |  |
| Deprivation rank tertile (ref. Lowest tertile / Most deprived) |  | 0.5 | 0.765 |  | 2.0 | 0.366 |  | 0.3 | 0.845 |  | 9.4 | 0.009 |
| Middle tertile | 0.03 (-0.06, 0.12) |  |  | -0.06 (-0.14, 0.03) |  |  | -0.03 (-0.14, 0.07) |  |  | 0.05 (-0.09, 0.18) |  |  |
| Highest tertile / Least deprived | 0.03 (-0.06, 0.12) |  |  | -0.02 (-0.10, 0.07) |  |  | -0.02 (-0.12, 0.09) |  |  | -0.14 (-0.28, 0.00) |  |  |
| Linear change * deprivation rank tertile (ref. Lowest tertile / Most deprived) |  | 2.5 | 0.284 |  | 0.8 | 0.673 |  | 0.2 | 0.912 |  | 8.0 | 0.018 |
| Middle tertile | 0.06 (-0.09, 0.22) |  |  | 0.07 (-0.08, 0.21) |  |  | 0.02 (-0.15, 0.20) |  |  | -0.20 (-0.43, 0.03) |  |  |
| Highest tertile / Least deprived | 0.12 (-0.03, 0.27) |  |  | 0.03 (-0.11, 0.18) |  |  | 0.04 (-0.13, 0.20) |  |  | 0.10 (-0.13, 0.33) |  |  |
| Quad. change * deprivation rank tertile (ref. Lowest tertile / Most deprived) |  | 3.1 | 0.211 |  | 1.6 | 0.460 |  | 0.7 | 0.714 |  | 8.0 | 0.018 |
| Middle tertile | -0.03 (-0.11, 0.04) |  |  | -0.03 (-0.10, 0.04) |  |  | -0.01 (-0.09, 0.07) |  |  | 0.07 (-0.04, 0.17) |  |  |
| Highest tertile / Least deprived | -0.06 (-0.13, 0.01) |  |  | -0.04 (-0.11, 0.03) |  |  | -0.03 (-0.11, 0.05) |  |  | -0.07 (-0.18, 0.03) |  |  |
| Cohort (ref. NCDS) * deprivation rank tertile (ref. Lowest tertile / Most deprived) |  | 12.3 | 0.140 |  | 17.5 | 0.026 |  | 6.0 | 0.645 |  | 16.1 | 0.041 |
| NSHD * Middle tertile | 0.11 (-0.07, 0.29) |  |  | 0.22 (0.05, 0.39) |  |  | 0.05 (-0.20, 0.30) |  |  | -0.20 (-0.55, 0.15) |  |  |
| NSHD * Highest tertile / Least deprived | 0.08 (-0.09, 0.25) |  |  | 0.12 (-0.05, 0.30) |  |  | 0.14 (-0.10, 0.38) |  |  | -0.26 (-0.60, 0.09) |  |  |
| BCS * Middle tertile | -0.03 (-0.17, 0.11) |  |  | 0.02 (-0.12, 0.16) |  |  | 0.07 (-0.09, 0.23) |  |  | -0.09 (-0.29, 0.11) |  |  |
| BCS * Highest tertile / Least deprived | -0.10 (-0.24, 0.03) |  |  | -0.04 (-0.18, 0.09) |  |  | 0.09 (-0.07, 0.24) |  |  | 0.01 (-0.19, 0.22) |  |  |
| NS * Middle tertile | -0.10 (-0.30, 0.10) |  |  | -0.03 (-0.22, 0.17) |  |  | 0.00 (-0.20, 0.19) |  |  | -0.11 (-0.38, 0.15) |  |  |
| NS * Highest tertile / Least deprived | -0.25 (-0.44, -0.06) |  |  | -0.26 (-0.44, -0.08) |  |  | -0.11 (-0.31, 0.09) |  |  | 0.22 (-0.03, 0.47) |  |  |
| MCS * Middle tertile | 0.02 (-0.17, 0.21) |  |  | 0.02 (-0.17, 0.20) |  |  | 0.09 (-0.11, 0.29) |  |  | 0.21 (-0.04, 0.46) |  |  |
| MCS * Highest tertile / Least deprived | 0.05 (-0.14, 0.23) |  |  | 0.00 (-0.18, 0.17) |  |  | 0.08 (-0.11, 0.27) |  |  | 0.22 (-0.03, 0.47) |  |  |
| Linear change * cohort (ref. NCDS) * deprivation rank tertile (ref. Lowest tertile / Most deprived) |  | 6.1 | 0.632 |  | 16.5 | 0.036 |  | 12.8 | 0.121 |  | 8.8 | 0.358 |
| NSHD * Middle tertile | -0.21 (-0.51, 0.10) |  |  | -0.25 (-0.57, 0.06) |  |  | 0.08 (-0.34, 0.49) |  |  | 0.04 (-0.57, 0.65) |  |  |
| NSHD * Highest tertile / Least deprived | -0.11 (-0.41, 0.20) |  |  | 0.12 (-0.21, 0.45) |  |  | -0.31 (-0.71, 0.10) |  |  | -0.15 (-0.75, 0.45) |  |  |
| BCS * Middle tertile | -0.22 (-0.49, 0.04) |  |  | -0.19 (-0.44, 0.06) |  |  | -0.30 (-0.57, -0.03) |  |  | 0.37 (0.01, 0.73) |  |  |
| BCS * Highest tertile / Least deprived | -0.19 (-0.45, 0.06) |  |  | -0.18 (-0.42, 0.06) |  |  | -0.32 (-0.58, -0.05) |  |  | 0.18 (-0.17, 0.53) |  |  |
| NS * Middle tertile | 0.01 (-0.34, 0.37) |  |  | -0.04 (-0.38, 0.30) |  |  | -0.20 (-0.54, 0.14) |  |  | 0.31 (-0.14, 0.76) |  |  |
| NS * Highest tertile / Least deprived | 0.11 (-0.24, 0.46) |  |  | 0.01 (-0.32, 0.33) |  |  | -0.18 (-0.52, 0.16) |  |  | 0.17 (-0.27, 0.60) |  |  |
| MCS * Middle tertile | -0.15 (-0.54, 0.23) |  |  | -0.07 (-0.45, 0.32) |  |  | 0.05 (-0.34, 0.44) |  |  | -0.22 (-0.70, 0.26) |  |  |
| MCS * Highest tertile / Least deprived | -0.22 (-0.58, 0.14) |  |  | -0.35 (-0.70, 0.00) |  |  | -0.08 (-0.44, 0.28) |  |  | 0.04 (-0.43, 0.51) |  |  |
| Quad. change * cohort (ref. NCDS) * deprivation rank tertile (ref. Lowest tertile / Most deprived) |  | 6.4 | 0.602 |  | 14.9 | 0.061 |  | 13.5 | 0.097 |  | 6.0 | 0.651 |
| NSHD * Middle tertile | 0.09 (-0.06, 0.23) |  |  | 0.08 (-0.10, 0.25) |  |  | -0.05 (-0.25, 0.16) |  |  | 0.07 (-0.24, 0.39) |  |  |
| NSHD * Highest tertile / Least deprived | 0.05 (-0.09, 0.20) |  |  | -0.06 (-0.24, 0.11) |  |  | 0.15 (-0.05, 0.34) |  |  | 0.14 (-0.16, 0.45) |  |  |
| BCS * Middle tertile | 0.12 (0.00, 0.24) |  |  | 0.09 (-0.03, 0.20) |  |  | 0.14 (0.01, 0.27) |  |  | -0.14 (-0.30, 0.03) |  |  |
| BCS * Highest tertile / Least deprived | 0.12 (0.00, 0.24) |  |  | 0.11 (0.00, 0.23) |  |  | 0.16 (0.04, 0.28) |  |  | -0.06 (-0.22, 0.11) |  |  |
| NS * Middle tertile | 0.03 (-0.14, 0.19) |  |  | 0.05 (-0.10, 0.20) |  |  | 0.09 (-0.06, 0.24) |  |  | -0.10 (-0.30, 0.10) |  |  |
| NS * Highest tertile / Least deprived | -0.01 (-0.17, 0.15) |  |  | 0.08 (-0.06, 0.23) |  |  | 0.11 (-0.04, 0.26) |  |  | -0.06 (-0.25, 0.14) |  |  |
| MCS * Middle tertile | 0.12 (-0.06, 0.29) |  |  | 0.05 (-0.13, 0.23) |  |  | 0.00 (-0.18, 0.18) |  |  | 0.06 (-0.17, 0.28) |  |  |
| MCS * Highest tertile / Least deprived | 0.08 (-0.08, 0.25) |  |  | 0.17 (0.01, 0.34) |  |  | 0.05 (-0.12, 0.21) |  |  | -0.03 (-0.24, 0.19) |  |  |
| **Sensitivity models** |  | | |  | | |  | | |  | | |
| N participants | 21,739 |  |  | 21,740 |  |  | 21,755 |  |  | 21,549 |  |  |
| N observations | 47,194 |  |  | 47,184 |  |  | 47,248 |  |  | 46,834 |  |  |
|  | ***B* (95% CI)** | **χ2** | ***p*** | ***B* (95% CI)** | **χ2** | ***p*** | ***B* (95% CI)** | **χ2** | ***p*** | ***B* (95% CI)** | **χ2** | ***p*** |
| Time (linear) | 0.03 (-0.08, 0.15) |  | 0.569 | -0.09 (-0.20, 0.02) |  | 0.120 | -0.28 (-0.42, -0.15) |  | <0.001 | 0.15 (-0.02, 0.33) |  | 0.092 |
| Time (quad) | 0.00 (-0.06, 0.05) |  | 0.917 | 0.09 (0.04, 0.14) |  | 0.001 | 0.19 (0.13, 0.25) |  | <0.001 | -0.19 (-0.27, -0.11) |  | <0.001 |
| Cohort (ref. NCDS) |  | 368.0 | <0.001 |  | 494.1 | <0.001 |  | 218.1 | <0.001 |  | 235.7 | <0.001 |
| NSHD | -0.40 (-0.54, -0.26) |  |  | -0.43 (-0.56, -0.29) |  |  | -0.30 (-0.50, -0.11) |  |  | 0.33 (0.03, 0.63) |  |  |
| BCS | 0.27 (0.15, 0.38) |  |  | 0.27 (0.15, 0.38) |  |  | 0.05 (-0.09, 0.18) |  |  | -0.27 (-0.44, -0.10) |  |  |
| NS | 0.77 (0.63, 0.92) |  |  | 0.76 (0.62, 0.90) |  |  | 0.45 (0.31, 0.59) |  |  | -0.61 (-0.79, -0.42) |  |  |
| MCS | 0.98 (0.84, 1.13) |  |  | 1.17 (1.03, 1.31) |  |  | 0.89 (0.75, 1.04) |  |  | -1.34 (-1.53, -1.15) |  |  |
| Linear change * cohort (ref. NCDS) |  | 15.6 | 0.004 |  | 6.8 | 0.147 |  | 21.3 | <0.001 |  | 14.0 | 0.007 |
| NSHD | 0.26 (0.03, 0.49) |  |  | 0.10 (-0.16, 0.37) |  |  | 0.02 (-0.31, 0.35) |  |  | 0.03 (-0.42, 0.47) |  |  |
| BCS | 0.26 (0.06, 0.46) |  |  | 0.08 (-0.11, 0.27) |  |  | 0.43 (0.23, 0.64) |  |  | -0.30 (-0.58, -0.03) |  |  |
| NS | 0.12 (-0.13, 0.36) |  |  | -0.17 (-0.41, 0.06) |  |  | 0.33 (0.09, 0.57) |  |  | 0.06 (-0.25, 0.37) |  |  |
| MCS | 0.47 (0.20, 0.74) |  |  | -0.20 (-0.47, 0.06) |  |  | 0.33 (0.06, 0.60) |  |  | 0.37 (0.03, 0.71) |  |  |
| Quad. change * cohort (ref. NCDS) |  | 13.1 | 0.011 |  | 4.5 | 0.3 |  | 24.8 | <0.001 |  | 10.5 | 0.033 |
| NSHD | -0.11 (-0.22, 0.00) |  |  | -0.04 (-0.19, 0.11) |  |  | -0.01 (-0.18, 0.16) |  |  | -0.09 (-0.33, 0.15) |  |  |
| BCS | -0.14 (-0.23, -0.04) |  |  | -0.05 (-0.14, 0.04) |  |  | -0.21 (-0.31, -0.12) |  |  | 0.15 (0.02, 0.27) |  |  |
| NS | -0.06 (-0.17, 0.05) |  |  | 0.02 (-0.08, 0.13) |  |  | -0.17 (-0.28, -0.06) |  |  | 0.00 (-0.14, 0.14) |  |  |
| MCS | -0.17 (-0.30, -0.05) |  |  | 0.08 (-0.04, 0.21) |  |  | -0.19 (-0.32, -0.07) |  |  | -0.09 (-0.25, 0.06) |  |  |
| Deprivation rank tertile (ref. Lowest tertile / Most deprived) |  | 7.5 | 0.023 |  | 23.0 | <0.001 |  | 21.6 | <0.001 |  | 11.3 | 0.004 |
| Middle tertile | -0.09 (-0.19, 0.01) |  |  | -0.20 (-0.29, -0.11) |  |  | -0.20 (-0.31, -0.08) |  |  | 0.25 (0.10, 0.39) |  |  |
| Highest tertile / Least deprived | -0.13 (-0.22, -0.04) |  |  | -0.20 (-0.30, -0.11) |  |  | -0.26 (-0.37, -0.15) |  |  | 0.13 (-0.01, 0.28) |  |  |
| Linear change * deprivation rank tertile (ref. Lowest tertile / Most deprived) |  | 1.9 | 0.382 |  | 0.9 | 0.633 |  | 0.1 | 0.953 |  | 9.1 | 0.010 |
| Middle tertile | 0.07 (-0.09, 0.22) |  |  | 0.07 (-0.08, 0.21) |  |  | 0.02 (-0.15, 0.20) |  |  | -0.20 (-0.43, 0.03) |  |  |
| Highest tertile / Least deprived | 0.11 (-0.04, 0.26) |  |  | 0.02 (-0.12, 0.16) |  |  | 0.02 (-0.14, 0.19) |  |  | 0.12 (-0.11, 0.35) |  |  |
| Quad. change * deprivation rank tertile (ref. Lowest tertile / Most deprived) |  | 2.6 | 0.270 |  | 1.2 | 0.546 |  | 0.5 | 0.785 |  | 8.5 | 0.014 |
| Middle tertile | -0.03 (-0.11, 0.04) |  |  | -0.03 (-0.10, 0.04) |  |  | -0.01 (-0.09, 0.07) |  |  | 0.06 (-0.04, 0.17) |  |  |
| Highest tertile / Least deprived | -0.06 (-0.13, 0.01) |  |  | -0.04 (-0.10, 0.03) |  |  | -0.03 (-0.10, 0.05) |  |  | -0.08 (-0.19, 0.03) |  |  |
| Cohort (ref. NCDS) * deprivation rank tertile (ref. Lowest tertile / Most deprived) |  | 18.8 | 0.016 |  | 25.6 | 0.001 |  | 9.8 | 0.278 |  | 15.5 | 0.050 |
| NSHD * Middle tertile | 0.23 (0.04, 0.41) |  |  | 0.34 (0.17, 0.51) |  |  | 0.17 (-0.09, 0.42) |  |  | -0.30 (-0.68, 0.09) |  |  |
| NSHD * Highest tertile / Least deprived | 0.23 (0.05, 0.41) |  |  | 0.27 (0.10, 0.45) |  |  | 0.29 (0.04, 0.54) |  |  | -0.42 (-0.80, -0.04) |  |  |
| BCS * Middle tertile | -0.05 (-0.21, 0.10) |  |  | 0.00 (-0.15, 0.14) |  |  | 0.03 (-0.14, 0.20) |  |  | -0.04 (-0.26, 0.18) |  |  |
| BCS * Highest tertile / Least deprived | -0.13 (-0.28, 0.02) |  |  | -0.07 (-0.21, 0.08) |  |  | 0.04 (-0.13, 0.21) |  |  | 0.09 (-0.13, 0.30) |  |  |
| NS * Middle tertile | -0.02 (-0.23, 0.19) |  |  | 0.05 (-0.15, 0.26) |  |  | 0.06 (-0.15, 0.27) |  |  | -0.18 (-0.46, 0.10) |  |  |
| NS * Highest tertile / Least deprived | -0.16 (-0.36, 0.04) |  |  | -0.16 (-0.35, 0.03) |  |  | 0.00 (-0.21, 0.21) |  |  | 0.17 (-0.10, 0.43) |  |  |
| MCS * Middle tertile | 0.05 (-0.15, 0.25) |  |  | 0.06 (-0.13, 0.26) |  |  | 0.16 (-0.04, 0.37) |  |  | 0.14 (-0.13, 0.41) |  |  |
| MCS * Highest tertile / Least deprived | 0.08 (-0.12, 0.28) |  |  | 0.06 (-0.12, 0.25) |  |  | 0.22 (0.02, 0.42) |  |  | 0.10 (-0.17, 0.36) |  |  |
| Linear change * cohort (ref. NCDS) * deprivation rank tertile (ref. Lowest tertile / Most deprived) |  | 6.1 | 0.635 |  | 16.3 | 0.039 |  | 12.5 | 0.131 |  | 9.0 | 0.339 |
| NSHD * Middle tertile | -0.20 (-0.51, 0.11) |  |  | -0.24 (-0.56, 0.07) |  |  | 0.08 (-0.34, 0.49) |  |  | 0.03 (-0.59, 0.64) |  |  |
| NSHD * Highest tertile / Least deprived | -0.08 (-0.39, 0.23) |  |  | 0.15 (-0.18, 0.48) |  |  | -0.29 (-0.70, 0.11) |  |  | -0.17 (-0.78, 0.43) |  |  |
| BCS * Middle tertile | -0.23 (-0.50, 0.03) |  |  | -0.19 (-0.44, 0.07) |  |  | -0.30 (-0.57, -0.03) |  |  | 0.40 (0.04, 0.76) |  |  |
| BCS * Highest tertile / Least deprived | -0.21 (-0.47, 0.05) |  |  | -0.19 (-0.43, 0.05) |  |  | -0.33 (-0.60, -0.06) |  |  | 0.19 (-0.17, 0.55) |  |  |
| NS * Middle tertile | 0.00 (-0.36, 0.36) |  |  | -0.04 (-0.38, 0.31) |  |  | -0.19 (-0.54, 0.15) |  |  | 0.32 (-0.14, 0.77) |  |  |
| NS * Highest tertile / Least deprived | 0.12 (-0.24, 0.47) |  |  | 0.03 (-0.30, 0.36) |  |  | -0.18 (-0.52, 0.17) |  |  | 0.15 (-0.29, 0.59) |  |  |
| MCS * Middle tertile | -0.16 (-0.55, 0.23) |  |  | -0.07 (-0.47, 0.32) |  |  | 0.05 (-0.35, 0.44) |  |  | -0.18 (-0.67, 0.30) |  |  |
| MCS * Highest tertile / Least deprived | -0.18 (-0.54, 0.18) |  |  | -0.32 (-0.68, 0.03) |  |  | -0.06 (-0.43, 0.30) |  |  | 0.05 (-0.43, 0.52) |  |  |
| Quad. change * cohort (ref. NCDS) * deprivation rank tertile (ref. Lowest tertile / Most deprived) |  | 6.7 | 0.566 |  | 14.3 | 0.074 |  | 13.1 | 0.109 |  | 5.9 | 0.661 |
| NSHD * Middle tertile | 0.08 (-0.06, 0.23) |  |  | 0.07 (-0.10, 0.24) |  |  | -0.05 (-0.25, 0.16) |  |  | 0.08 (-0.24, 0.40) |  |  |
| NSHD * Highest tertile / Least deprived | 0.05 (-0.10, 0.19) |  |  | -0.07 (-0.24, 0.11) |  |  | 0.14 (-0.06, 0.34) |  |  | 0.15 (-0.16, 0.46) |  |  |
| BCS * Middle tertile | 0.12 (0.00, 0.24) |  |  | 0.08 (-0.03, 0.20) |  |  | 0.14 (0.01, 0.26) |  |  | -0.14 (-0.31, 0.03) |  |  |
| BCS * Highest tertile / Least deprived | 0.12 (0.01, 0.24) |  |  | 0.11 (0.00, 0.23) |  |  | 0.16 (0.04, 0.29) |  |  | -0.06 (-0.23, 0.11) |  |  |
| NS * Middle tertile | 0.03 (-0.13, 0.19) |  |  | 0.05 (-0.10, 0.20) |  |  | 0.09 (-0.06, 0.24) |  |  | -0.10 (-0.31, 0.10) |  |  |
| NS * Highest tertile / Least deprived | -0.01 (-0.17, 0.15) |  |  | 0.08 (-0.07, 0.22) |  |  | 0.11 (-0.05, 0.26) |  |  | -0.05 (-0.25, 0.14) |  |  |
| MCS * Middle tertile | 0.12 (-0.06, 0.30) |  |  | 0.05 (-0.13, 0.23) |  |  | 0.00 (-0.18, 0.18) |  |  | 0.04 (-0.18, 0.26) |  |  |
| MCS * Highest tertile / Least deprived | 0.07 (-0.10, 0.24) |  |  | 0.16 (-0.01, 0.33) |  |  | 0.04 (-0.13, 0.21) |  |  | -0.03 (-0.25, 0.18) |  |  |

*Note.* Adjusted models included birth sex; prospectively assessed self-rated health, long-standing illness, psychological distress, and life satisfaction (life satisfaction only in models for that outcome); and household composition as covariates. Sensitivity models correspond to the unadjusted models after restricting the analytical sample to that of the adjusted models. BCS: British Cohort Study, 1970 birth cohort; GAD-2: 2-item General Anxiety Disorder questionnaire; IMD: index of multiple deprivation; MCS: Millennium Cohort Study, 2000 birth cohort; NCDS: National Child and Development Study, 1958 birth cohort; NS: Next Steps, 1990 cohort; NSHD: National Survey of Health and Development, 1946 birth cohort; ONS: UK Office for National Statistics; PHQ-2: 2-item Patient Health Questionnaire; UCLA-3: 3-item UCLA loneliness scale. χ2: Wald test performed to assess the overall statistical significance of the interaction terms; χ2 statistics have 4 degrees of freedom for time*cohort interaction terms, 2 degrees of freedom for time*IMD rank interaction terms; and 8 degrees of freedom for the rest of interaction terms.

## TableS8.2. Unadjusted and adjusted marginal mean estimates and 95% confidence intervals by IMD tertile.

|  |  |  | **GAD-2, anxiety symptomatology** | **PHQ-2, depressive symptomatology** | **UCLA-3 (feelings of loneliness)** | **Life satisfaction** |
| --- | --- | --- | --- | --- | --- | --- |
| **Cohort** | **IMD rank tertile** | **Survey wave** | **Unadjusted marginal mean (95% CI)** | **Unadjusted marginal mean (95% CI)** | **Unadjusted marginal mean (95% CI)** | **Unadjusted marginal mean (95% CI)** |
| NSHD | Lowest tertile / most deprived | 1 | 0.43 (0.31, 0.55) | 0.43 (0.30, 0.55) | 4.03 (3.85, 4.20) | 7.70 (7.45, 7.95) |
| NSHD | Lowest tertile / most deprived | 2 | 0.60 (0.47, 0.73) | 0.48 (0.35, 0.60) | 3.89 (3.73, 4.06) | 7.48 (7.26, 7.70) |
| NSHD | Lowest tertile / most deprived | 3 | 0.56 (0.43, 0.69) | 0.57 (0.43, 0.71) | 4.18 (4.00, 4.36) | 7.09 (6.83, 7.34) |
| NSHD | Middle tertile | 1 | 0.53 (0.43, 0.62) | 0.47 (0.39, 0.56) | 3.93 (3.80, 4.06) | 7.56 (7.35, 7.76) |
| NSHD | Middle tertile | 2 | 0.60 (0.51, 0.70) | 0.42 (0.34, 0.49) | 3.90 (3.78, 4.03) | 7.56 (7.39, 7.73) |
| NSHD | Middle tertile | 3 | 0.58 (0.49, 0.68) | 0.51 (0.43, 0.60) | 4.12 (3.98, 4.25) | 7.08 (6.89, 7.26) |
| NSHD | Highest tertile / least deprived | 1 | 0.50 (0.40, 0.59) | 0.45 (0.36, 0.55) | 4.01 (3.89, 4.14) | 7.49 (7.30, 7.68) |
| NSHD | Highest tertile / least deprived | 2 | 0.69 (0.60, 0.79) | 0.58 (0.50, 0.66) | 3.78 (3.68, 3.88) | 7.33 (7.18, 7.49) |
| NSHD | Highest tertile / least deprived | 3 | 0.64 (0.55, 0.74) | 0.58 (0.50, 0.67) | 4.15 (4.03, 4.27) | 6.81 (6.64, 6.98) |
| NCDS | Lowest tertile / most deprived | 1 | 0.83 (0.76, 0.90) | 0.81 (0.74, 0.87) | 4.29 (4.21, 4.38) | 7.34 (7.23, 7.45) |
| NCDS | Lowest tertile / most deprived | 2 | 0.86 (0.79, 0.93) | 0.83 (0.77, 0.90) | 4.20 (4.12, 4.27) | 7.27 (7.17, 7.37) |
| NCDS | Lowest tertile / most deprived | 3 | 0.88 (0.82, 0.94) | 0.98 (0.91, 1.04) | 4.48 (4.40, 4.55) | 6.88 (6.79, 6.98) |
| NCDS | Middle tertile | 1 | 0.75 (0.68, 0.81) | 0.62 (0.56, 0.68) | 4.12 (4.05, 4.19) | 7.56 (7.47, 7.65) |
| NCDS | Middle tertile | 2 | 0.80 (0.74, 0.85) | 0.65 (0.60, 0.70) | 4.01 (3.94, 4.07) | 7.42 (7.34, 7.51) |
| NCDS | Middle tertile |  | 0.77 (0.71, 0.82) | 0.79 (0.74, 0.84) | 4.30 (4.23, 4.36) | 6.99 (6.91, 7.08) |
| NCDS | Highest tertile / least deprived | 1 | 0.68 (0.62, 0.74) | 0.60 (0.55, 0.65) | 4.04 (3.97, 4.10) | 7.47 (7.38, 7.56) |
| NCDS | Highest tertile / least deprived | 2 | 0.75 (0.69, 0.80) | 0.59 (0.54, 0.64) | 3.95 (3.89, 4.01) | 7.47 (7.39, 7.54) |
| NCDS | Highest tertile / least deprived | 3 | 0.71 (0.66, 0.76) | 0.67 (0.62, 0.72) | 4.19 (4.13, 4.26) | 6.97 (6.88, 7.05) |
| BCS70 | Lowest tertile / most deprived | 1 | 1.08 (0.99, 1.16) | 1.06 (0.98, 1.14) | 4.35 (4.26, 4.44) | 7.06 (6.95, 7.17) |
| BCS70 | Lowest tertile / most deprived | 2 | 1.22 (1.14, 1.30) | 1.10 (1.03, 1.18) | 4.47 (4.38, 4.56) | 6.86 (6.75, 6.96) |
| BCS70 | Lowest tertile / most deprived | 3 | 1.16 (1.08, 1.24) | 1.26 (1.18, 1.33) | 4.61 (4.52, 4.69) | 6.58 (6.47, 6.69) |
| BCS70 | Middle tertile | 1 | 0.96 (0.88, 1.03) | 0.87 (0.80, 0.94) | 4.20 (4.12, 4.28) | 7.24 (7.14, 7.34) |
| BCS70 | Middle tertile | 2 | 1.02 (0.95, 1.09) | 0.83 (0.77, 0.90) | 4.18 (4.11, 4.26) | 7.18 (7.09, 7.27) |
| BCS70 | Middle tertile | 3 | 1.00 (0.93, 1.06) | 0.97 (0.91, 1.03) | 4.37 (4.30, 4.44) | 6.87 (6.78, 6.96) |
| BCS70 | Highest tertile / least deprived | 1 | 0.82 (0.75, 0.88) | 0.78 (0.72, 0.85) | 4.11 (4.04, 4.19) | 7.28 (7.18, 7.38) |
| BCS70 | Highest tertile / least deprived | 2 | 0.94 (0.88, 1.00) | 0.74 (0.68, 0.80) | 4.08 (4.01, 4.15) | 7.24 (7.16, 7.33) |
| BCS70 | Highest tertile / least deprived | 3 | 0.90 (0.84, 0.96) | 0.91 (0.85, 0.97) | 4.29 (4.22, 4.36) | 6.83 (6.75, 6.92) |
| NS | Lowest tertile / most deprived | 1 | 1.54 (1.43, 1.66) | 1.53 (1.42, 1.64) | 4.73 (4.62, 4.83) | 6.71 (6.57, 6.85) |
| NS | Lowest tertile / most deprived | 2 | 1.64 (1.55, 1.74) | 1.39 (1.30, 1.47) | 4.79 (4.70, 4.88) | 6.75 (6.64, 6.86) |
| NS | Lowest tertile / most deprived | 3 | 1.63 (1.54, 1.71) | 1.52 (1.44, 1.59) | 4.91 (4.83, 4.99) | 6.41 (6.31, 6.51) |
| NS | Middle tertile | 1 | 1.50 (1.37, 1.64) | 1.45 (1.31, 1.58) | 4.58 (4.45, 4.71) | 6.82 (6.64, 6.99) |
| NS | Middle tertile | 2 | 1.62 (1.52, 1.73) | 1.31 (1.21, 1.41) | 4.57 (4.47, 4.67) | 6.92 (6.79, 7.05) |
| NS | Middle tertile | 3 | 1.67 (1.57, 1.77) | 1.50 (1.41, 1.60) | 4.80 (4.70, 4.89) | 6.57 (6.45, 6.69) |
| NS | Highest tertile / least deprived | 1 | 1.32 (1.19, 1.45) | 1.24 (1.11, 1.36) | 4.51 (4.37, 4.65) | 6.99 (6.83, 7.16) |
| NS | Highest tertile / least deprived | 2 | 1.54 (1.43, 1.66) | 1.16 (1.06, 1.26) | 4.53 (4.42, 4.63) | 7.15 (7.03, 7.28) |
| NS | Highest tertile / least deprived | 3 | 1.56 (1.46, 1.67) | 1.44 (1.34, 1.54) | 4.73 (4.62, 4.83) | 6.66 (6.54, 6.79) |
| MCS | Lowest tertile / most deprived | 1 | 1.80 (1.68, 1.92) | 1.99 (1.87, 2.10) | 5.19 (5.08, 5.30) | 5.96 (5.81, 6.11) |
| MCS | Lowest tertile / most deprived | 2 | 2.12 (2.00, 2.23) | 1.85 (1.74, 1.96) | 5.23 (5.12, 5.34) | 6.21 (6.09, 6.34) |
| MCS | Lowest tertile / most deprived | 3 | 2.13 (2.04, 2.23) | 2.11 (2.02, 2.19) | 5.27 (5.18, 5.36) | 5.89 (5.77, 6.00) |
| MCS | Middle tertile | 1 | 1.76 (1.63, 1.89) | 1.83 (1.71, 1.96) | 5.16 (5.03, 5.29) | 6.36 (6.20, 6.52) |
| MCS | Middle tertile | 2 | 2.07 (1.95, 2.19) | 1.69 (1.58, 1.80) | 5.23 (5.11, 5.35) | 6.38 (6.24, 6.51) |
| MCS | Middle tertile | 3 | 2.22 (2.12, 2.32) | 2.00 (1.91, 2.10) | 5.33 (5.22, 5.43) | 5.93 (5.81, 6.05) |
| MCS | Highest tertile / least deprived | 1 | 1.74 (1.62, 1.86) | 1.83 (1.71, 1.94) | 5.14 (5.02, 5.26) | 6.21 (6.05, 6.37) |
| MCS | Highest tertile / least deprived | 2 | 2.01 (1.89, 2.12) | 1.51 (1.41, 1.62) | 5.13 (5.02, 5.24) | 6.51 (6.38, 6.64) |
| MCS | Highest tertile / least deprived | 3 | 1.94 (1.84, 2.04) | 1.82 (1.73, 1.91) | 5.18 (5.08, 5.28) | 6.02 (5.91, 6.14) |
| **Cohort** | **IMD rank tertile** | **Survey wave** | **Adjusted marginal mean (95% CI)** | **Adjusted marginal mean (95% CI)** | **Adjusted marginal mean (95% CI)** | **Adjusted marginal mean (95% CI)** |
| NSHD | Lowest tertile / most deprived | 1 | 0.42 (0.30, 0.54) | 0.41 (0.29, 0.54) | 4.08 (3.90, 4.26) | 7.45 (7.20, 7.70) |
| NSHD | Lowest tertile / most deprived | 2 | 0.60 (0.48, 0.73) | 0.47 (0.34, 0.61) | 3.99 (3.82, 4.17) | 7.35 (7.11, 7.59) |
| NSHD | Lowest tertile / most deprived | 3 | 0.56 (0.42, 0.70) | 0.63 (0.47, 0.79) | 4.27 (4.08, 4.47) | 6.68 (6.37, 7.00) |
| NSHD | Middle tertile | 1 | 0.57 (0.46, 0.67) | 0.58 (0.49, 0.67) | 4.10 (3.96, 4.24) | 7.29 (7.08, 7.50) |
| NSHD | Middle tertile | 2 | 0.65 (0.55, 0.75) | 0.49 (0.41, 0.58) | 4.05 (3.91, 4.19) | 7.17 (6.97, 7.37) |
| NSHD | Middle tertile | 3 | 0.62 (0.51, 0.73) | 0.59 (0.49, 0.68) | 4.26 (4.12, 4.41) | 6.77 (6.55, 6.98) |
| NSHD | Highest tertile / least deprived | 1 | 0.53 (0.43, 0.62) | 0.52 (0.42, 0.62) | 4.21 (4.08, 4.34) | 7.05 (6.85, 7.26) |
| NSHD | Highest tertile / least deprived | 2 | 0.72 (0.62, 0.82) | 0.64 (0.55, 0.72) | 3.97 (3.86, 4.07) | 6.97 (6.79, 7.15) |
| NSHD | Highest tertile / least deprived | 3 | 0.66 (0.56, 0.76) | 0.65 (0.56, 0.73) | 4.33 (4.20, 4.45) | 6.47 (6.27, 6.67) |
| NCDS | Lowest tertile / most deprived | 1 | 0.76 (0.69, 0.83) | 0.74 (0.67, 0.81) | 4.27 (4.18, 4.35) | 7.32 (7.22, 7.43) |
| NCDS | Lowest tertile / most deprived | 2 | 0.79 (0.72, 0.85) | 0.74 (0.67, 0.80) | 4.16 (4.09, 4.24) | 7.31 (7.22, 7.41) |
| NCDS | Lowest tertile / most deprived | 3 | 0.81 (0.75, 0.87) | 0.92 (0.85, 0.99) | 4.45 (4.37, 4.53) | 6.91 (6.81, 7.01) |
| NCDS | Middle tertile | 1 | 0.79 (0.73, 0.85) | 0.69 (0.63, 0.74) | 4.24 (4.17, 4.31) | 7.37 (7.28, 7.46) |
| NCDS | Middle tertile | 2 | 0.85 (0.79, 0.90) | 0.71 (0.66, 0.76) | 4.14 (4.08, 4.21) | 7.23 (7.15, 7.31) |
| NCDS | Middle tertile | 3 | 0.83 (0.78, 0.89) | 0.86 (0.81, 0.92) | 4.42 (4.35, 4.49) | 6.83 (6.74, 6.91) |
| NCDS | Highest tertile / least deprived | 1 | 0.79 (0.73, 0.85) | 0.73 (0.67, 0.78) | 4.25 (4.18, 4.32) | 7.18 (7.09, 7.27) |
| NCDS | Highest tertile / least deprived | 2 | 0.87 (0.82, 0.93) | 0.71 (0.66, 0.76) | 4.15 (4.09, 4.22) | 7.19 (7.12, 7.27) |
| NCDS | Highest tertile / least deprived | 3 | 0.83 (0.78, 0.88) | 0.81 (0.76, 0.86) | 4.38 (4.32, 4.45) | 6.67 (6.58, 6.75) |
| BCS70 | Lowest tertile / most deprived | 1 | 0.95 (0.87, 1.04) | 0.96 (0.88, 1.04) | 4.28 (4.19, 4.37) | 7.10 (6.99, 7.21) |
| BCS70 | Lowest tertile / most deprived | 2 | 1.09 (1.01, 1.17) | 0.97 (0.89, 1.05) | 4.39 (4.31, 4.48) | 6.94 (6.84, 7.04) |
| BCS70 | Lowest tertile / most deprived | 3 | 0.96 (0.88, 1.03) | 1.08 (1.00, 1.15) | 4.47 (4.39, 4.56) | 6.67 (6.57, 6.78) |
| BCS70 | Middle tertile | 1 | 0.95 (0.88, 1.03) | 0.92 (0.85, 0.99) | 4.32 (4.24, 4.40) | 7.05 (6.95, 7.15) |
| BCS70 | Middle tertile | 2 | 1.02 (0.95, 1.09) | 0.87 (0.80, 0.93) | 4.29 (4.21, 4.37) | 7.00 (6.90, 7.09) |
| BCS70 | Middle tertile | 3 | 0.98 (0.91, 1.05) | 1.01 (0.94, 1.07) | 4.48 (4.40, 4.56) | 6.70 (6.61, 6.79) |
| BCS70 | Highest tertile / least deprived | 1 | 0.88 (0.81, 0.95) | 0.90 (0.83, 0.97) | 4.35 (4.27, 4.43) | 6.97 (6.88, 7.07) |
| BCS70 | Highest tertile / least deprived | 2 | 1.00 (0.93, 1.07) | 0.84 (0.78, 0.90) | 4.31 (4.24, 4.39) | 6.96 (6.87, 7.05) |
| BCS70 | Highest tertile / least deprived | 3 | 0.96 (0.90, 1.02) | 1.01 (0.94, 1.07) | 4.50 (4.43, 4.57) | 6.57 (6.48, 6.67) |
| NS | Lowest tertile / most deprived | 1 | 1.49 (1.37, 1.61) | 1.50 (1.38, 1.61) | 4.70 (4.59, 4.80) | 6.76 (6.62, 6.91) |
| NS | Lowest tertile / most deprived | 2 | 1.57 (1.48, 1.67) | 1.35 (1.26, 1.44) | 4.76 (4.67, 4.85) | 6.81 (6.70, 6.92) |
| NS | Lowest tertile / most deprived | 3 | 1.54 (1.45, 1.62) | 1.44 (1.36, 1.52) | 4.88 (4.80, 4.96) | 6.48 (6.37, 6.58) |
| NS | Middle tertile | 1 | 1.41 (1.28, 1.55) | 1.41 (1.28, 1.55) | 4.66 (4.53, 4.79) | 6.70 (6.52, 6.87) |
| NS | Middle tertile | 2 | 1.57 (1.46, 1.68) | 1.32 (1.21, 1.42) | 4.63 (4.53, 4.73) | 6.82 (6.69, 6.94) |
| NS | Middle tertile | 3 | 1.59 (1.48, 1.69) | 1.48 (1.39, 1.57) | 4.82 (4.72, 4.91) | 6.49 (6.37, 6.61) |
| NS | Highest tertile / least deprived | 1 | 1.27 (1.14, 1.39) | 1.22 (1.10, 1.34) | 4.57 (4.44, 4.70) | 6.84 (6.69, 7.00) |
| NS | Highest tertile / least deprived | 2 | 1.51 (1.40, 1.62) | 1.16 (1.06, 1.26) | 4.57 (4.46, 4.67) | 7.03 (6.91, 7.15) |
| NS | Highest tertile / least deprived | 3 | 1.48 (1.38, 1.59) | 1.41 (1.31, 1.51) | 4.78 (4.68, 4.89) | 6.57 (6.45, 6.68) |
| MCS | Lowest tertile / most deprived | 1 | 1.70 (1.58, 1.83) | 1.88 (1.77, 2.00) | 4.88 (4.76, 4.99) | 6.50 (6.34, 6.65) |
| MCS | Lowest tertile / most deprived | 2 | 2.05 (1.93, 2.17) | 1.78 (1.66, 1.89) | 4.93 (4.81, 5.05) | 6.74 (6.60, 6.88) |
| MCS | Lowest tertile / most deprived | 3 | 2.04 (1.94, 2.14) | 2.02 (1.92, 2.11) | 4.97 (4.87, 5.07) | 6.39 (6.26, 6.52) |
| MCS | Middle tertile | 1 | 1.76 (1.63, 1.89) | 1.85 (1.72, 1.98) | 4.93 (4.80, 5.07) | 6.75 (6.59, 6.92) |
| MCS | Middle tertile | 2 | 2.10 (1.97, 2.22) | 1.75 (1.63, 1.87) | 5.04 (4.91, 5.17) | 6.71 (6.56, 6.85) |
| MCS | Middle tertile | 3 | 2.25 (2.14, 2.36) | 2.03 (1.92, 2.14) | 5.13 (5.01, 5.24) | 6.31 (6.17, 6.44) |
| MCS | Highest tertile / least deprived | 1 | 1.78 (1.66, 1.90) | 1.87 (1.75, 1.98) | 4.94 (4.81, 5.07) | 6.58 (6.42, 6.74) |
| MCS | Highest tertile / least deprived | 2 | 2.05 (1.93, 2.17) | 1.57 (1.46, 1.68) | 4.96 (4.84, 5.08) | 6.86 (6.72, 7.00) |
| MCS | Highest tertile / least deprived | 3 | 2.00 (1.90, 2.10) | 1.88 (1.78, 1.98) | 5.00 (4.89, 5.11) | 6.34 (6.21, 6.47) |

*Note.* Adjusted models included birth sex; prospectively assessed self-rated health, long-standing illness, psychological distress, and life satisfaction (life satisfaction only in models for that outcome); and household composition as covariates. BCS: British Cohort Study, 1970 birth cohort; GAD-2: 2-item General Anxiety Disorder questionnaire; IMD: index of multiple deprivation; MCS: Millennium Cohort Study, 2000 birth cohort; NCDS: National Child and Development Study, 1958 birth cohort; NS: Next Steps, 1990 cohort; NSHD: National Survey of Health and Development, 1946 birth cohort; ONS: UK Office for National Statistics; PHQ-2: 2-item Patient Health Questionnaire; UCLA-3: 3-item UCLA loneliness scale. Survey wave 1: May 2020; survey wave 2: September/October 2020; survey wave 3: February/March 2021.

## Table S8.3. Difference-in-differences (change in difference between people living in most vs least deprived IMD areas between first vs last time point) estimates and 95% confidence intervals.

|  | *DID* (95% CI) | *p* |
| --- | --- | --- |
| GAD-2 | 0.023 (-0.046, 0.093) | 0.513 |
| PHQ-2 | -0.014 (-0.084, 0.057) | 0.702 |
| UCLA-3 | -0.035 (-0.111, 0.040) | 0.358 |
| Life satisfaction | -0.003 (-0.119, 0.114) | 0.965 |

## Figure S8.1. Unadjusted and adjusted anxiety symptomatology (GAD-2) marginal mean estimates and 95% confidence intervals by IMD tertile.

Adjusted models include birth sex, pre-pandemic self-reported health and long-standing illness, pre-pandemic psychological distress, and household composition.


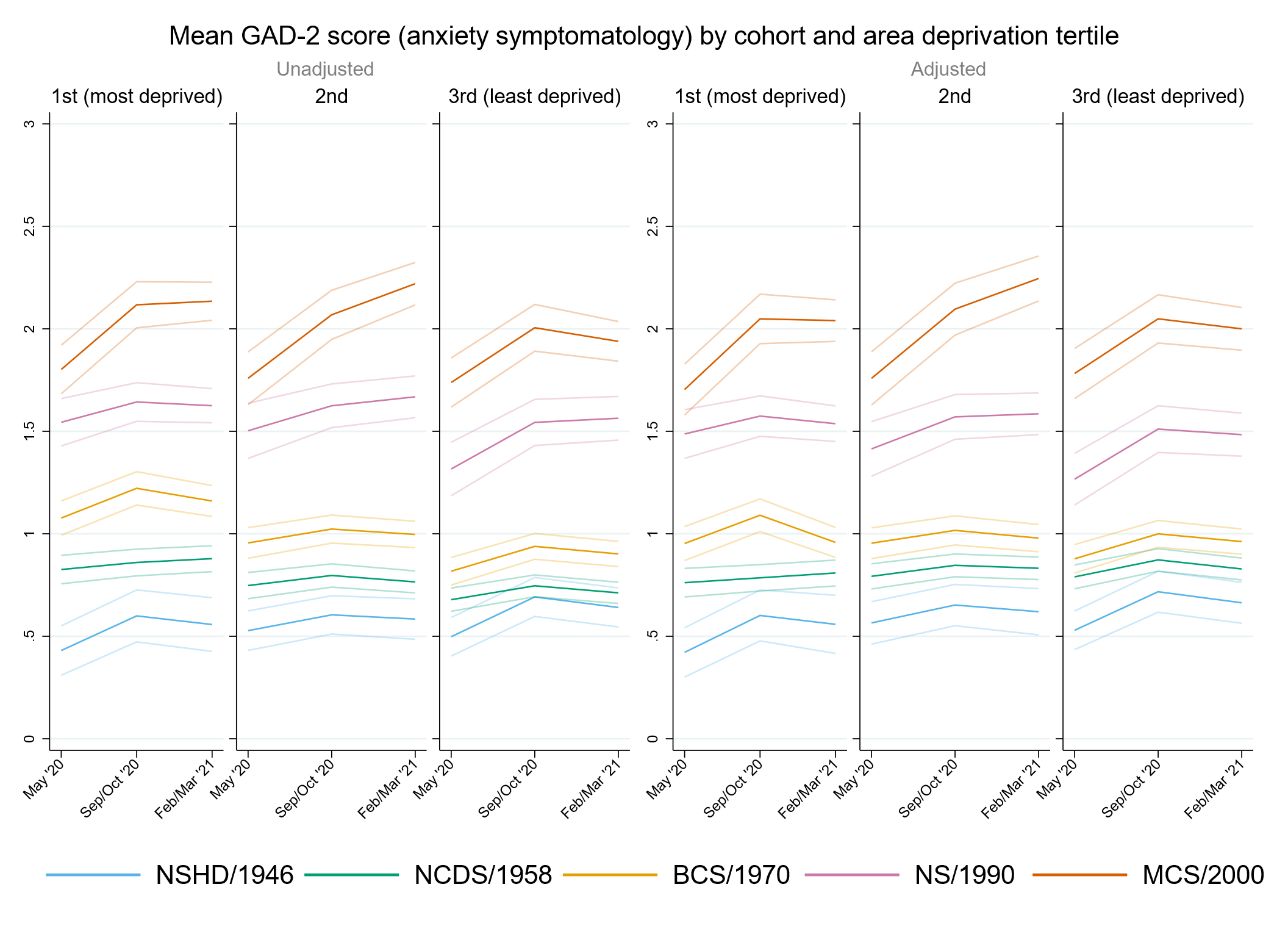


## Figure S8.2. Unadjusted and adjusted depressive symptomatology (PHQ-2) marginal mean estimates and 95% confidence intervals by IMD tertile.

Adjusted models include birth sex, pre-pandemic self-reported health and long-standing illness, pre-pandemic psychological distress, and household composition.


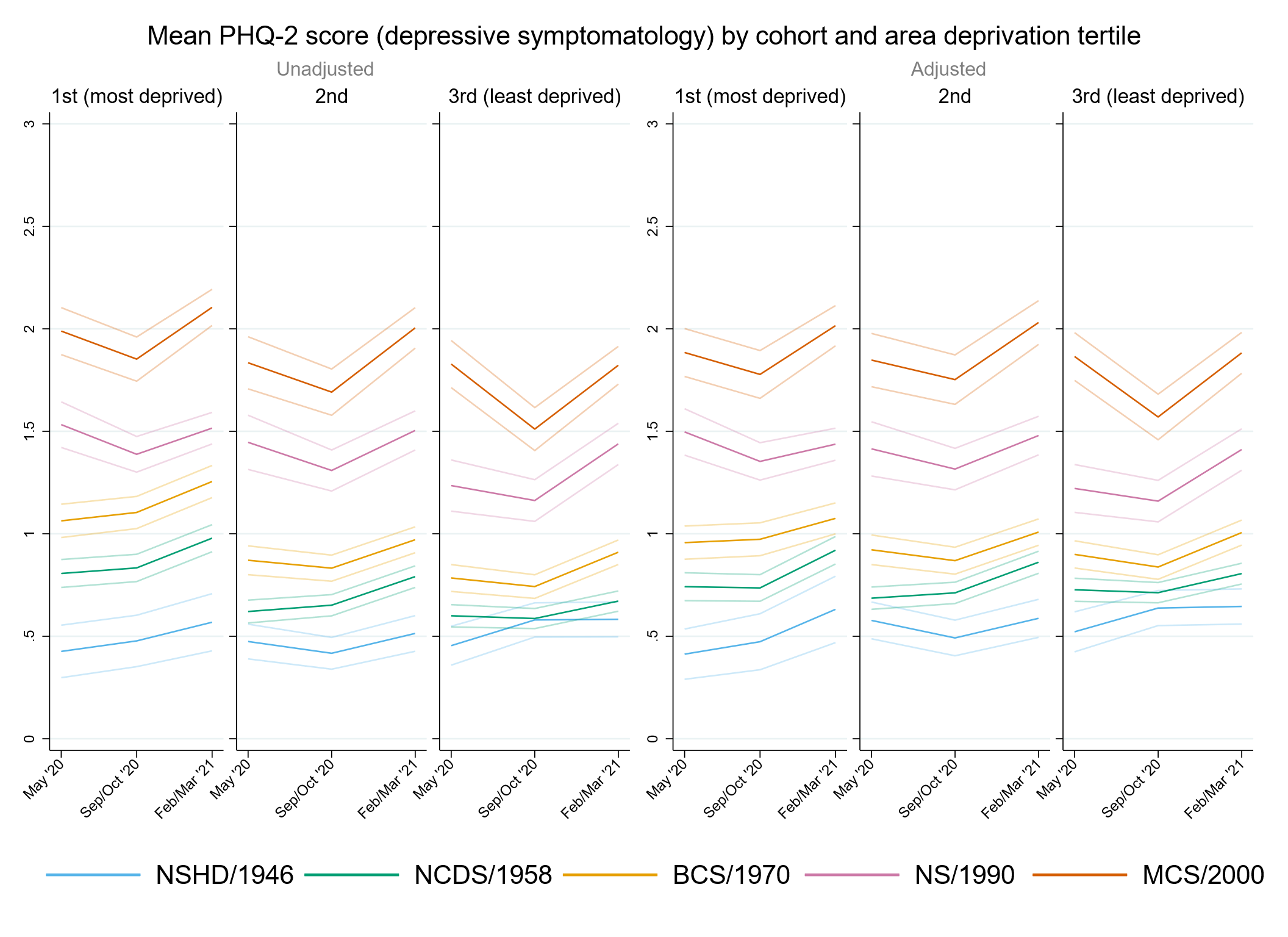


## Figure S8.3. Unadjusted and adjusted loneliness (UCLA-3) marginal mean estimates and 95% confidence intervals by IMD tertile.

Adjusted models include birth sex, pre-pandemic self-reported health and long-standing illness, pre-pandemic psychological distress, and household composition.


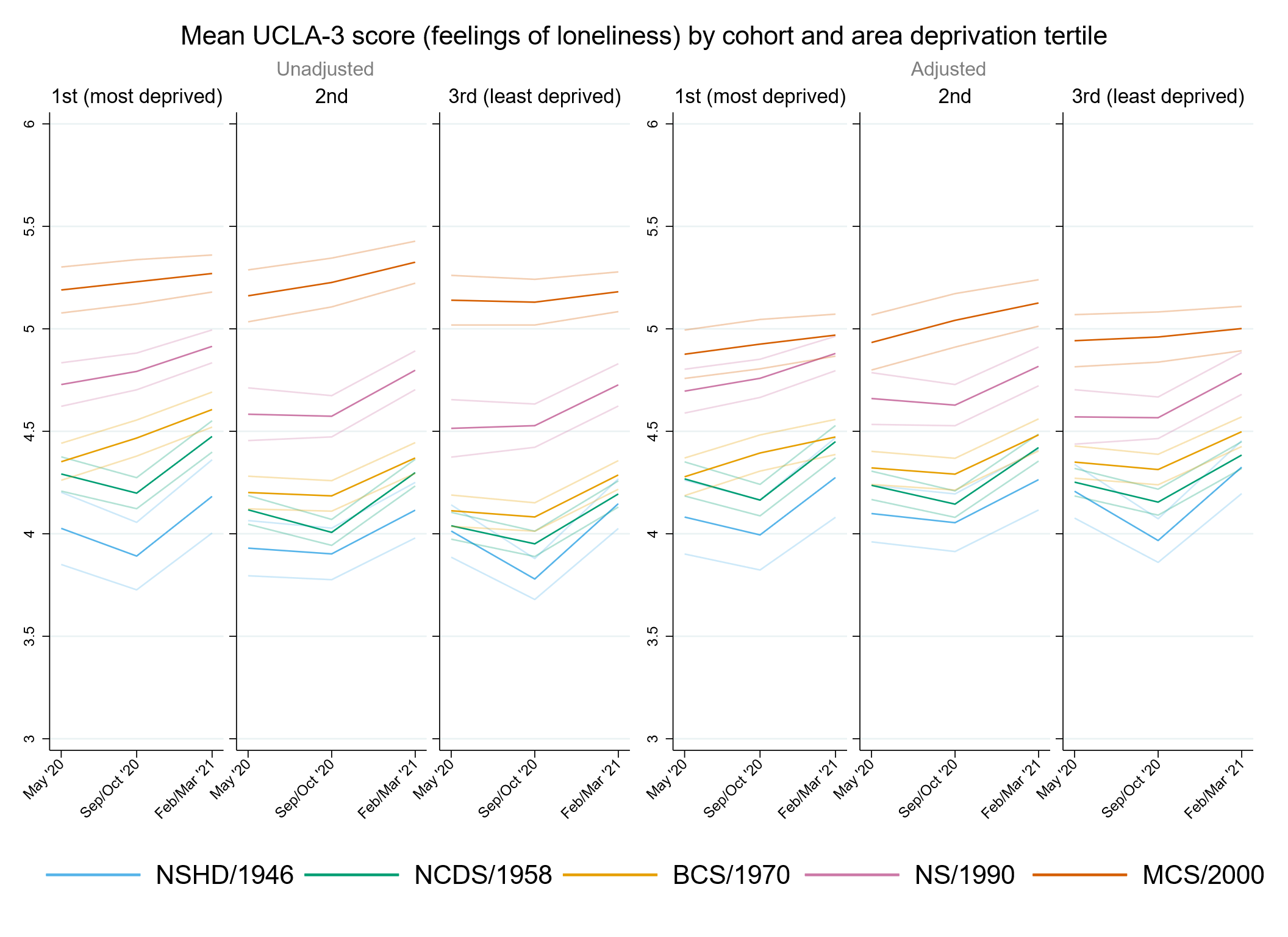


## Figure S8.4. Unadjusted and adjusted life satisfaction marginal mean estimates and 95% confidence intervals by IMD tertile.

Adjusted models include birth sex, pre-pandemic self-reported health and long-standing illness, pre-pandemic life satisfaction and psychological distress, and household composition.


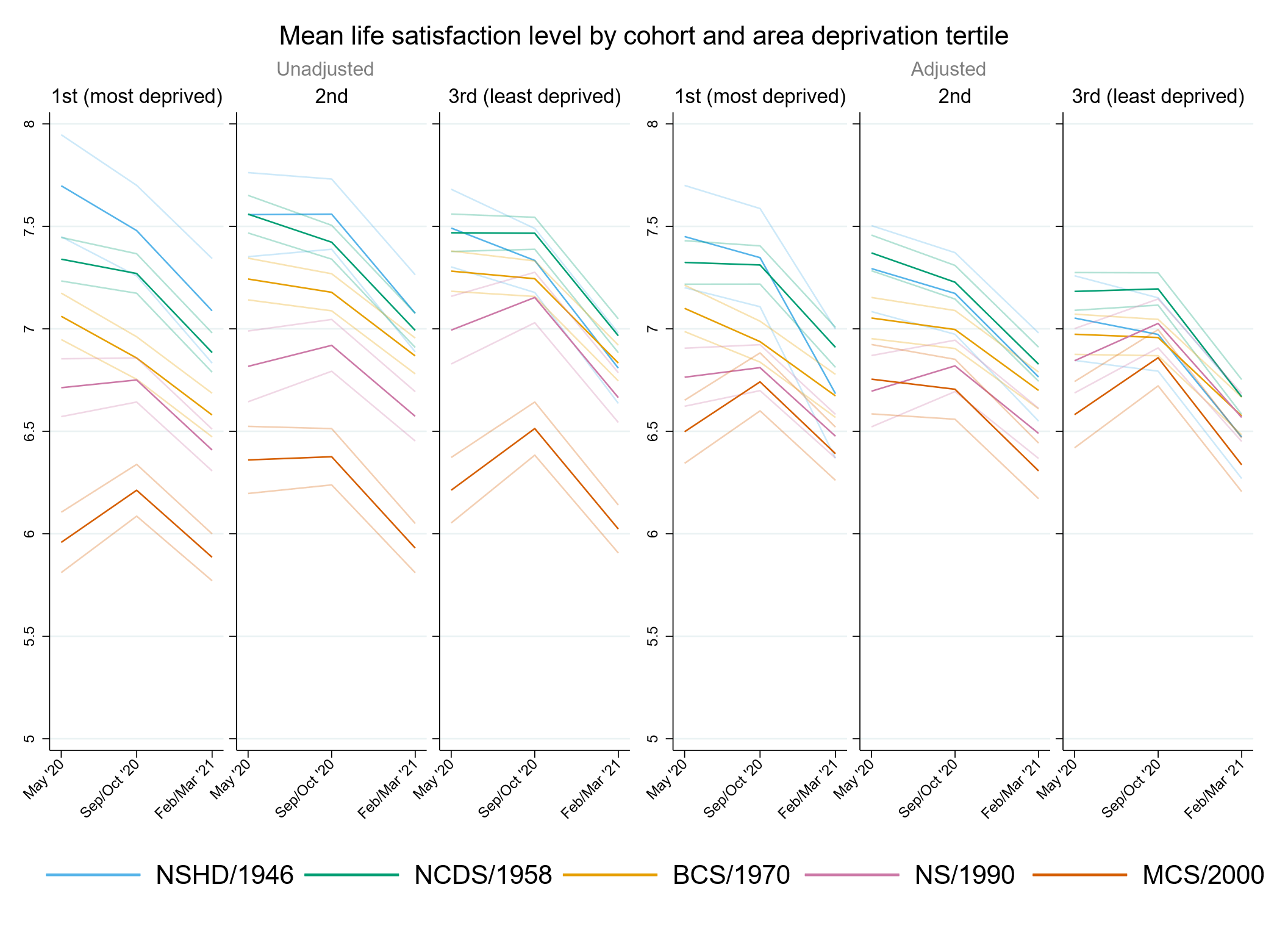


# Supplementary References

Blanchflower, D. G. (2021). Is happiness U-shaped everywhere? Age and subjective well-being in 145 countries. *Journal of Population Economics, 34*(2), 575-624. doi:10.1007/s00148-020-00797-z

De Stavola, B. L., Daniel, R. M., Ploubidis, G. B., & Micali, N. (2015). Mediation analysis with intermediate confounding: structural equation modeling viewed through the causal inference lens. *American Journal of Epidemiology, 181*(1), 64-80. doi:10.1093/aje/kwu239

Dodgeon, B., Morris, T., Crawford, C., Parsons, S., Vignoles, A., Oldfield, Z., & O'Neill, D. (2011). *CLOSER work package 2: Harmonised socio-economic measures user guide (revised)*. London: CLOSER.

Dodgeon, B., & Parsons, S. (2011). *Deriving highest qualification in NCDS and BCS70*. London: Centre for Longitudinal Studies, Institute of Education.

Gondek, D., Bann, D., Patalay, P., Goodman, A., McElroy, E., Richards, M., & Ploubidis, G. B. (2022). Psychological distress from early adulthood to early old age: evidence from the 1946, 1958 and 1970 British birth cohorts. *Psychological Medicine, 52*(8), 1471-1480. doi:10.1017/S003329172000327X

McElroy, E., Richards, M., Fitzsimons, E., Conti, G., Ploubidis, G. B., Sullivan, A., & Moulton, V. (2021). *Feasibility of retrospectively harmonising cognitive measures in five British birth cohort studies*. London: CLOSER.

Wood, N., Stafford, M., & O’Neill, D. (2019). *CLOSER work package 9: Harmonised childhood environment and adult wellbeing measures user guide.* London: CLOSER.
